# Supplementary figures and images for: ATG6 interacting with NPR1 increases Arabidopsis thaliana resistance to Pst DC3000/avrRps4 by increasing its nuclear accumulation and stability
Source: eLife. 2025 Mar 4;13:RP97206. doi: 10.7554/eLife.97206 (PMC11879114; doi:10.7554/eLife.97206)

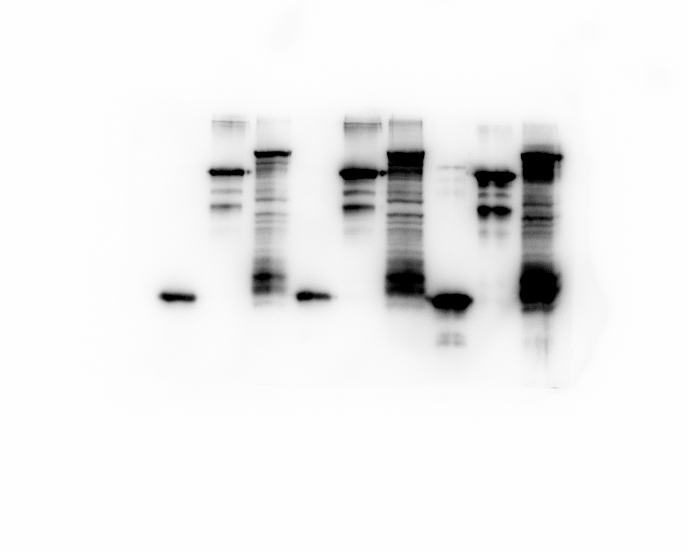

Supplement: Figure 1—source data 1. [file elife-97206-fig1-data1.zip › Figure 1-source data 1/Figure 1b/Original file for the Western blot analysis in Figure 1b (anti-GST,anti-GST-ATG6;anti-SnRK2.8) long exposure .tif]

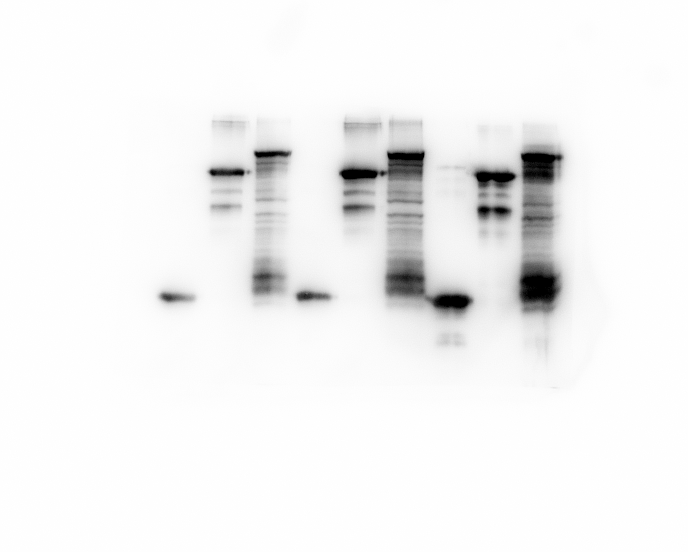

Supplement: Figure 1—source data 1. [file elife-97206-fig1-data1.zip › Figure 1-source data 1/Figure 1b/Original file for the Western blot analysis in Figure 1b (anti-GST,anti-GST-ATG6;anti-SnRK2.8) short exposure.tif]

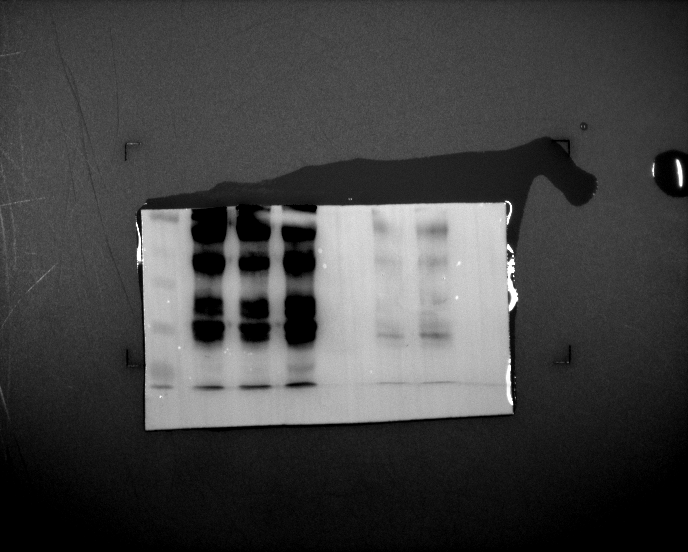

Supplement: Figure 1—source data 1. [file elife-97206-fig1-data1.zip › Figure 1-source data 1/Figure 1b/Original file for the Western blot analysis in Figure 1b (anti-NPR1-His) .tif]

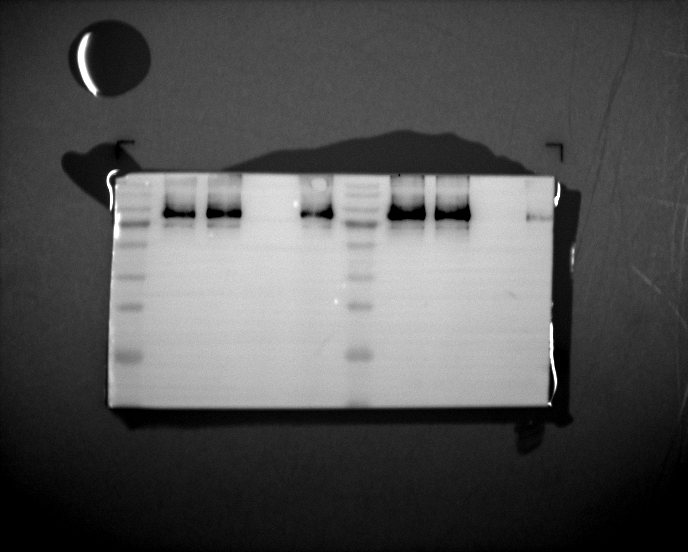

Supplement: Figure 1—source data 1. [file elife-97206-fig1-data1.zip › Figure 1-source data 1/Figure 1c/Original file for the Western blot analysis in Figure 1c (anti-ATG6-mCherry) .tif]

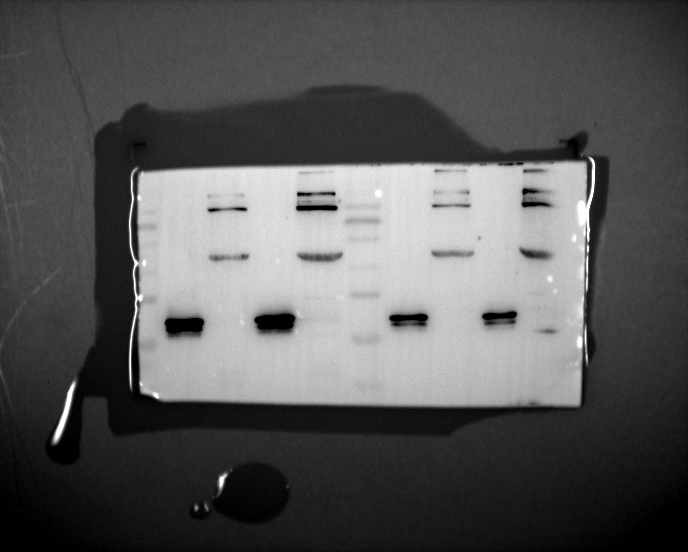

Supplement: Figure 1—source data 1. [file elife-97206-fig1-data1.zip › Figure 1-source data 1/Figure 1c/Original file for the Western blot analysis in Figure 1c (anti-NPR1-GFP and anti-GFP) .tif]

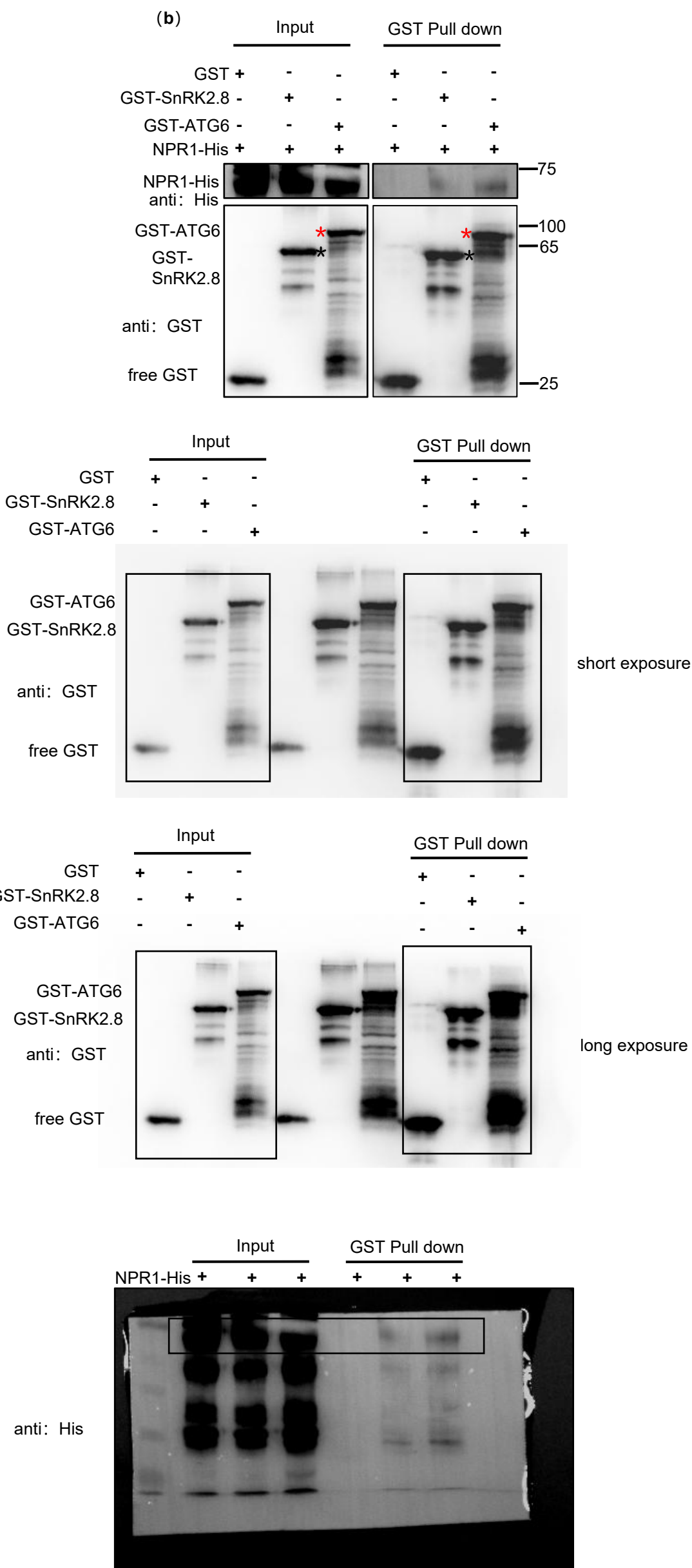

Figure 1. Physical interaction between NPR1 and ATG6.

Supplement: Figure 1—source data 2. [file elife-97206-fig1-data2.zip › Figure 1-source data 2/Figure 1b.pdf]

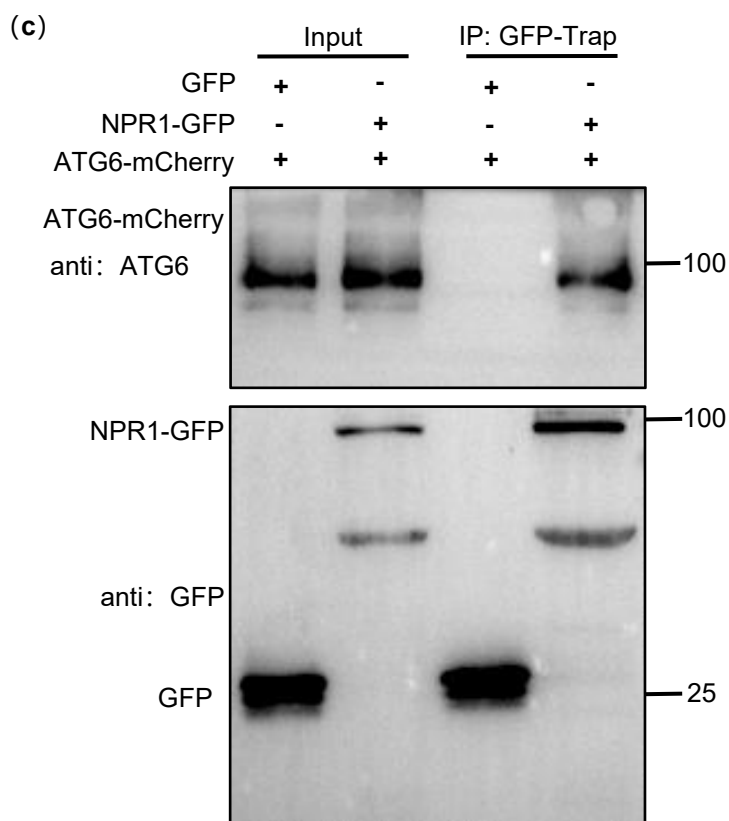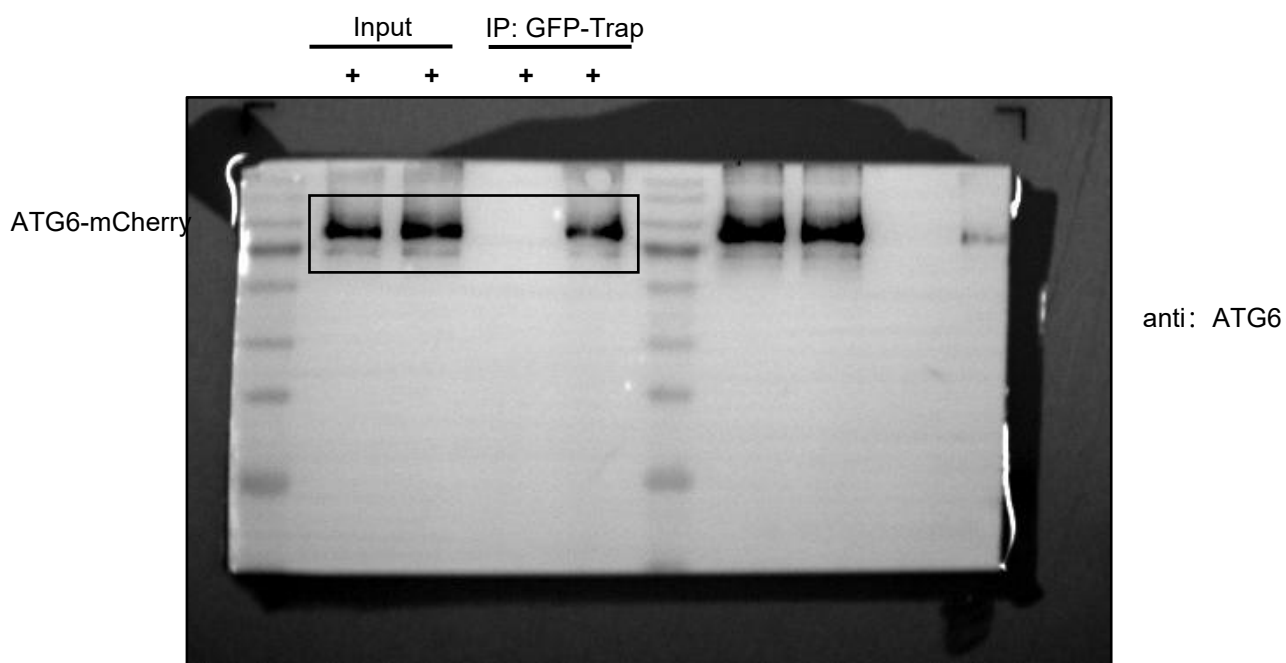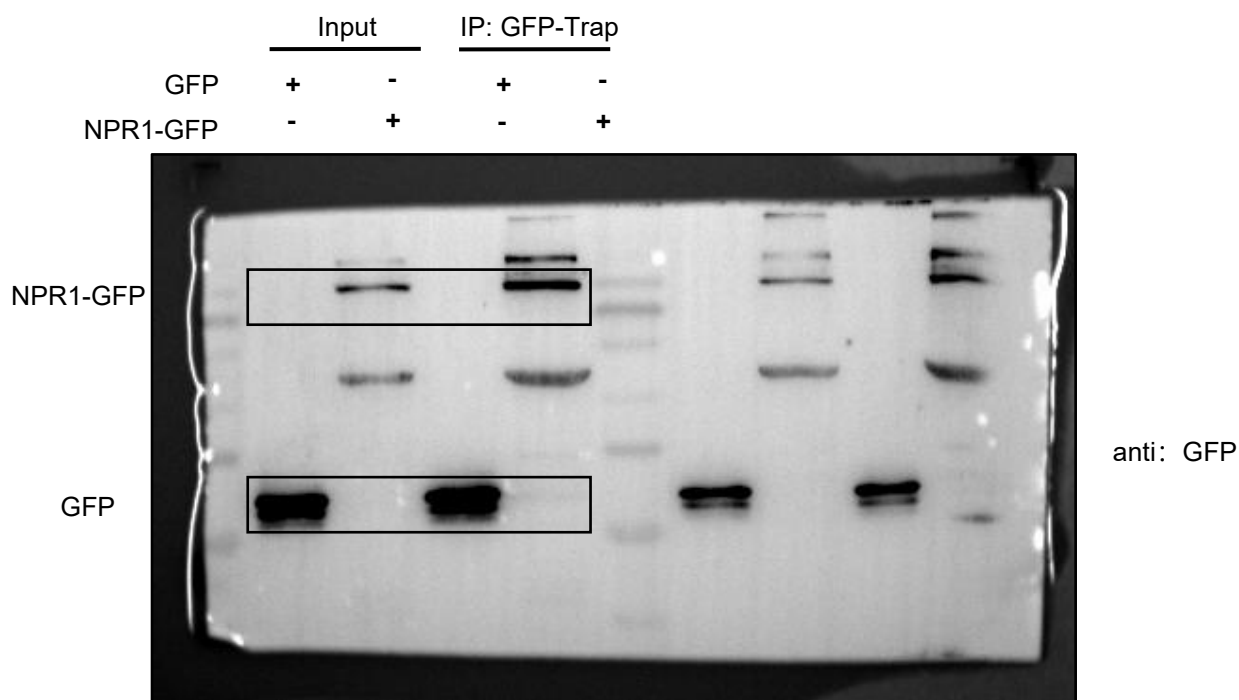

**Figure 1. Physical interaction between NPR1 and ATG6.**

Supplement: Figure 1—source data 2. [file elife-97206-fig1-data2.zip › Figure 1-source data 2/Figure 1c.pdf]

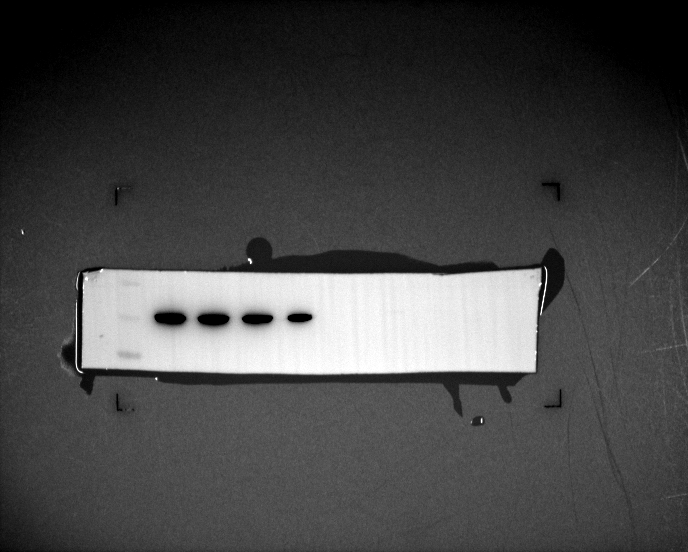

Supplement: Figure 2—source data 1. [file elife-97206-fig2-data1.zip › Figure 2-source data 1/Figure 2 d/Original file for the Western blot analysis in Figure 2d (anti-Actin ) .tif]

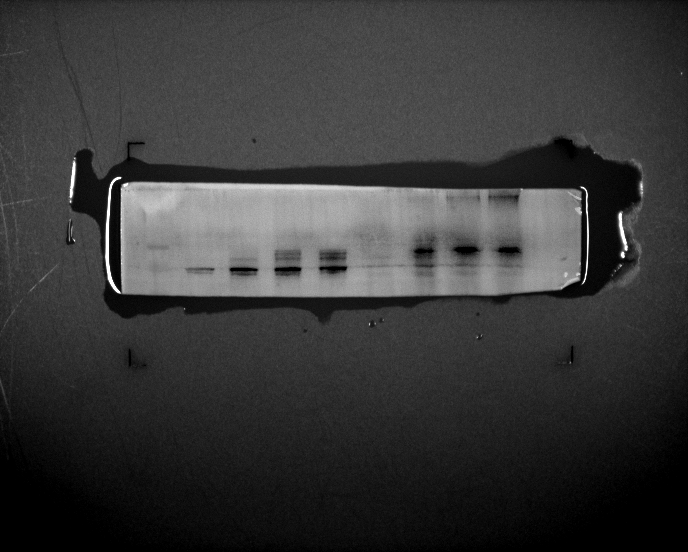

Supplement: Figure 2—source data 1. [file elife-97206-fig2-data1.zip › Figure 2-source data 1/Figure 2 d/Original file for the Western blot analysis in Figure 2d (anti-ATG6-mCherry ) .tif]

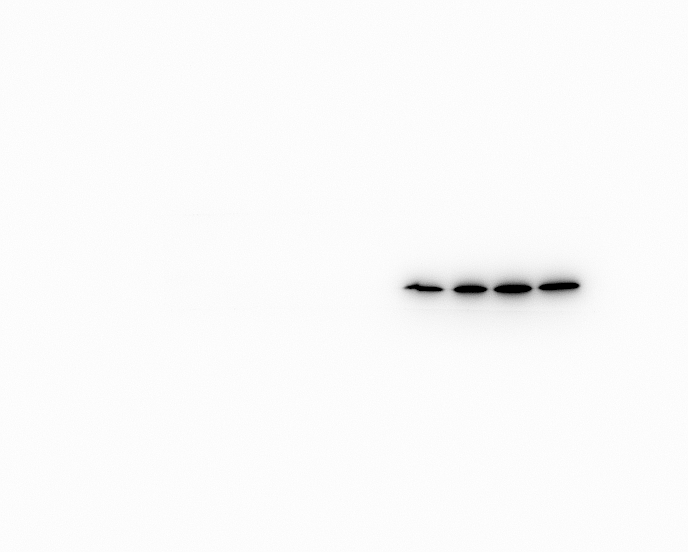

Supplement: Figure 2—source data 1. [file elife-97206-fig2-data1.zip › Figure 2-source data 1/Figure 2 d/Original file for the Western blot analysis in Figure 2d (anti-H3 ) .tif]

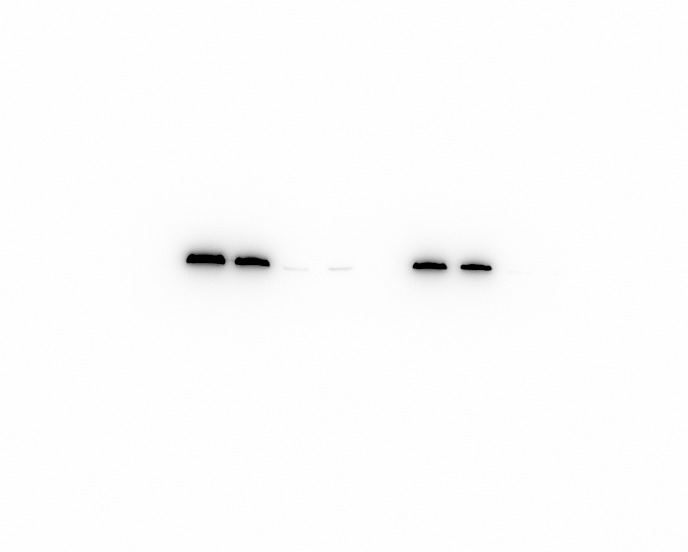

Supplement: Figure 2—source data 1. [file elife-97206-fig2-data1.zip › Figure 2-source data 1/Figure 2 e/Original file for the Western blot analysis in Figure 2e (anti-Actin ).tif]

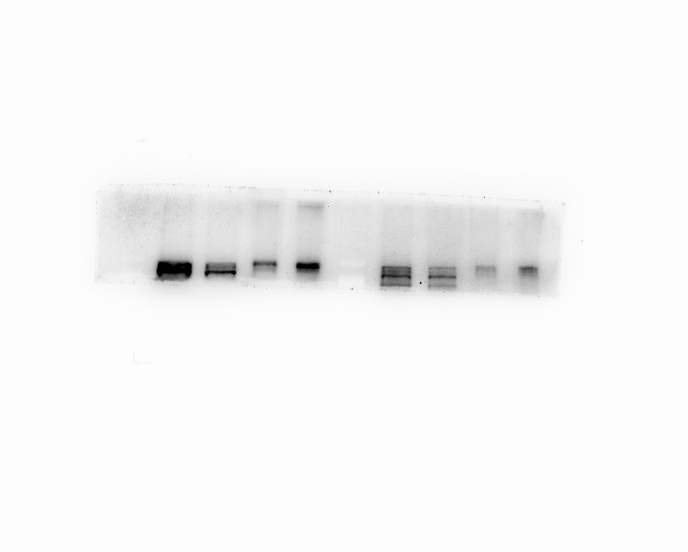

Supplement: Figure 2—source data 1. [file elife-97206-fig2-data1.zip › Figure 2-source data 1/Figure 2 e/Original file for the Western blot analysis in Figure 2e (anti-ATG6-GFP ) .tif]

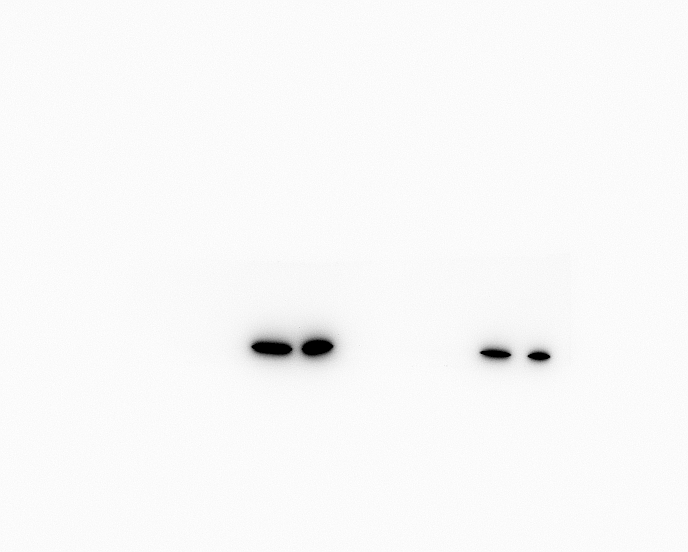

Supplement: Figure 2—source data 1. [file elife-97206-fig2-data1.zip › Figure 2-source data 1/Figure 2 e/Original file for the Western blot analysis in Figure 2e (antiH3 ).tif]

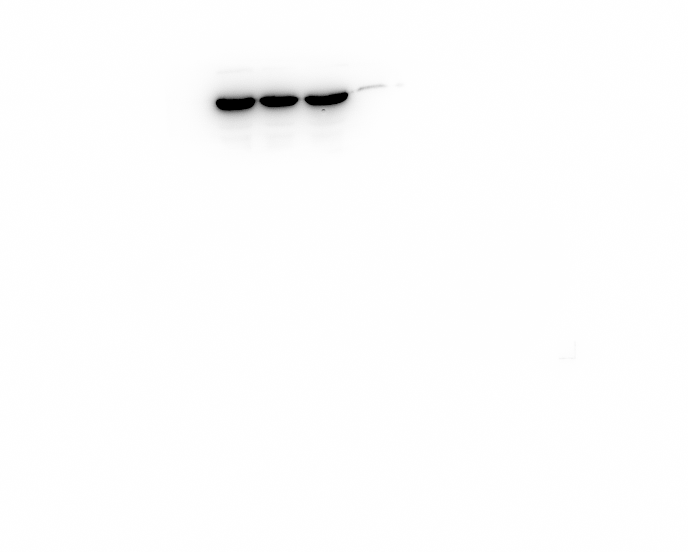

Supplement: Figure 2—source data 1. [file elife-97206-fig2-data1.zip › Figure 2-source data 1/Figure 2 f/Original file for the Western blot analysis in Figure 2f (anti-Actin ).tif]

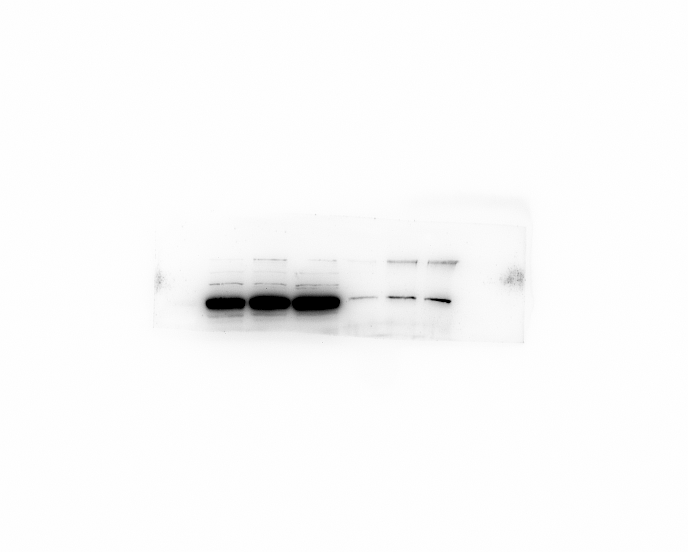

Supplement: Figure 2—source data 1. [file elife-97206-fig2-data1.zip › Figure 2-source data 1/Figure 2 f/Original file for the Western blot analysis in Figure 2f (anti-ATG6-GFP ).tif]

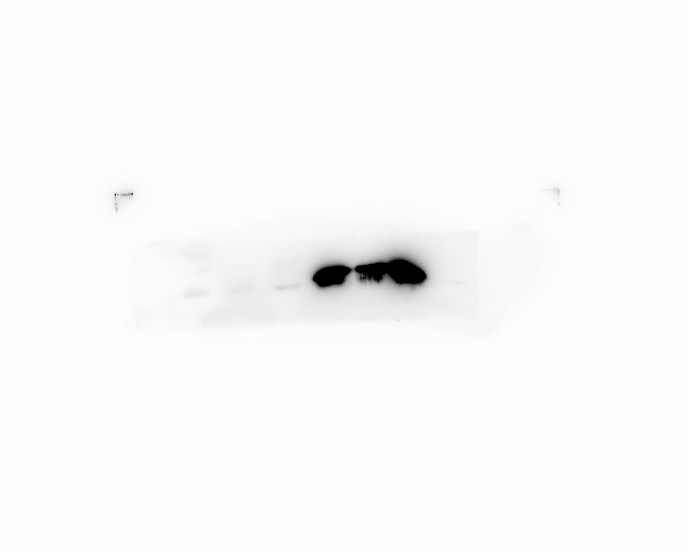

Supplement: Figure 2—source data 1. [file elife-97206-fig2-data1.zip › Figure 2-source data 1/Figure 2 f/Original file for the Western blot analysis in Figure 2f (anti-H3 ).tif]

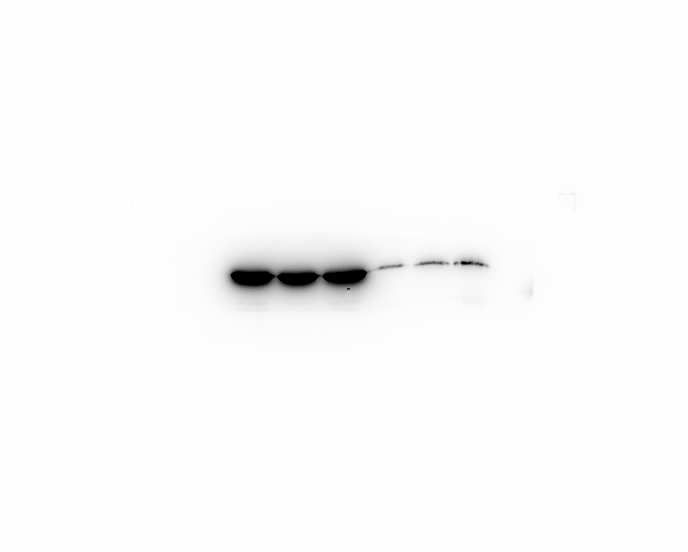

Supplement: Figure 2—source data 1. [file elife-97206-fig2-data1.zip › Figure 2-source data 1/Figure 2 g/Original file for the Western blot analysis in Figure 2g (anti-Actin ).tif]

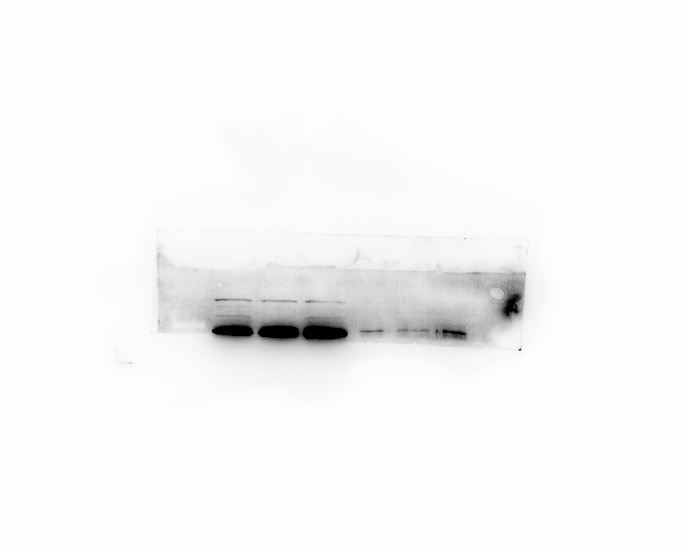

Supplement: Figure 2—source data 1. [file elife-97206-fig2-data1.zip › Figure 2-source data 1/Figure 2 g/Original file for the Western blot analysis in Figure 2g (anti-ATG6-mCherry ).tif]

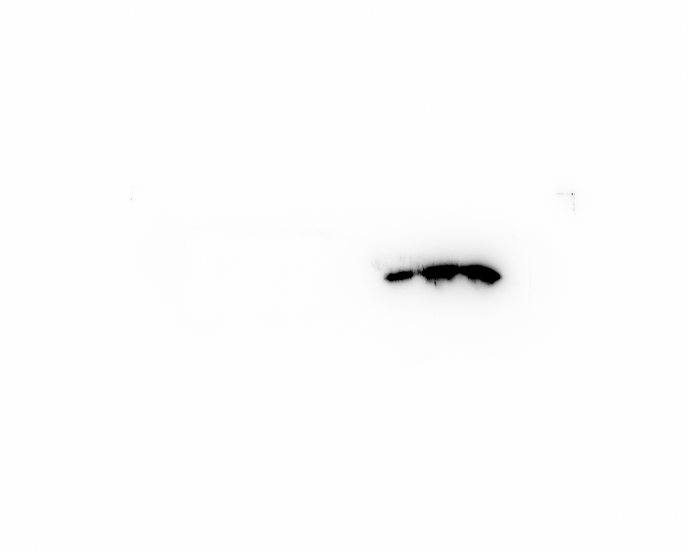

Supplement: Figure 2—source data 1. [file elife-97206-fig2-data1.zip › Figure 2-source data 1/Figure 2 g/Original file for the Western blot analysis in Figure 2g (anti-H3 ).tif]

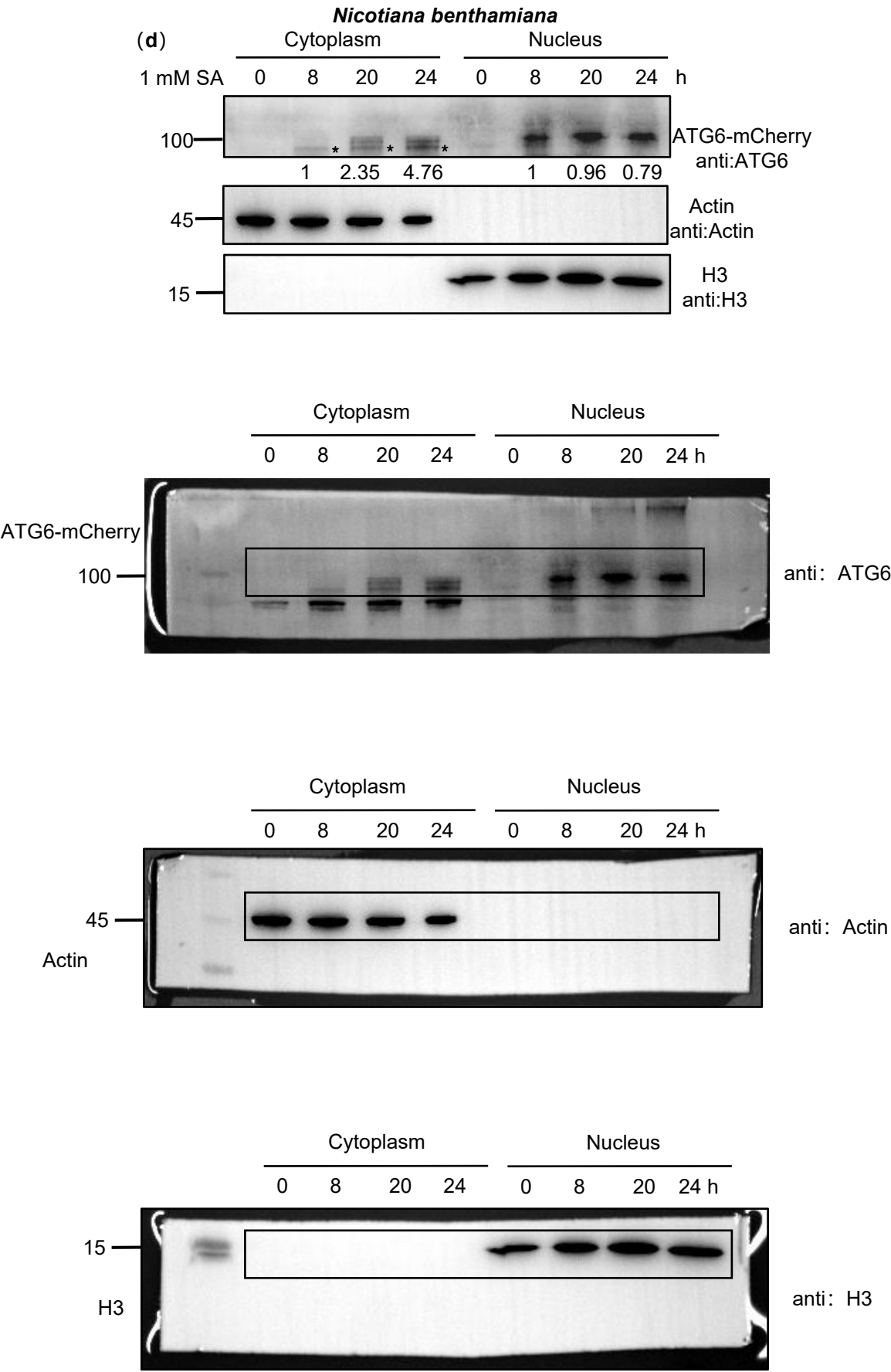

Figure 2. ATG6 is localized in the cytoplasm and nucleus

Supplement: Figure 2—source data 2. [file elife-97206-fig2-data2.zip › Figure 2-source data 2/Figure 2 d.pdf]

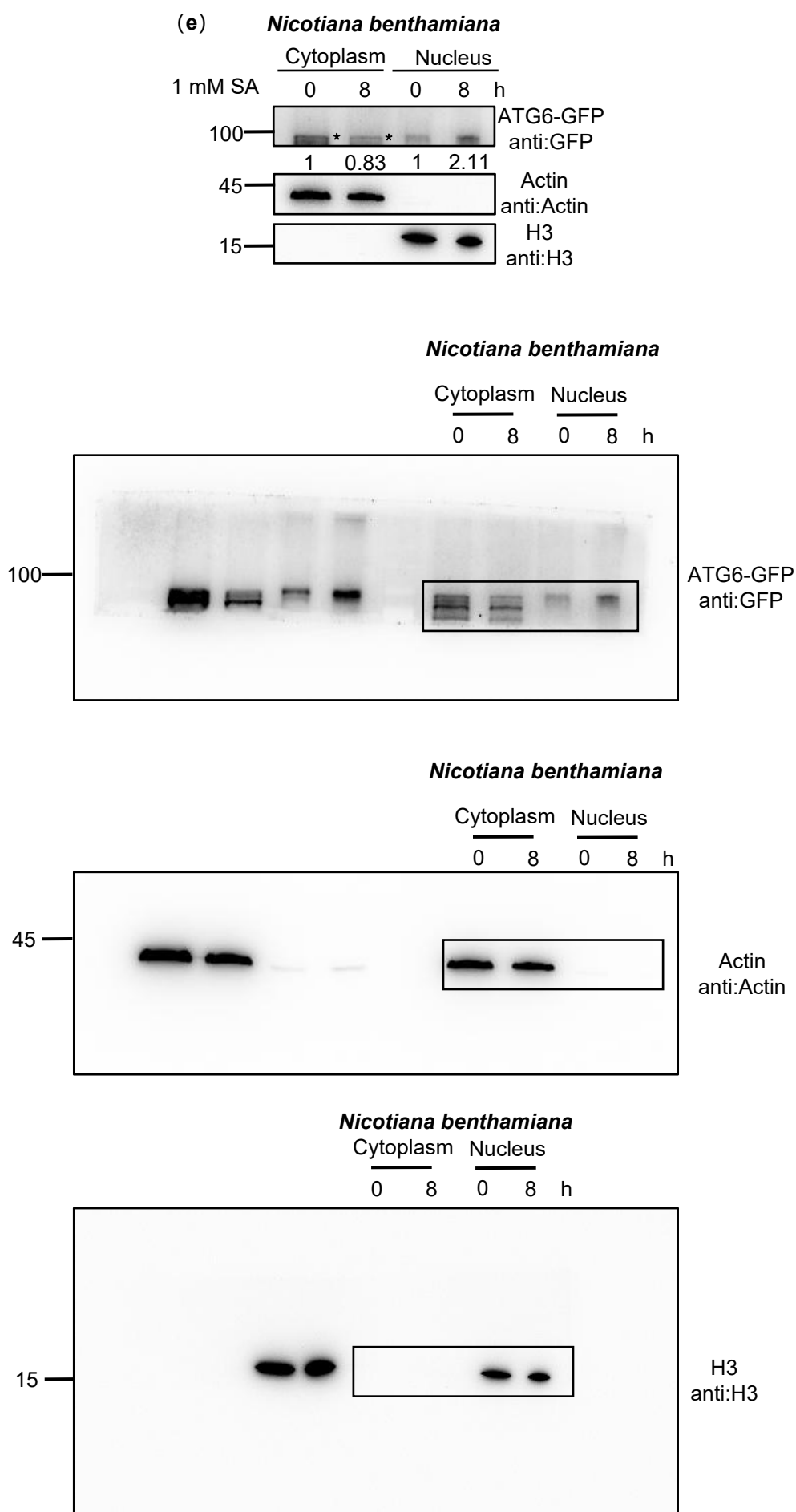

Figure 2. ATG6 is localized in the cytoplasm and nucleus

Supplement: Figure 2—source data 2. [file elife-97206-fig2-data2.zip › Figure 2-source data 2/Figure 2 e.pdf]

(f)

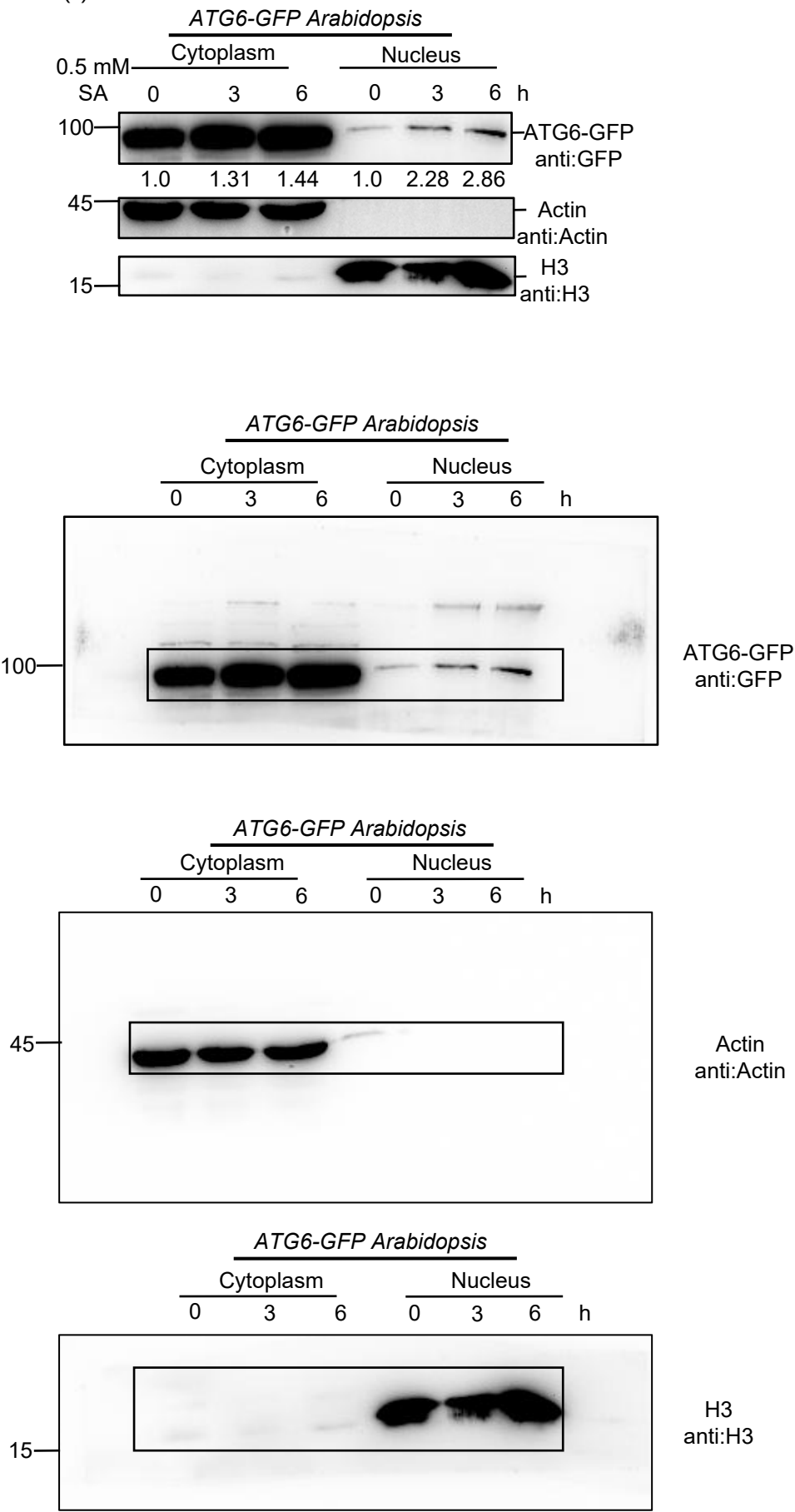

Figure 2. ATG6 is localized in the cytoplasm and nucleus

Supplement: Figure 2—source data 2. [file elife-97206-fig2-data2.zip › Figure 2-source data 2/Figure 2 f.pdf]

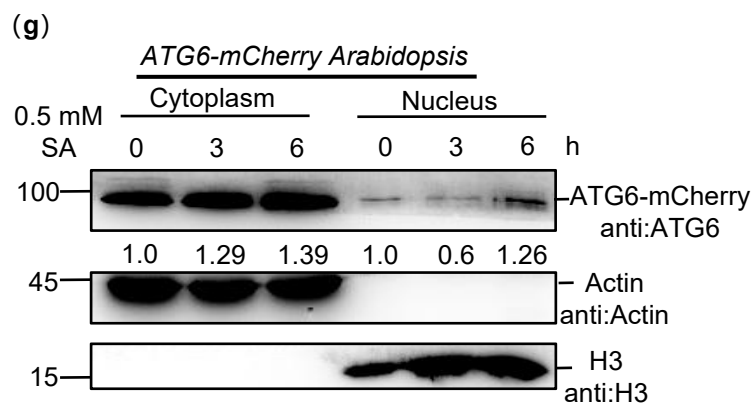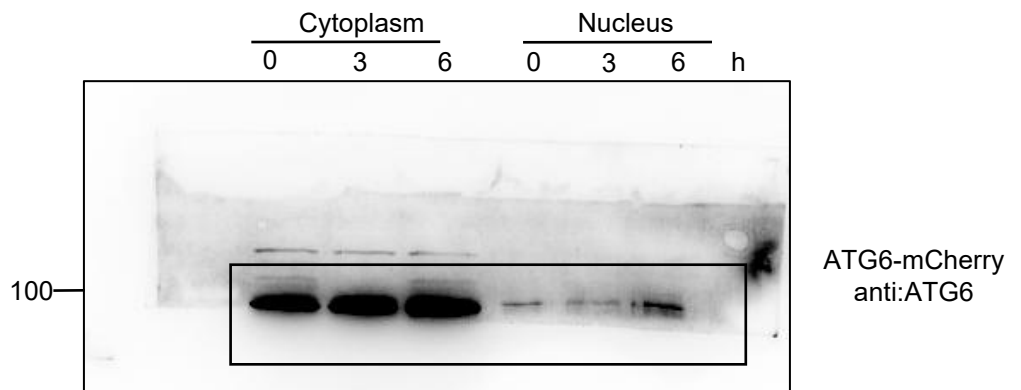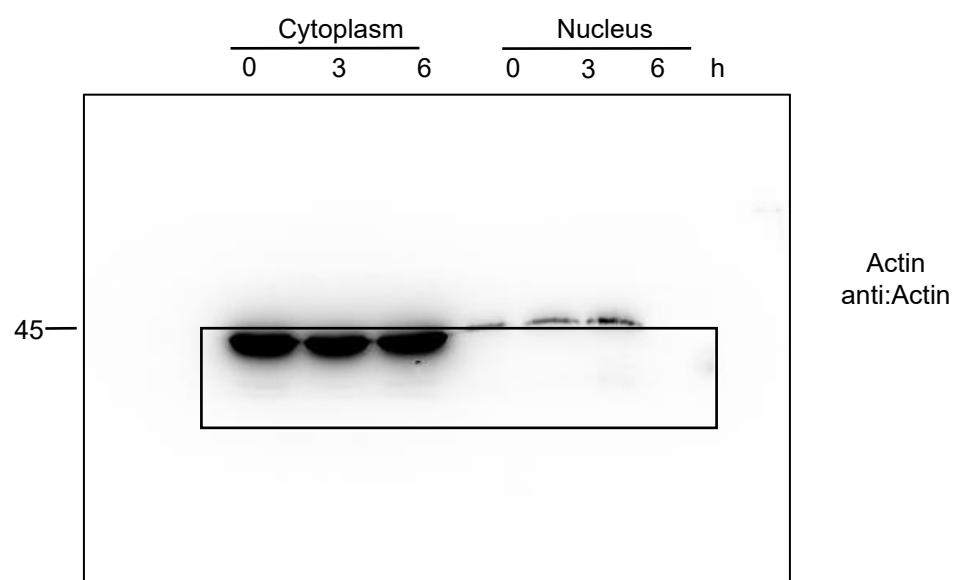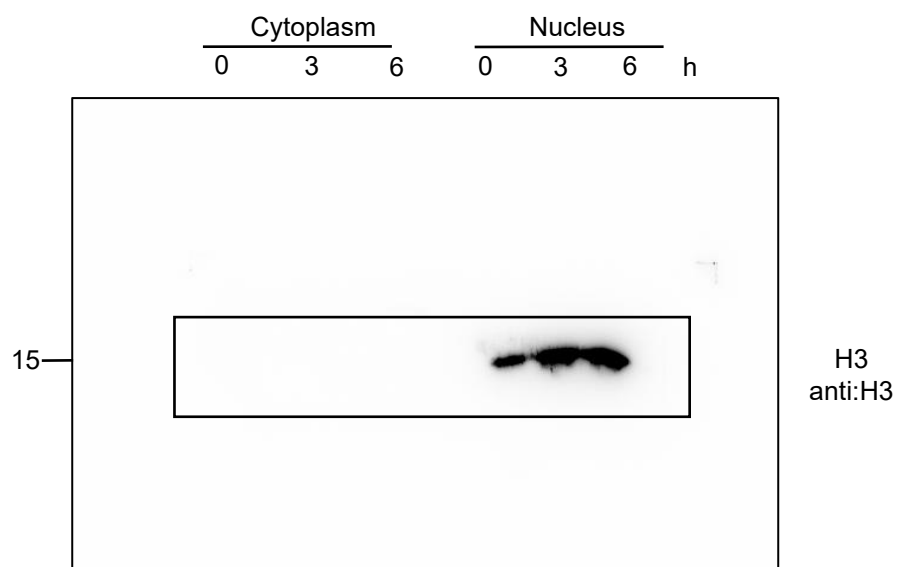

**Figure 2. ATG6 is localized in the cytoplasm and nucleus**

Supplement: Figure 2—source data 2. [file elife-97206-fig2-data2.zip › Figure 2-source data 2/Figure 2 g.pdf]

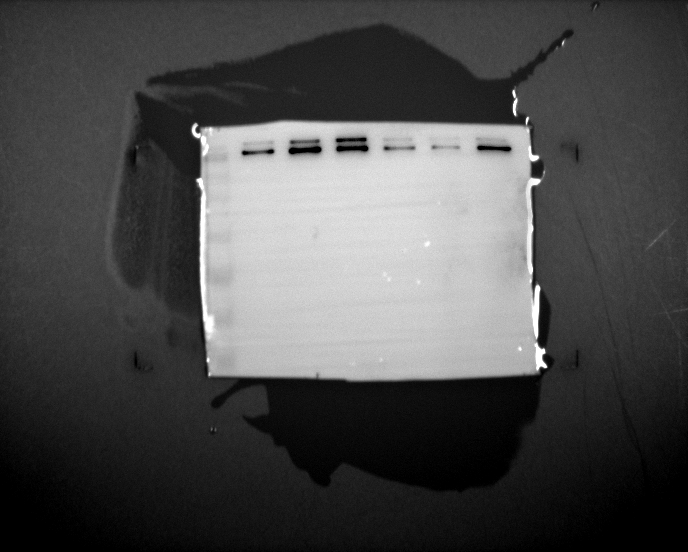

Supplement: Figure 2—figure supplement 1—source data 1. [file elife-97206-fig2-figsupp1-data1.zip › Figure 2-figure supplement 1-source data 1/Figure 2-figure supplement 1d/Original file for the Western blot analysis in figure supplement 1d (anti-ATG6-GFP).tif]

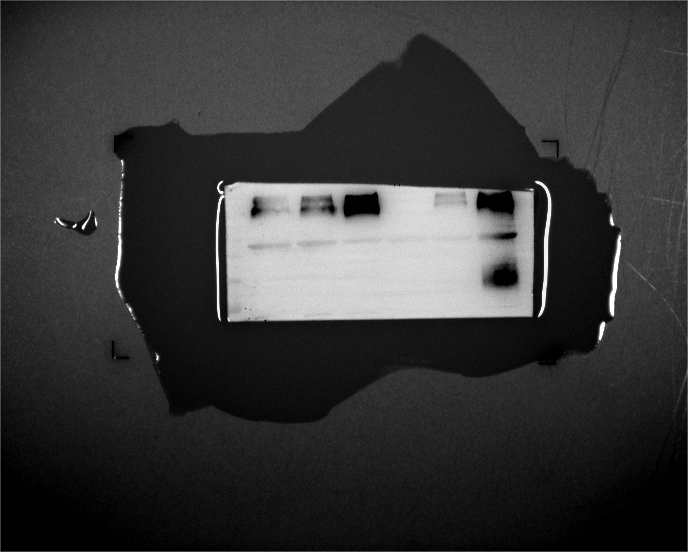

Supplement: Figure 2—figure supplement 1—source data 1. [file elife-97206-fig2-figsupp1-data1.zip › Figure 2-figure supplement 1-source data 1/Figure 2-figure supplement 1e/Original file for the Western blot analysis in figure supplement 1e (anti-ATG6-GFP).png]

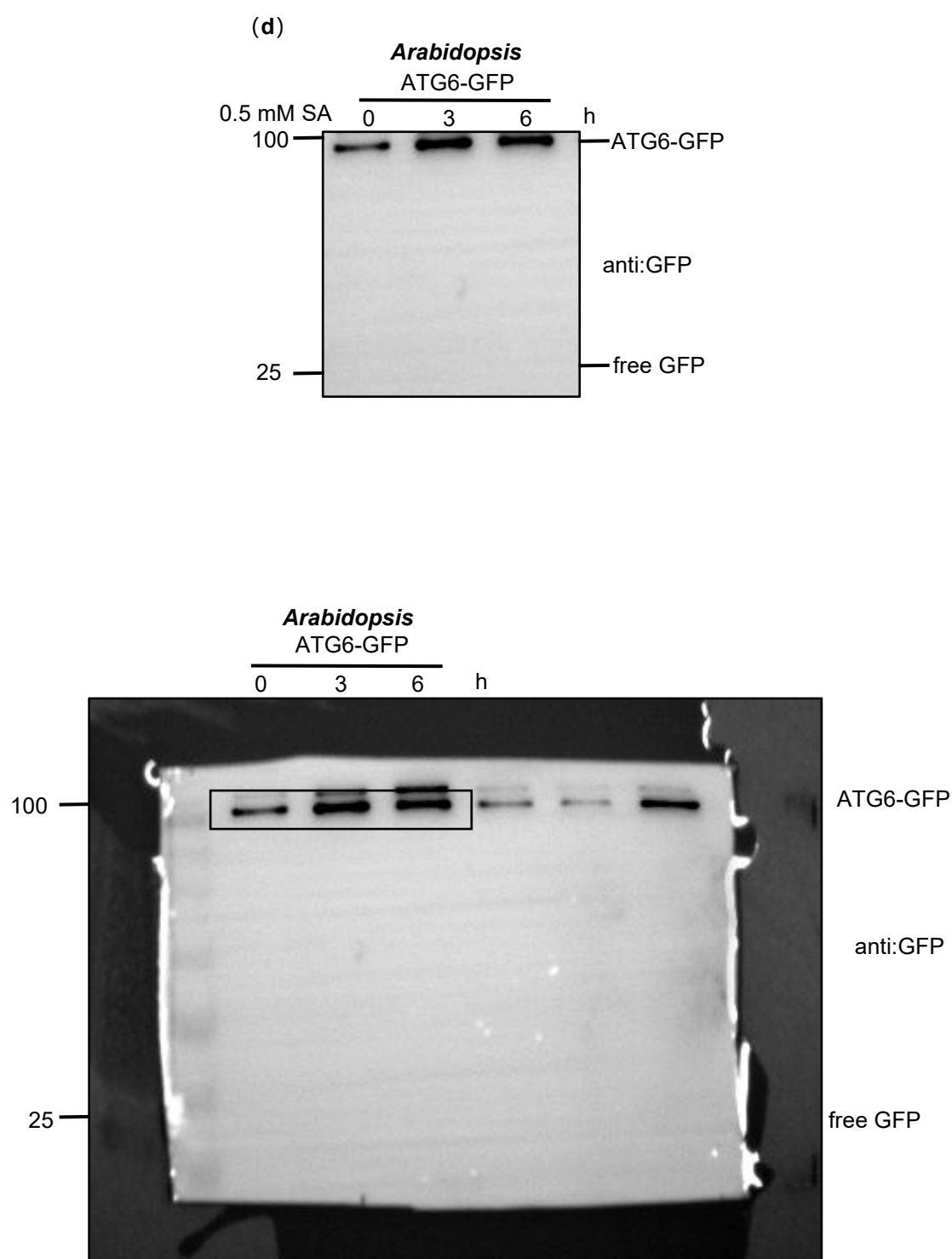

Figure 2-figure supplement 1-source data 2 The nuclear localization of ATG6 in *Arabidopsis*.

Supplement: Figure 2—figure supplement 1—source data 2. [file elife-97206-fig2-figsupp1-data2.zip › Figure 2-figure supplement 1-source data 2/Figure 2-figure supplement 1d.pdf]

(e)

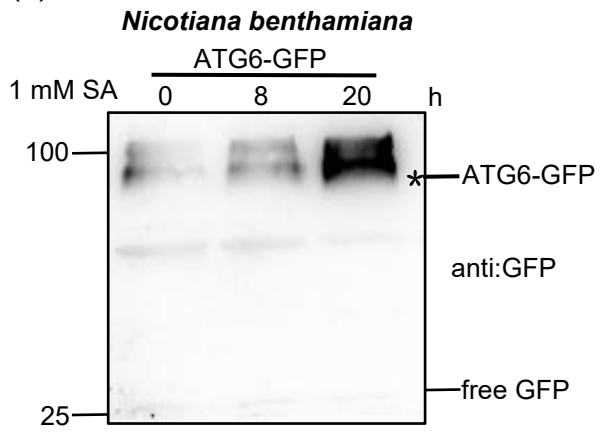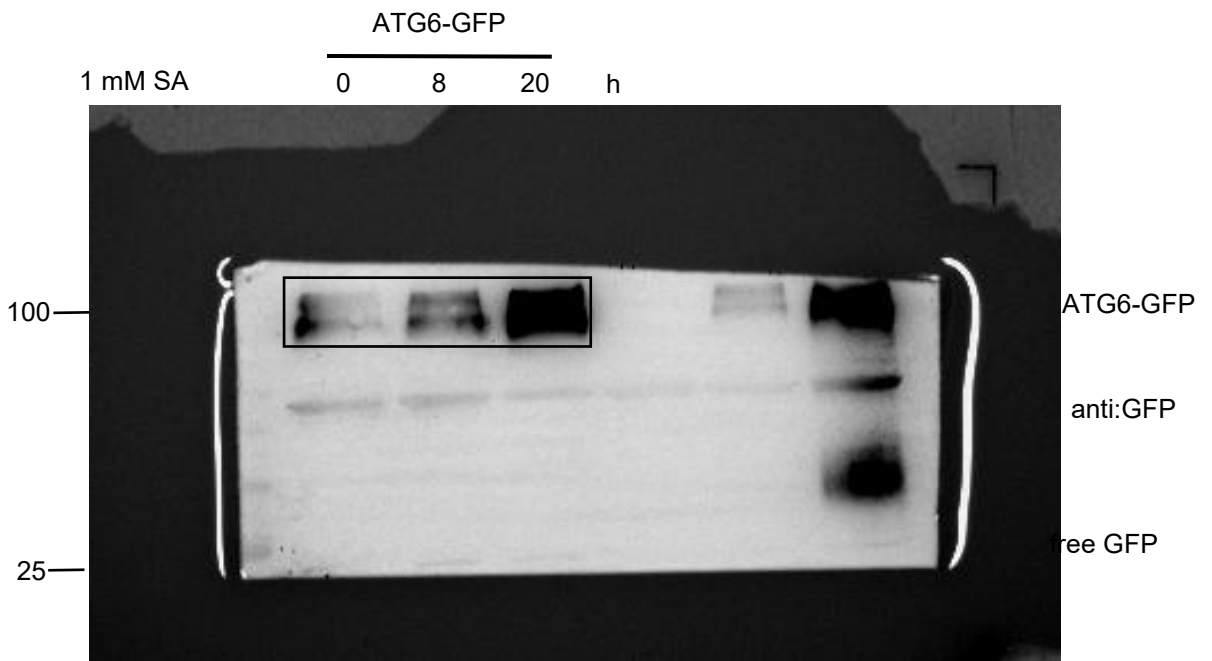

Figure 2-figure supplement 1 e The nuclear localization of ATG6 in *Arabidopsis*.

Supplement: Figure 2—figure supplement 1—source data 2. [file elife-97206-fig2-figsupp1-data2.zip › Figure 2-figure supplement 1-source data 2/Figure 2-figure supplement 1e.pdf]

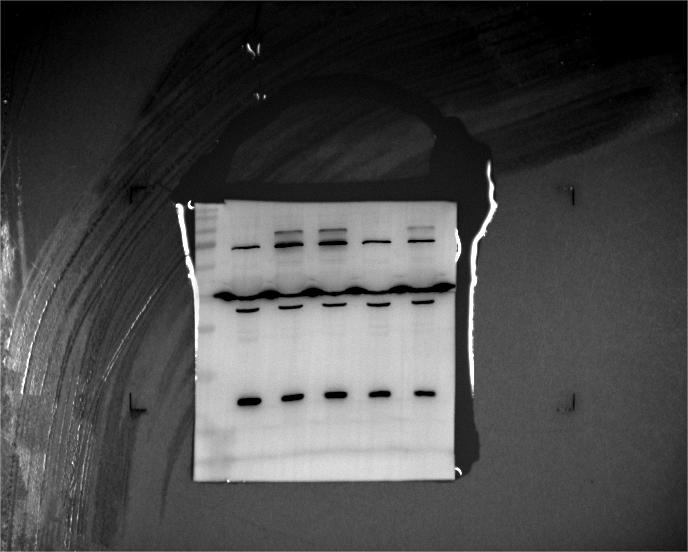

Supplement: Figure 2—figure supplement 2—source data 1. [file elife-97206-fig2-figsupp2-data1.zip › Figure 2-figure supplement 2-source data 1/Figure 2-figure supplement 2a/Original file for the Western blot in figure supplement 2a (anti-ATG6-mCherry).tif]

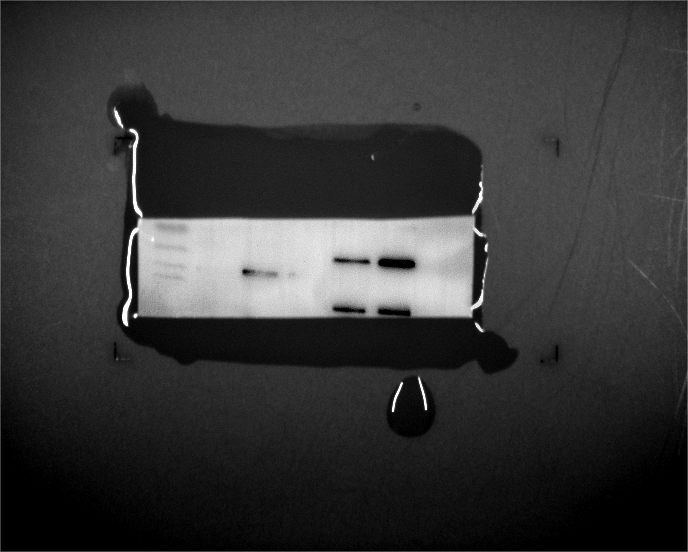

Supplement: Figure 2—figure supplement 2—source data 1. [file elife-97206-fig2-figsupp2-data1.zip › Figure 2-figure supplement 2-source data 1/Figure 2-figure supplement 2a/Original file for the Western blot in figure supplement 2a (anti-NPR1-GFP).tif]

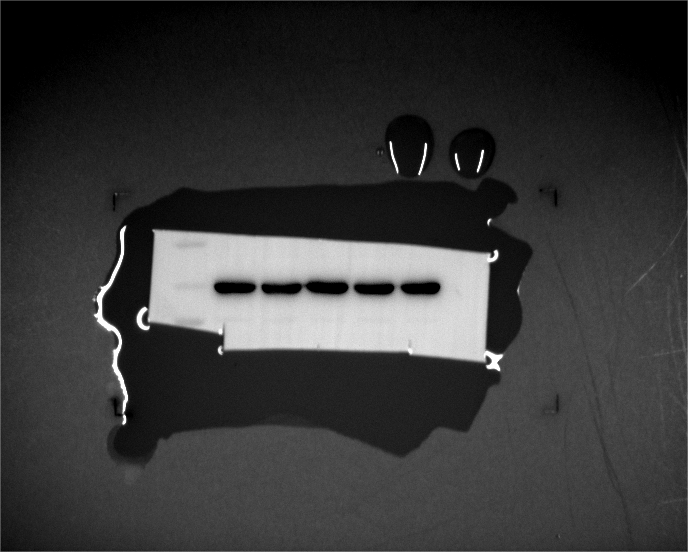

Supplement: Figure 2—figure supplement 2—source data 1. [file elife-97206-fig2-figsupp2-data1.zip › Figure 2-figure supplement 2-source data 1/Figure 2-figure supplement 2a/Original file for the Western blot analysis in figure supplement 2a (anti-Actin).tif]

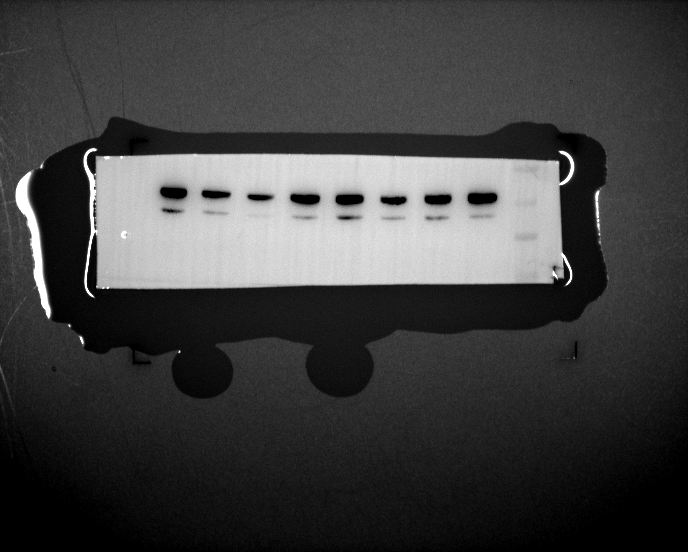

Supplement: Figure 2—figure supplement 2—source data 1. [file elife-97206-fig2-figsupp2-data1.zip › Figure 2-figure supplement 2-source data 1/Figure 2-figure supplement 2b/Original file for the Western blot analysis in figure supplement 2b (anti-Actin).tif]

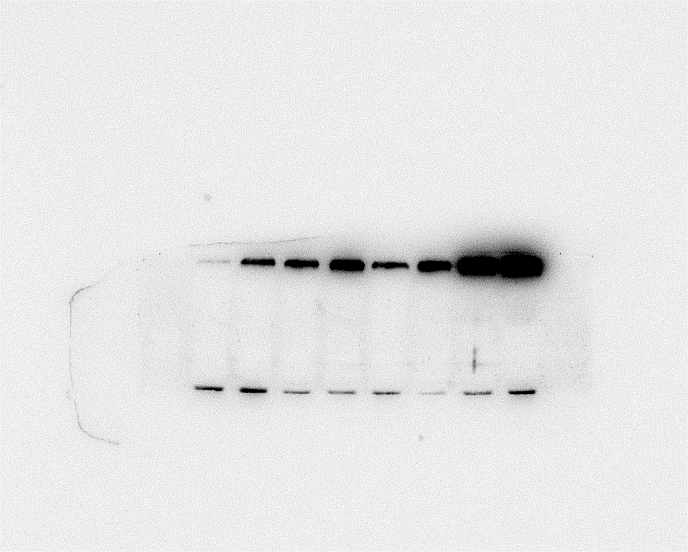

Supplement: Figure 2—figure supplement 2—source data 1. [file elife-97206-fig2-figsupp2-data1.zip › Figure 2-figure supplement 2-source data 1/Figure 2-figure supplement 2b/Original file for the Western blot analysis in figure supplement 2b (anti-NPR1-GFP).tif]

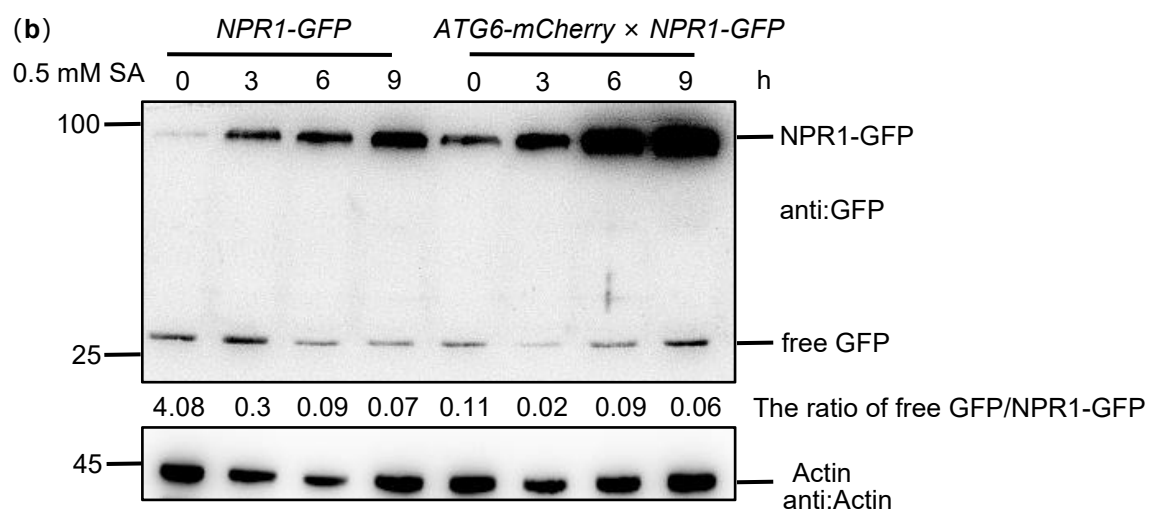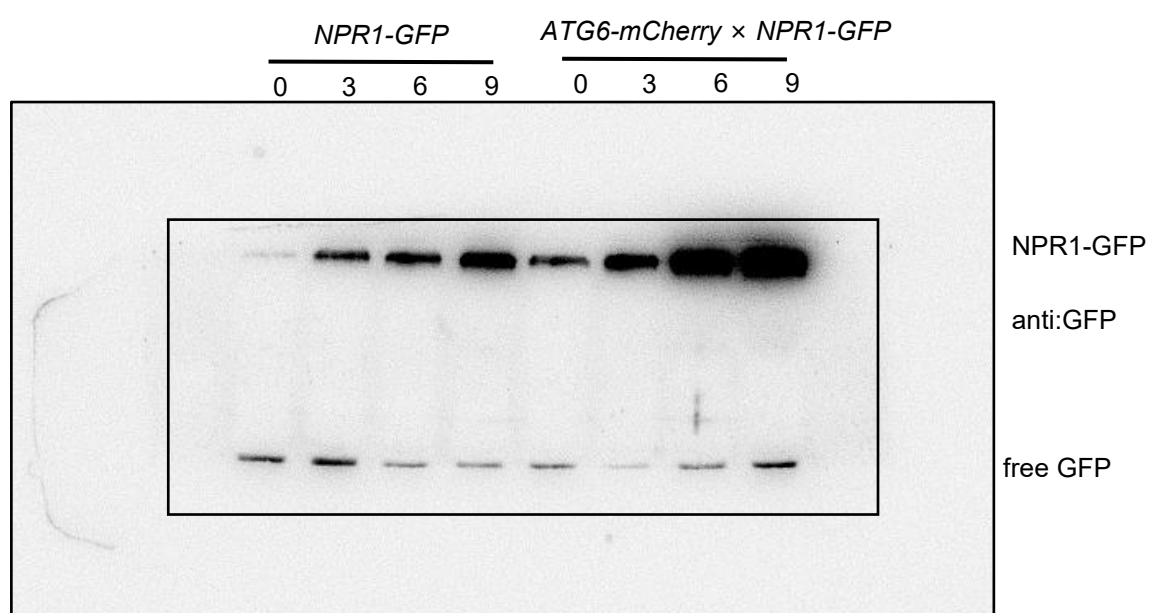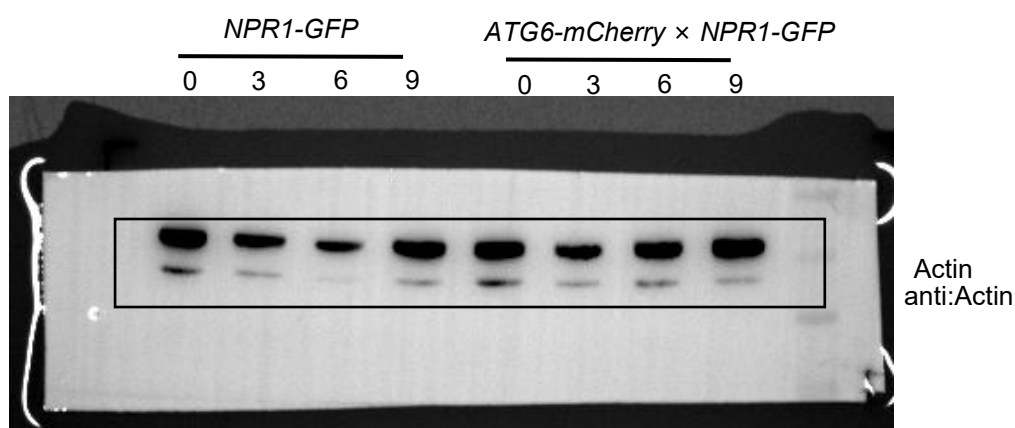

Figure 2-figure supplement 2 Identification of *ATG6-mCherry × NPR1-GFP* plants.

Supplement: Figure 2—figure supplement 2—source data 2. [file elife-97206-fig2-figsupp2-data2.zip › Figure 2-figure supplement 2-source data 2/Figure 2-figure supplement 2b.pdf]

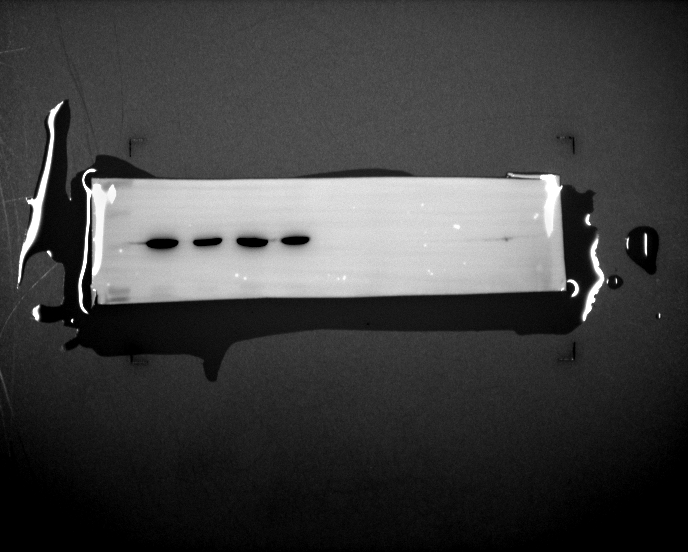

Supplement: Figure 2—figure supplement 3—source data 1. [file elife-97206-fig2-figsupp3-data1.zip › Figure 2-figure supplement 3-source data 1/Original file for the Western blot analysis in Figure 2-figure supplement 3 (anti-Actin).tif]

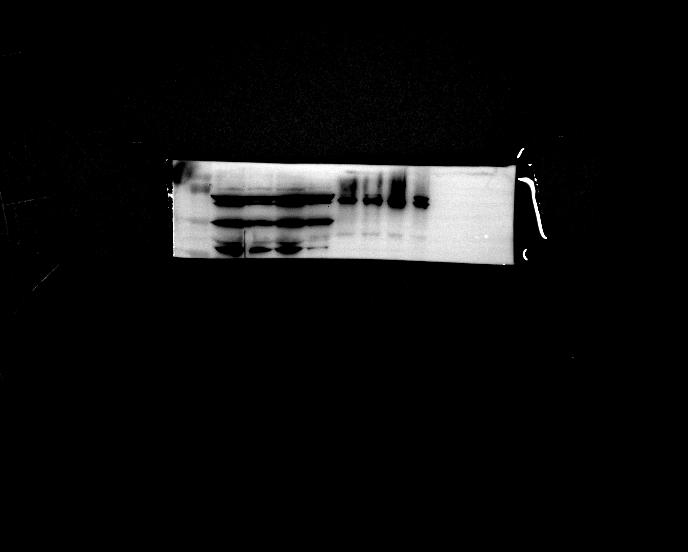

Supplement: Figure 2—figure supplement 3—source data 1. [file elife-97206-fig2-figsupp3-data1.zip › Figure 2-figure supplement 3-source data 1/Original file for the Western blot analysis in Figure 2-figure supplement 3 (anti-ATG6).tif]

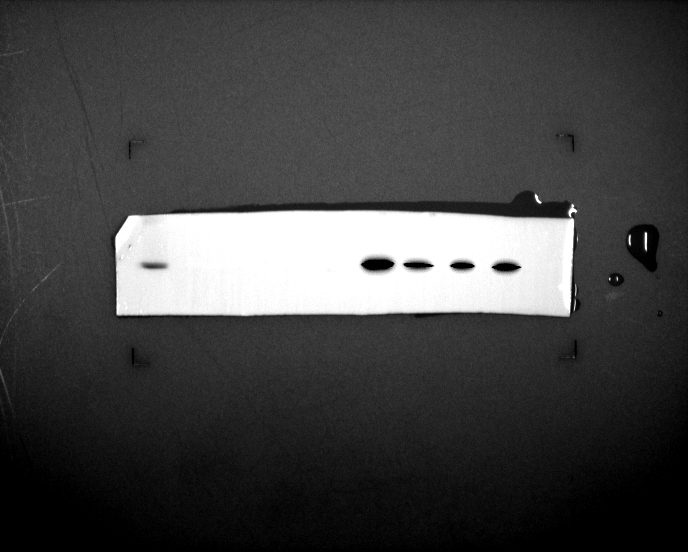

Supplement: Figure 2—figure supplement 3—source data 1. [file elife-97206-fig2-figsupp3-data1.zip › Figure 2-figure supplement 3-source data 1/Original file for the Western blot analysis in Figure 2-figure supplement 3 (anti-H3).tif]

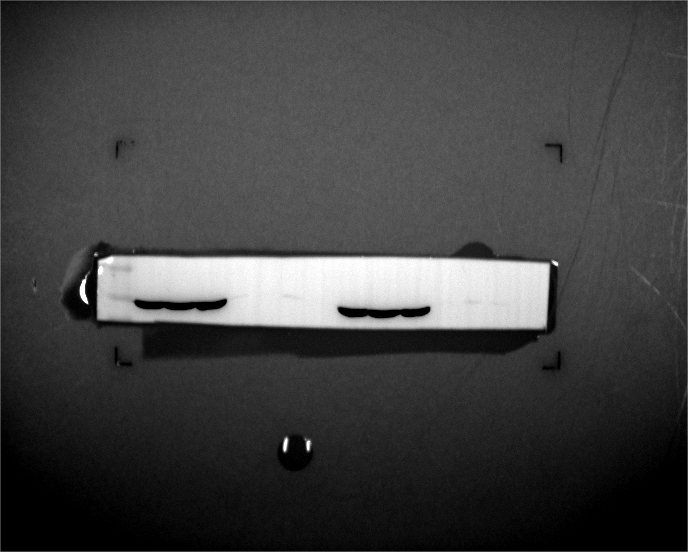

Supplement: Figure 3—source data 1. [file elife-97206-fig3-data1.zip › Figure 3-source data 1/Figure 3 c/Original file for the Western blot analysis in Figure 3c (anti-Actin) .tif]

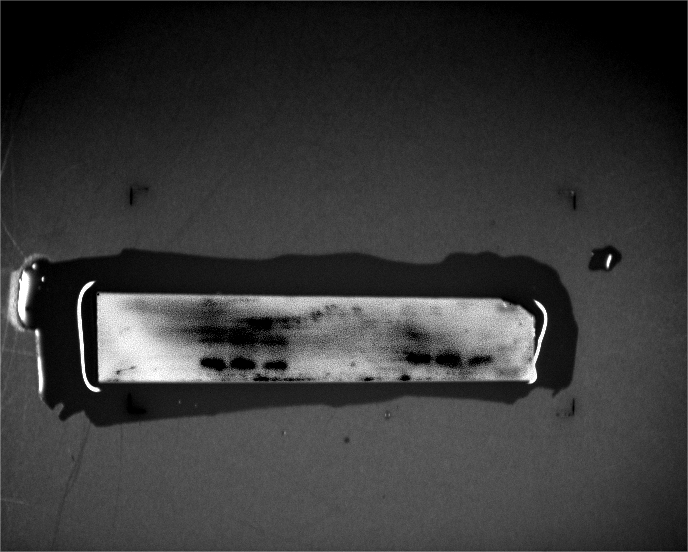

Supplement: Figure 3—source data 1. [file elife-97206-fig3-data1.zip › Figure 3-source data 1/Figure 3 c/Original file for the Western blot analysis in Figure 3c (anti-H3) .tif]

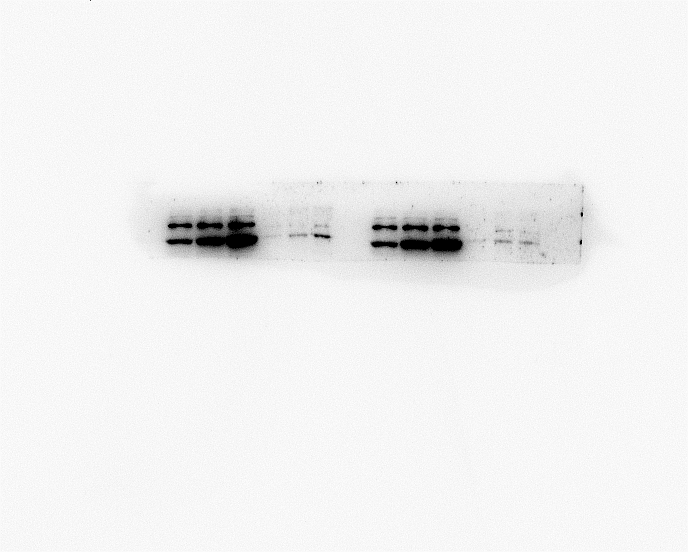

Supplement: Figure 3—source data 1. [file elife-97206-fig3-data1.zip › Figure 3-source data 1/Figure 3 c/Original file for the Western blot analysis in Figure 3c (anti-NPR1-GFP) .tif]

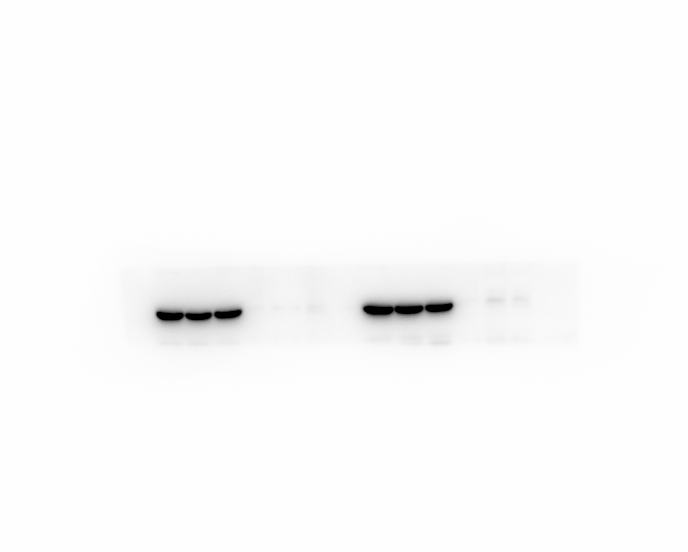

Supplement: Figure 3—source data 1. [file elife-97206-fig3-data1.zip › Figure 3-source data 1/Figure 3 e/Original file for the Western blot analysis in Figure 3e (anti-Actin) .tif]

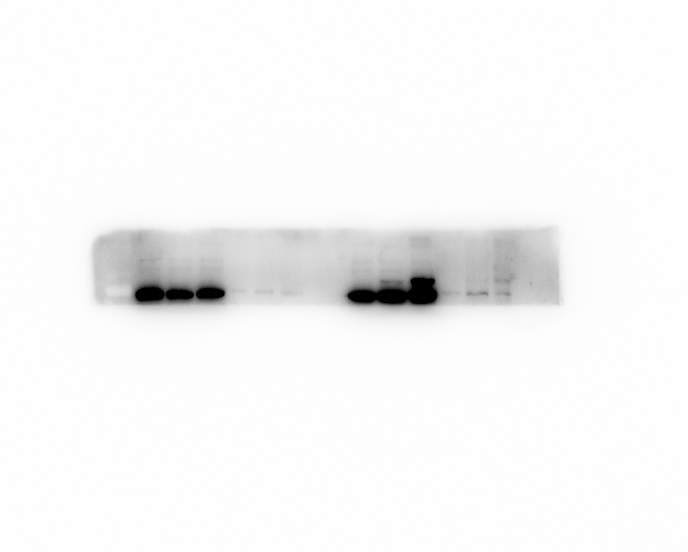

Supplement: Figure 3—source data 1. [file elife-97206-fig3-data1.zip › Figure 3-source data 1/Figure 3 e/Original file for the Western blot analysis in Figure 3e (anti-ATG6-mCherry) .tif]

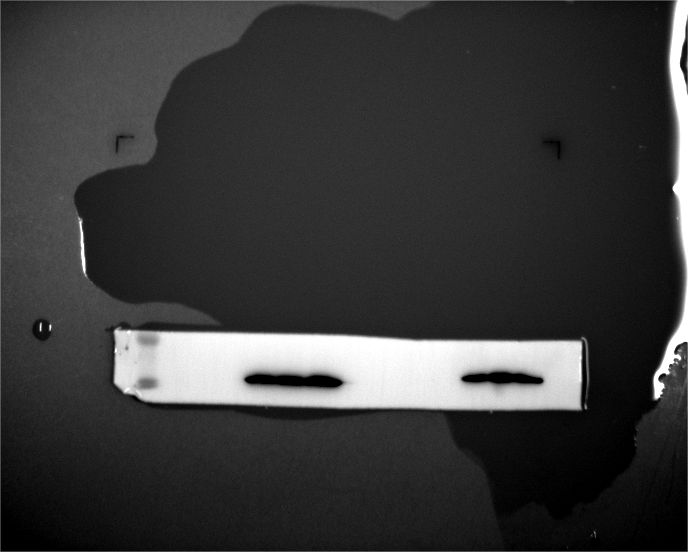

Supplement: Figure 3—source data 1. [file elife-97206-fig3-data1.zip › Figure 3-source data 1/Figure 3 e/Original file for the Western blot analysis in Figure 3e (anti-H3) .tif]

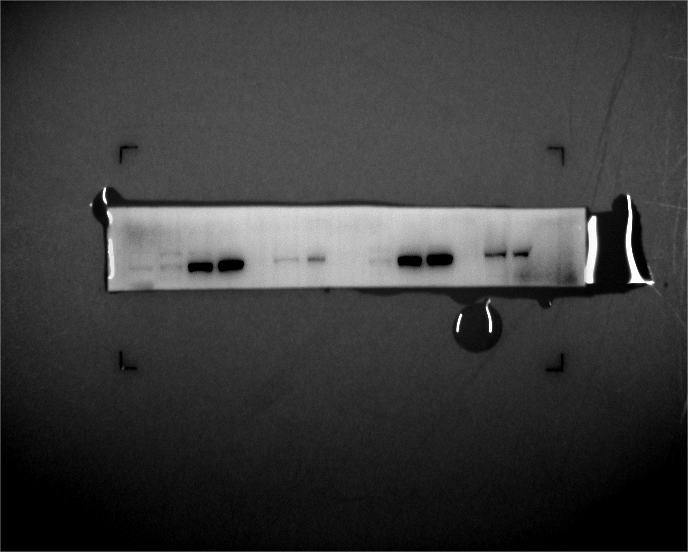

Supplement: Figure 3—source data 1. [file elife-97206-fig3-data1.zip › Figure 3-source data 1/Figure 3 e/Original file for the Western blot analysis in Figure 3e (anti-NPR1-GFP) .tif]

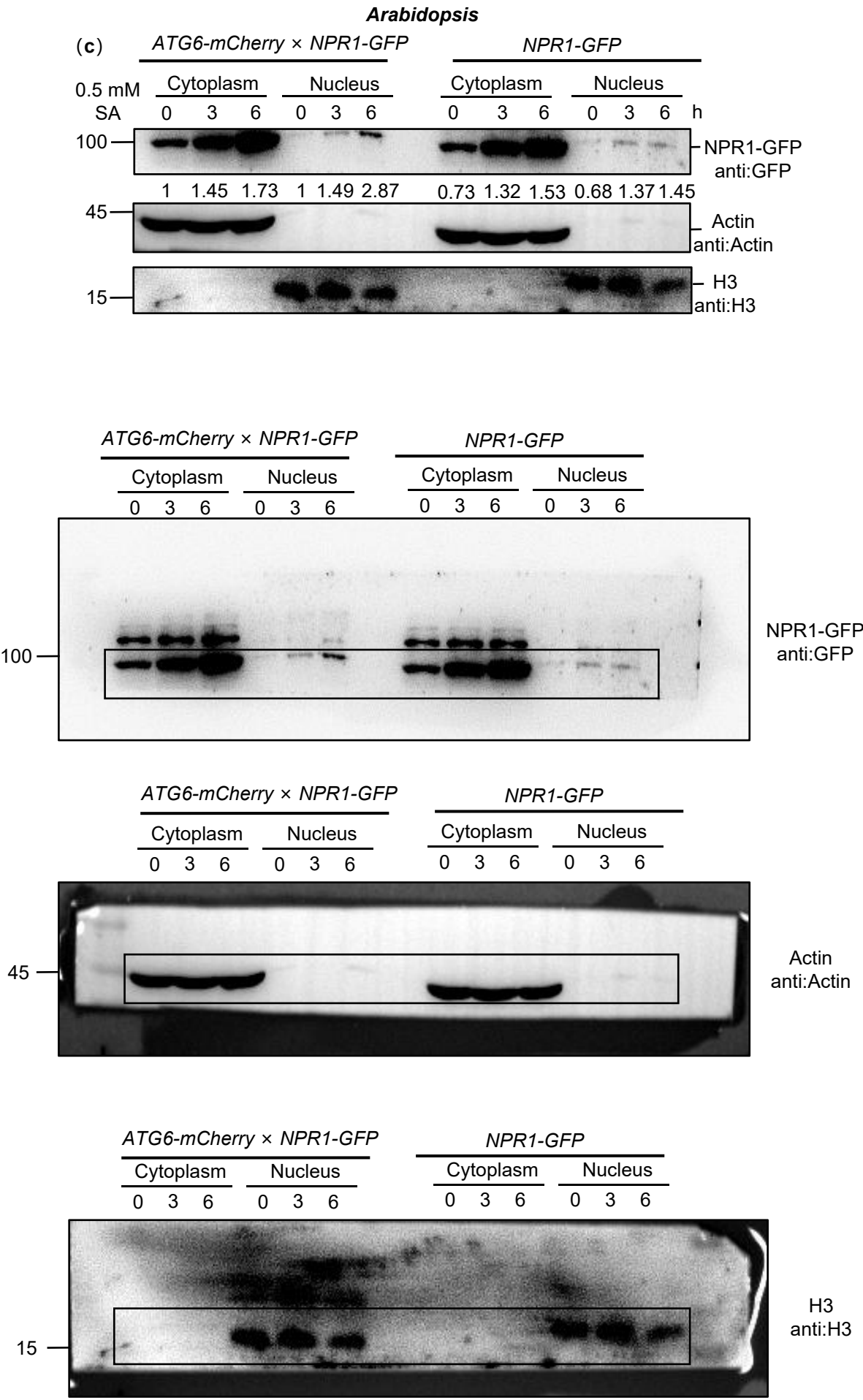

Figure 3. ATG6 increases the nuclear accumulation of NPR1 under SA treatment.

Supplement: Figure 3—source data 2. [file elife-97206-fig3-data2.zip › Figure 3-source data 2/Figure 3 c.pdf]

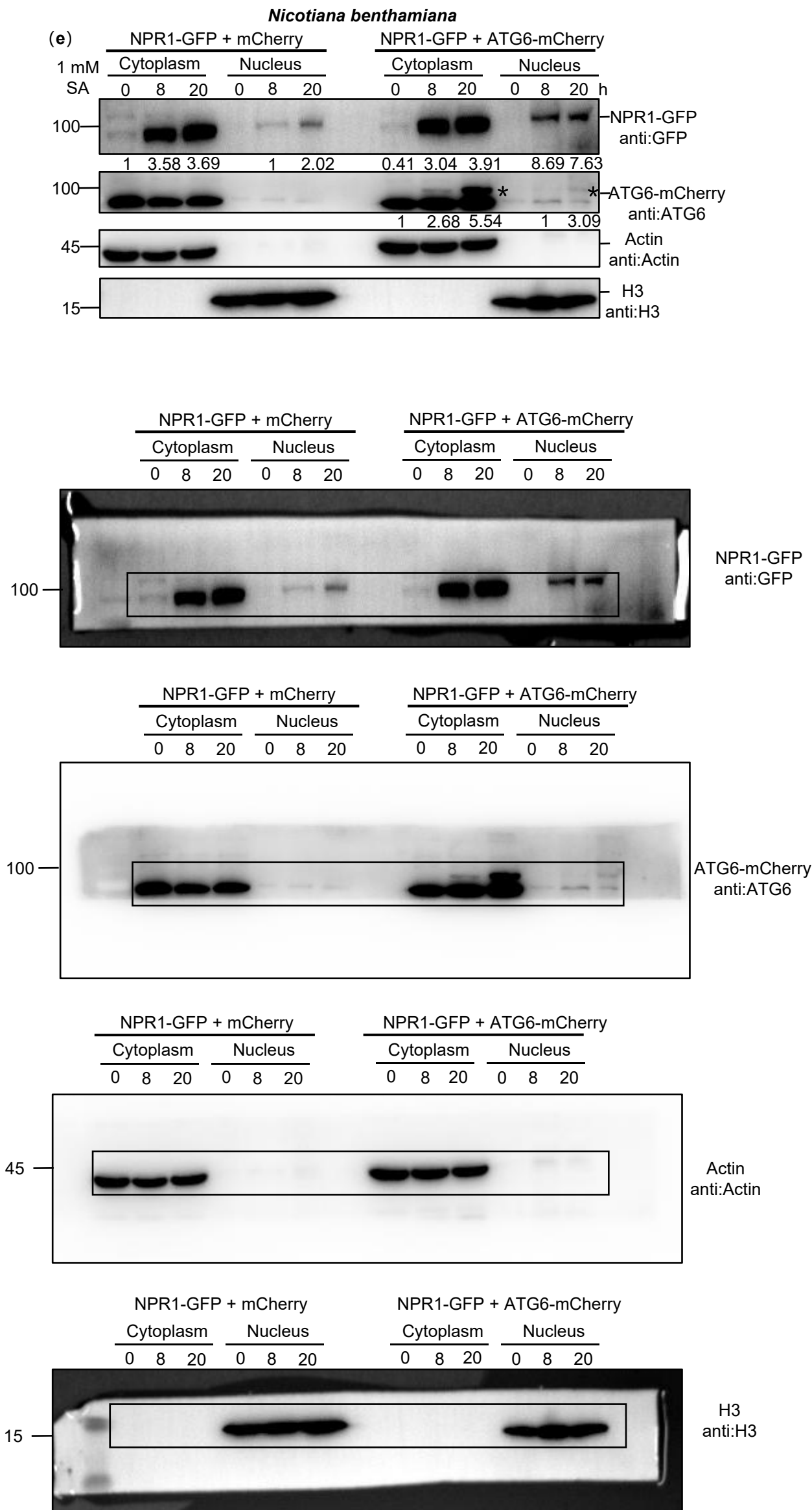

**Figure 3. ATG6 increases the nuclear accumulation of NPR1 under SA treatment.**

Supplement: Figure 3—source data 2. [file elife-97206-fig3-data2.zip › Figure 3-source data 2/Figure 3 e.pdf]

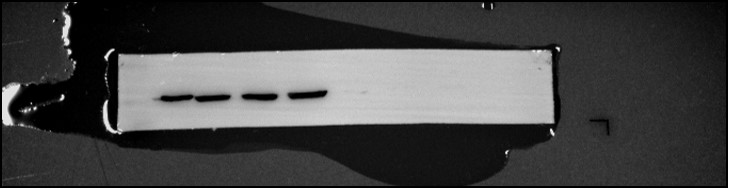

Supplement: Figure 3—figure supplement 2—source data 1. [file elife-97206-fig3-figsupp2-data1.zip › Figure 3-figure supplement 2-source data 1/Figure 3-figure supplement 2 repeat 1/Original file for the Western blot in repeat 1 anti-Actin left.jpg]

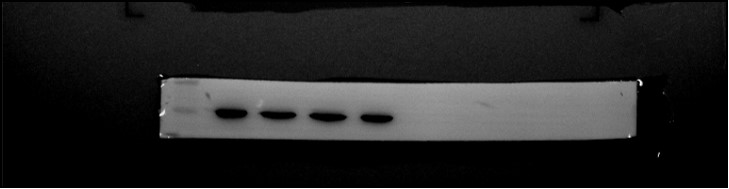

Supplement: Figure 3—figure supplement 2—source data 1. [file elife-97206-fig3-figsupp2-data1.zip › Figure 3-figure supplement 2-source data 1/Figure 3-figure supplement 2 repeat 1/Original file for the Western blot in repeat 1 anti-Actin right.jpg]

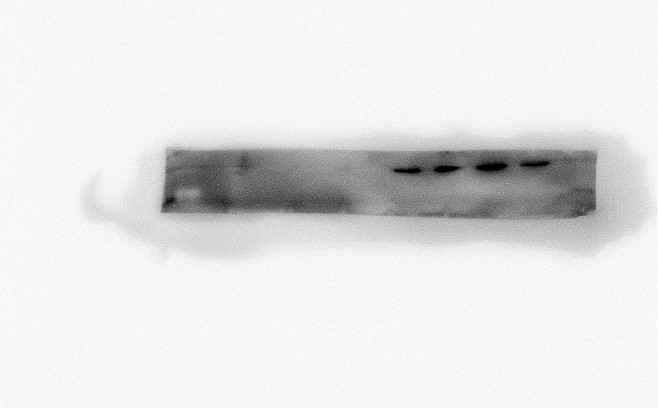

Supplement: Figure 3—figure supplement 2—source data 1. [file elife-97206-fig3-figsupp2-data1.zip › Figure 3-figure supplement 2-source data 1/Figure 3-figure supplement 2 repeat 1/Original file for the Western blot in repeat 1 anti-H3 left.jpg]

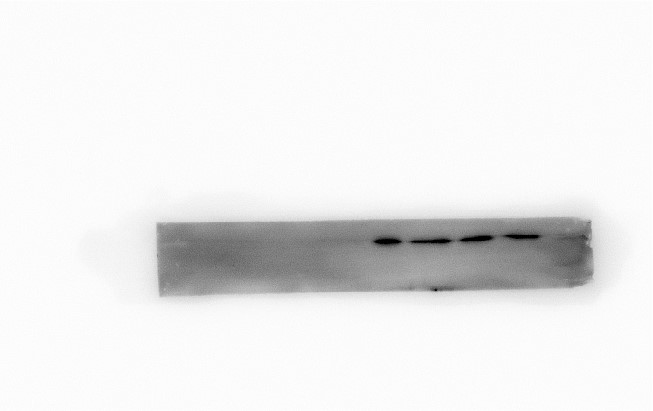

Supplement: Figure 3—figure supplement 2—source data 1. [file elife-97206-fig3-figsupp2-data1.zip › Figure 3-figure supplement 2-source data 1/Figure 3-figure supplement 2 repeat 1/Original file for the Western blot in repeat 1 anti-H3 right.jpg]

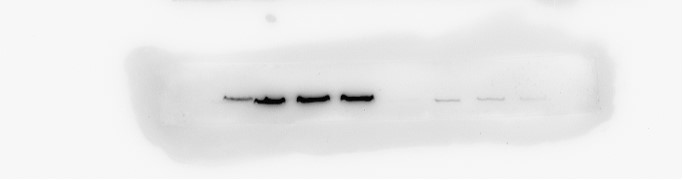

Supplement: Figure 3—figure supplement 2—source data 1. [file elife-97206-fig3-figsupp2-data1.zip › Figure 3-figure supplement 2-source data 1/Figure 3-figure supplement 2 repeat 1/Original file for the Western blot in repeat 1 anti-NPR1-GFP left.jpg]

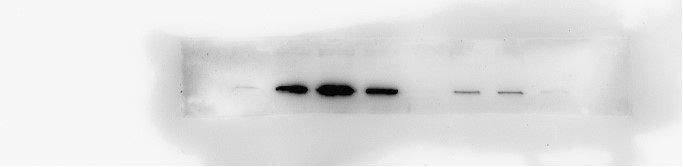

Supplement: Figure 3—figure supplement 2—source data 1. [file elife-97206-fig3-figsupp2-data1.zip › Figure 3-figure supplement 2-source data 1/Figure 3-figure supplement 2 repeat 1/Original file for the Western blot in repeat 1 anti-NPR1-GFP right.jpg]

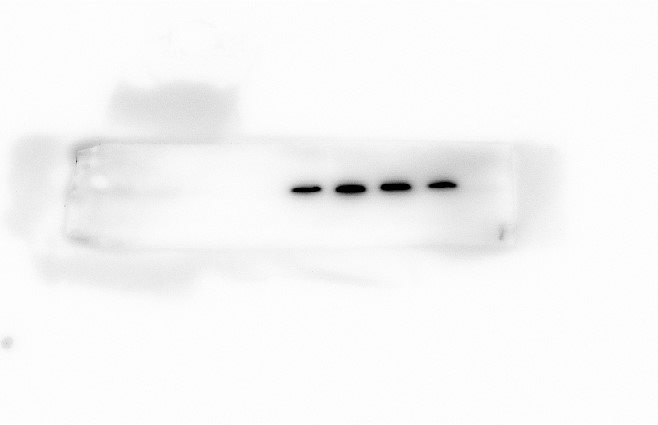

Supplement: Figure 3—figure supplement 2—source data 1. [file elife-97206-fig3-figsupp2-data1.zip › Figure 3-figure supplement 2-source data 1/Figure 3-figure supplement 2 repeat 2/Original file for the Western blot in repeat 2 anti-H3 left.jpg]

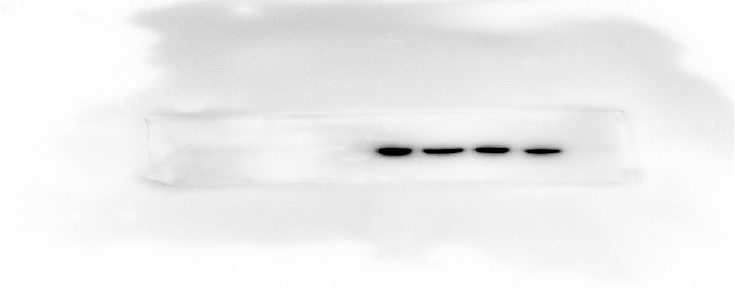

Supplement: Figure 3—figure supplement 2—source data 1. [file elife-97206-fig3-figsupp2-data1.zip › Figure 3-figure supplement 2-source data 1/Figure 3-figure supplement 2 repeat 2/Original file for the Western blot in repeat 2 anti-H3 right.jpg]

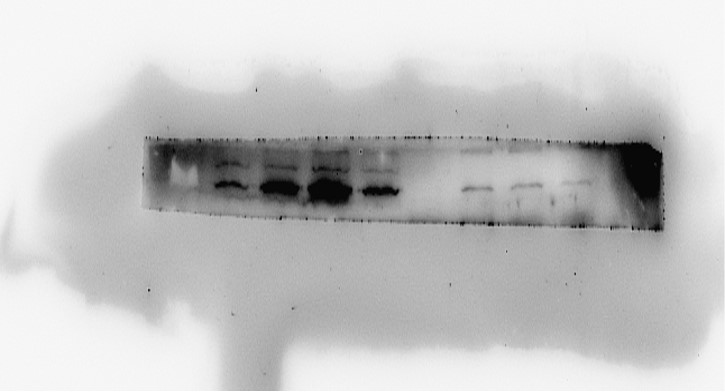

Supplement: Figure 3—figure supplement 2—source data 1. [file elife-97206-fig3-figsupp2-data1.zip › Figure 3-figure supplement 2-source data 1/Figure 3-figure supplement 2 repeat 2/Original file for the Western blot in repeat 2 anti-NPR1-GFP left.jpg]

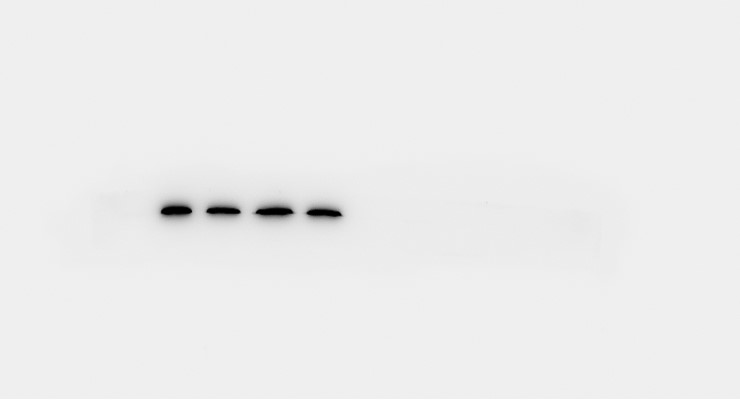

Supplement: Figure 3—figure supplement 2—source data 1. [file elife-97206-fig3-figsupp2-data1.zip › Figure 3-figure supplement 2-source data 1/Figure 3-figure supplement 2 repeat 2/Original file for the Western blot in repeat 2 anti-Actin left.jpg]

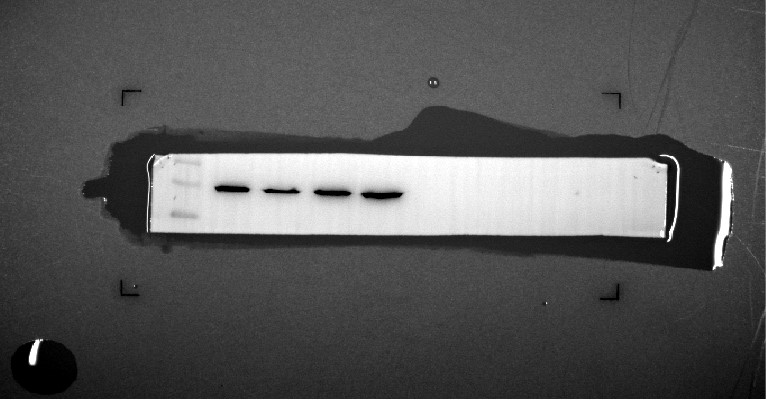

Supplement: Figure 3—figure supplement 2—source data 1. [file elife-97206-fig3-figsupp2-data1.zip › Figure 3-figure supplement 2-source data 1/Figure 3-figure supplement 2 repeat 2/Original file for the Western blot in repeat 2 anti-Actin right.jpg]

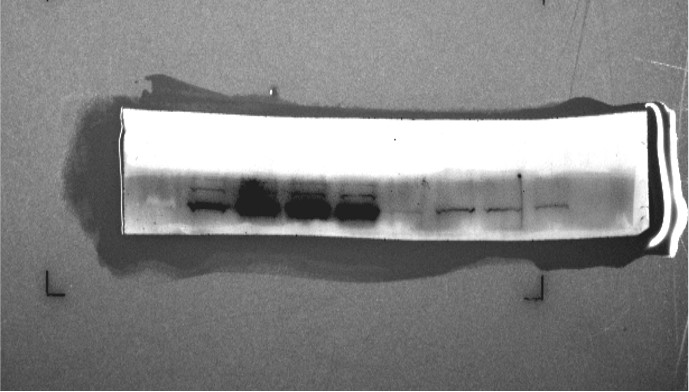

Supplement: Figure 3—figure supplement 2—source data 1. [file elife-97206-fig3-figsupp2-data1.zip › Figure 3-figure supplement 2-source data 1/Figure 3-figure supplement 2 repeat 2/Original file for the Western blot in repeat 2 anti-NPR1-GFP right.jpg]

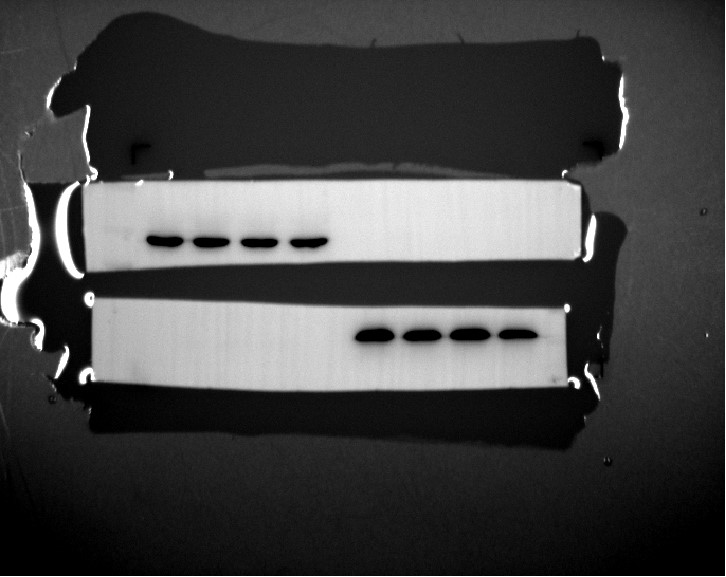

Supplement: Figure 3—figure supplement 2—source data 1. [file elife-97206-fig3-figsupp2-data1.zip › Figure 3-figure supplement 2-source data 1/Figure 3-figure supplement 2 repeat 3/Original file for the Western blot in repeat 3 anti-actin.jpg]

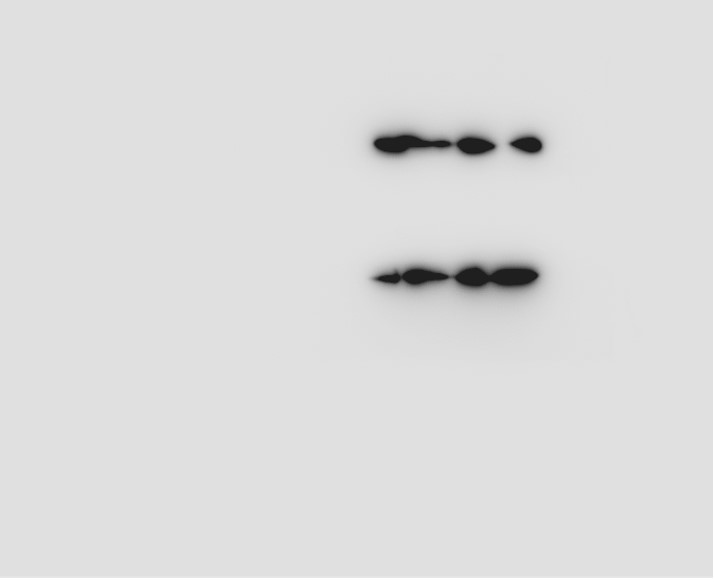

Supplement: Figure 3—figure supplement 2—source data 1. [file elife-97206-fig3-figsupp2-data1.zip › Figure 3-figure supplement 2-source data 1/Figure 3-figure supplement 2 repeat 3/Original file for the Western blot in repeat 3 anti-H3.jpg]

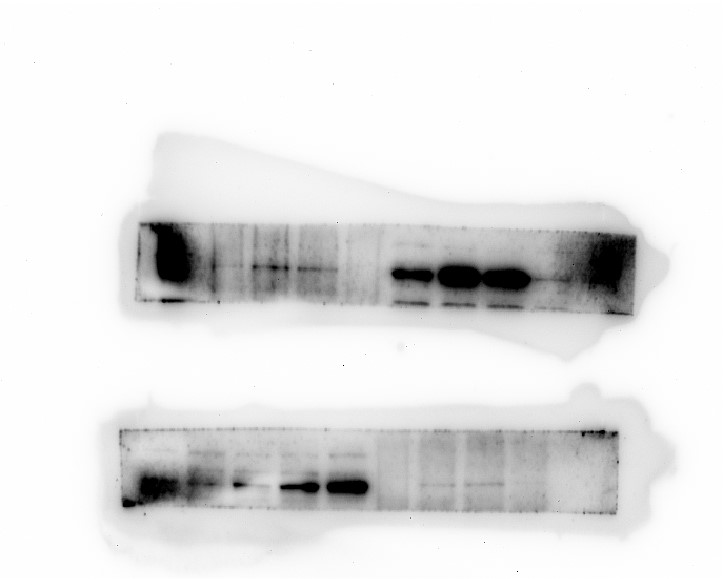

Supplement: Figure 3—figure supplement 2—source data 1. [file elife-97206-fig3-figsupp2-data1.zip › Figure 3-figure supplement 2-source data 1/Figure 3-figure supplement 2 repeat 3/Original file for the Western blot in repeat 3 anti-NPR1-GFP.jpg]

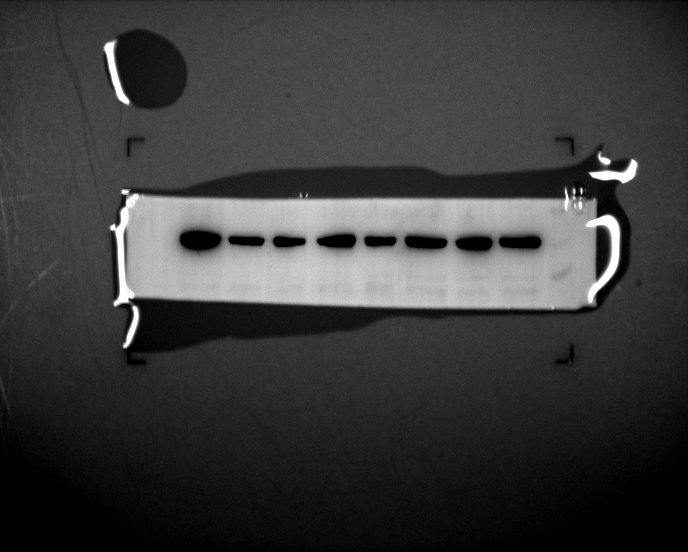

Supplement: Figure 5—source data 1. [file elife-97206-fig5-data1.zip › Figure 5-source data 1/Figure 5 a/Original file for the Western blot analysis in Figure 5a (anti-Actin) .tif]

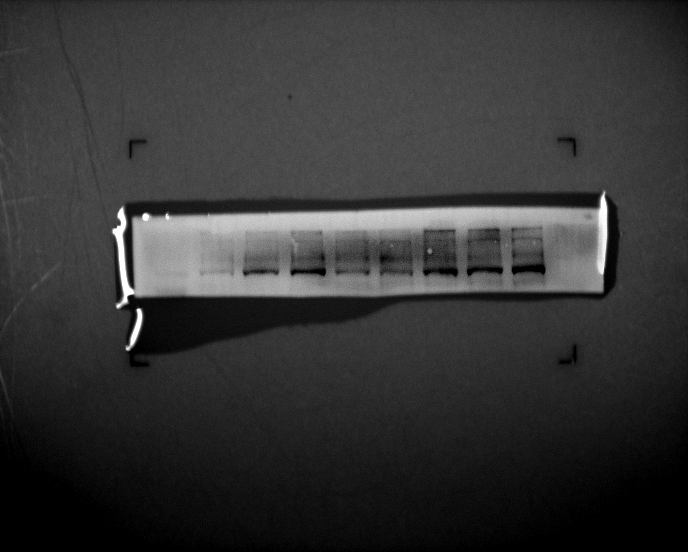

Supplement: Figure 5—source data 1. [file elife-97206-fig5-data1.zip › Figure 5-source data 1/Figure 5 a/Original file for the Western blot analysis in Figure 5a (anti-NPR1-GFP) .tif]

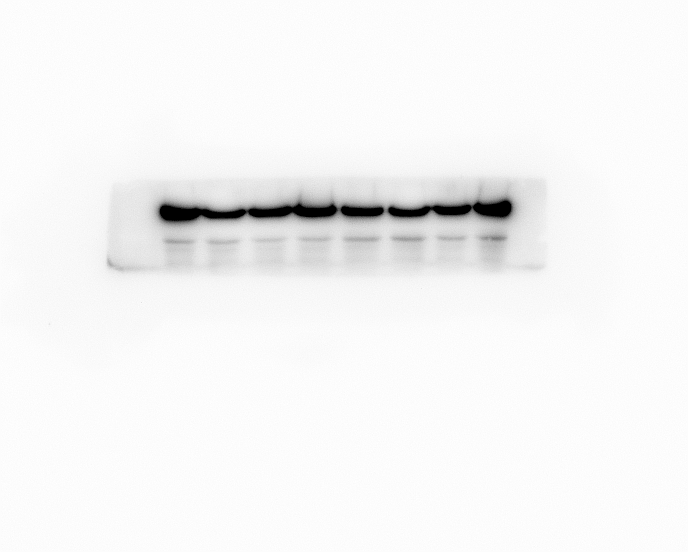

Supplement: Figure 5—source data 1. [file elife-97206-fig5-data1.zip › Figure 5-source data 1/Figure 5 b/Original file for the Western blot analysis in Figure 5b (anti-Actin) .tif]

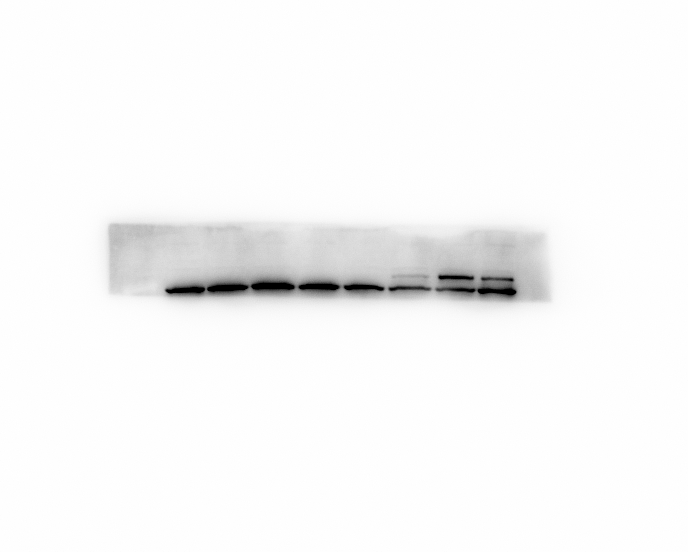

Supplement: Figure 5—source data 1. [file elife-97206-fig5-data1.zip › Figure 5-source data 1/Figure 5 b/Original file for the Western blot analysis in Figure 5b (anti-ATG6-mCherry) .tif]

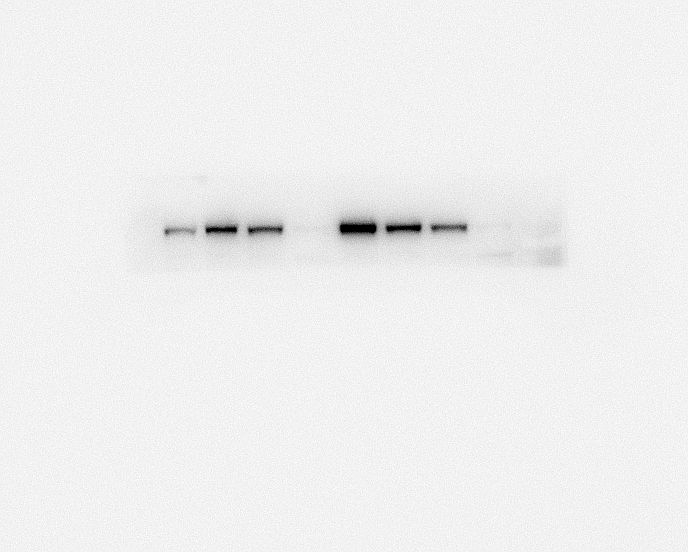

Supplement: Figure 5—source data 1. [file elife-97206-fig5-data1.zip › Figure 5-source data 1/Figure 5 b/Original file for the Western blot analysis in Figure 5b (anti-NPR1-GFP) .tif]

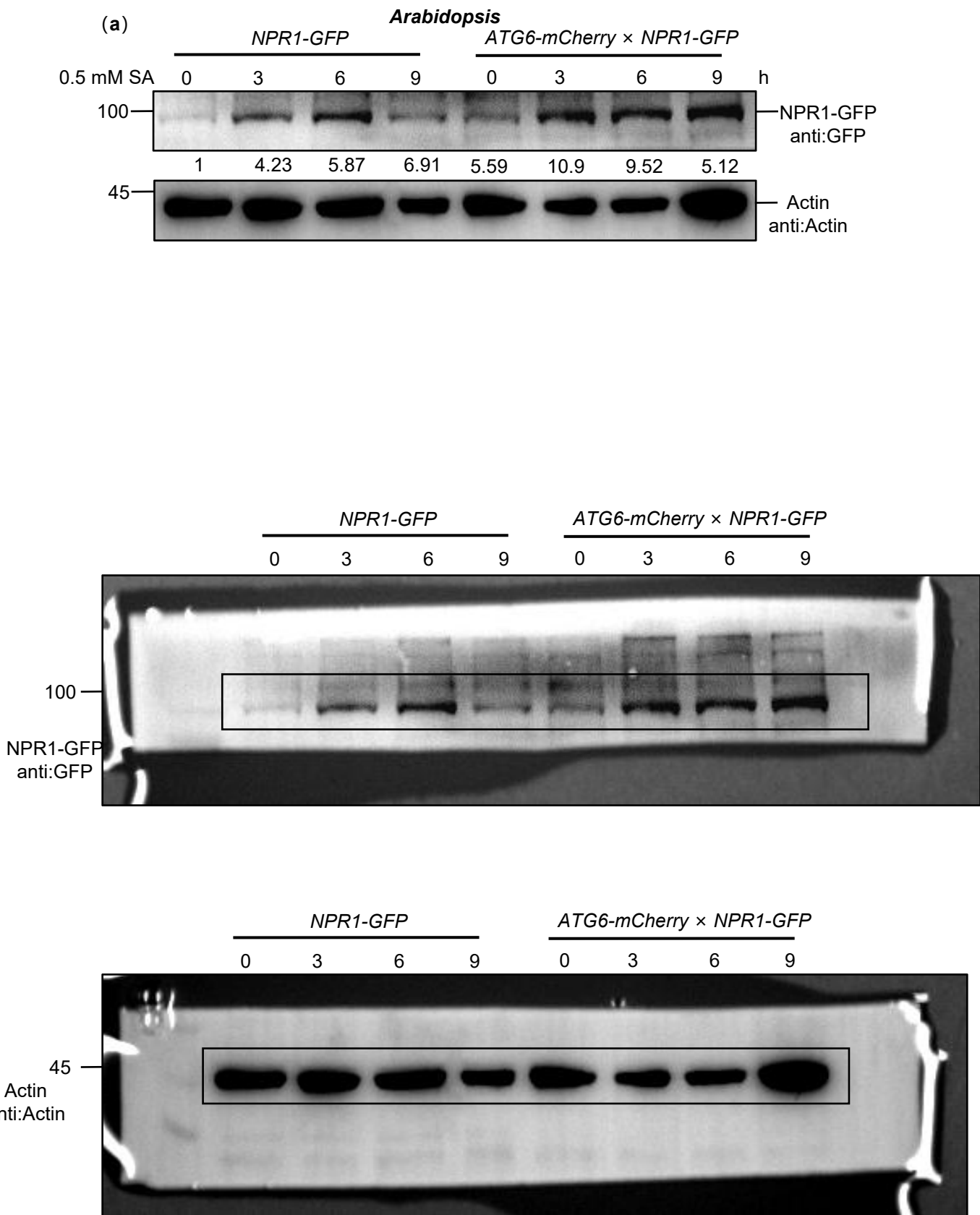

Figure 5. ATG6 increases the NPR1 protein levels and the formation of SINC-like condensates.

Supplement: Figure 5—source data 2. [file elife-97206-fig5-data2.zip › Figure 5-source data 2/Figure 5 a.pdf]

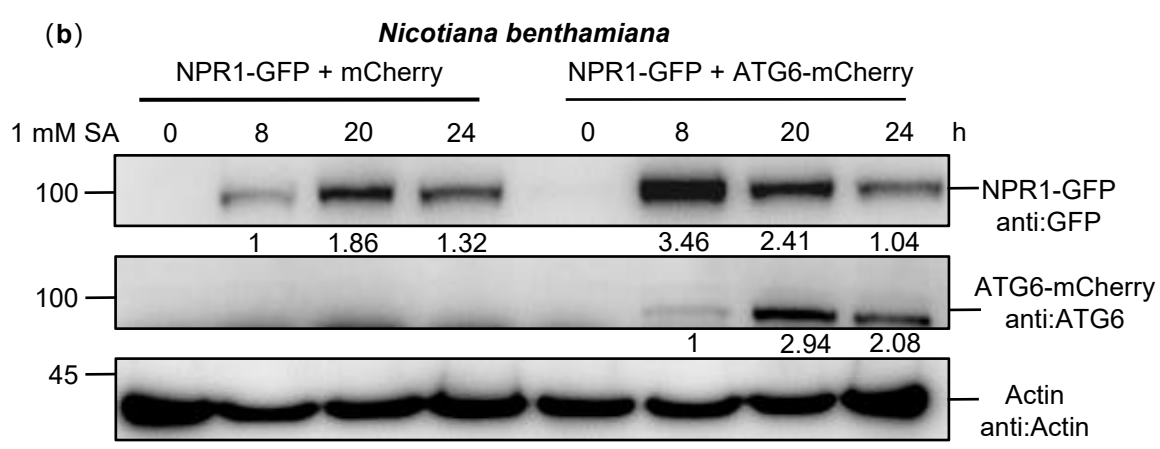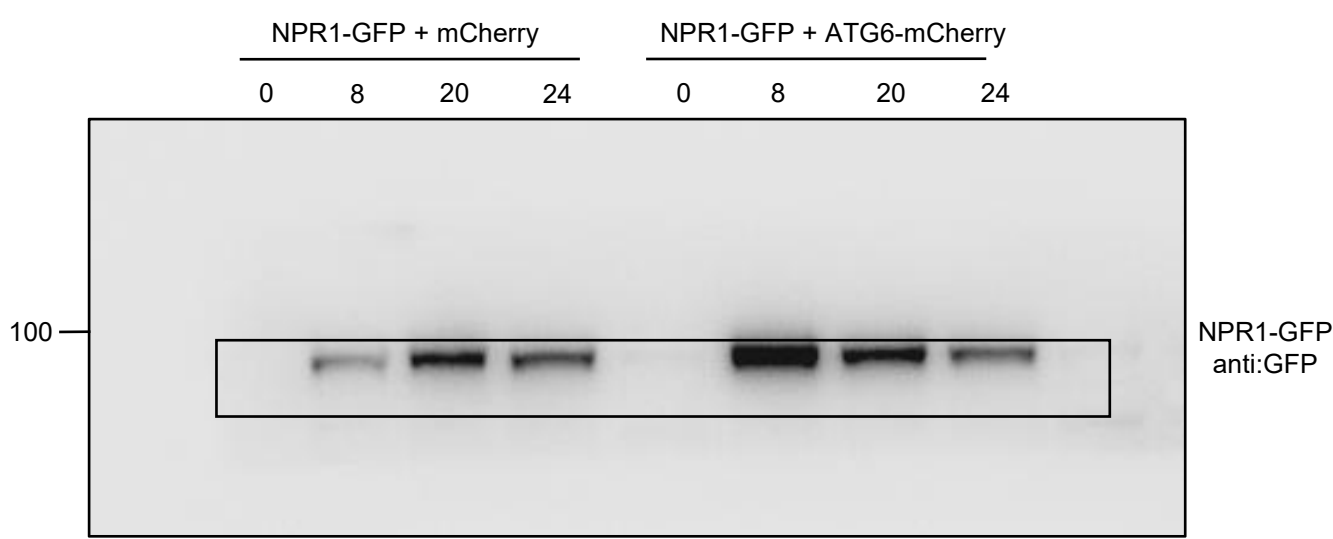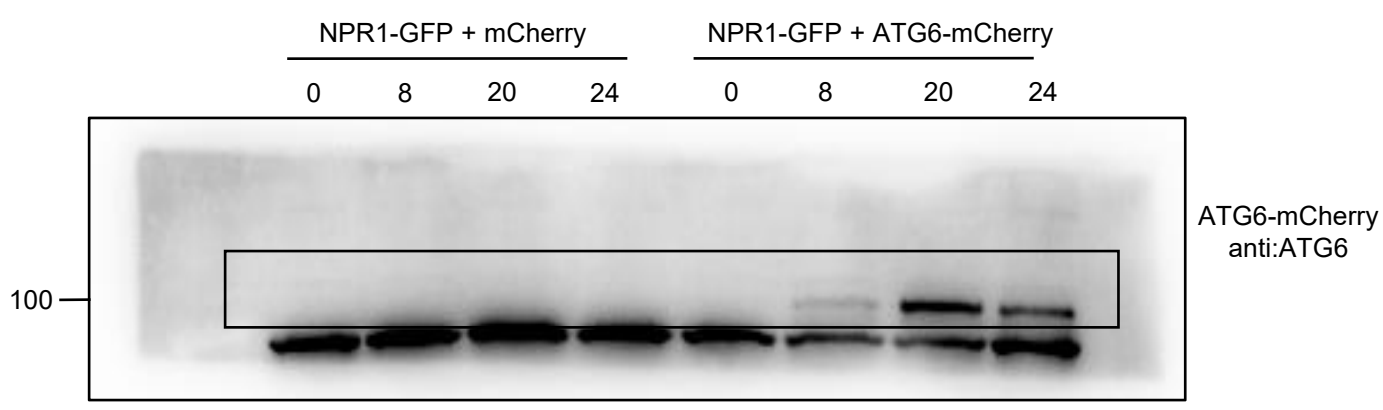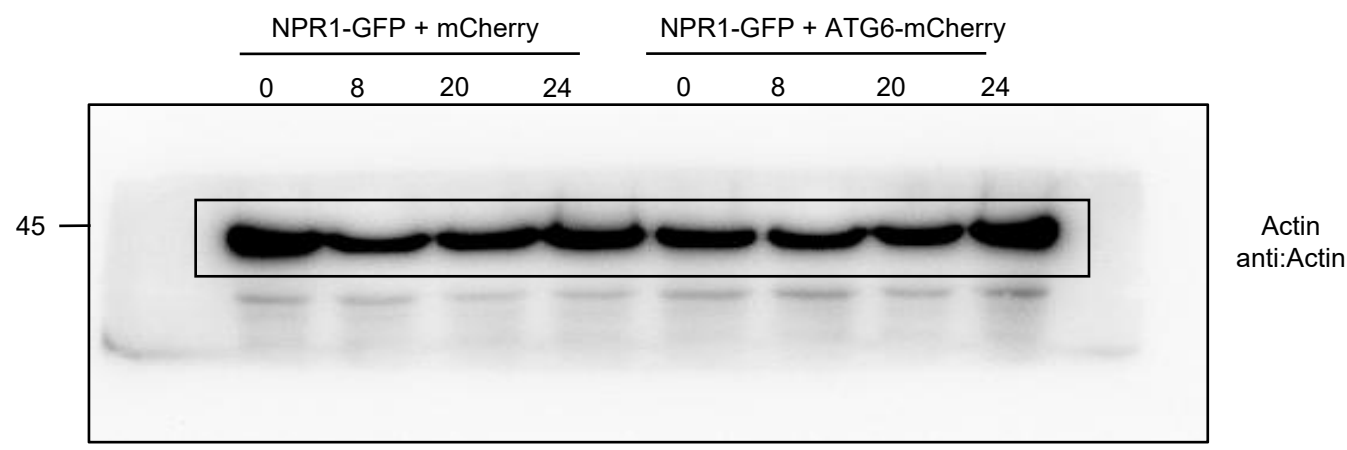

Figure 5. ATG6 increases the NPR1 protein levels and the formation of SINC-like condensates.

Supplement: Figure 5—source data 2. [file elife-97206-fig5-data2.zip › Figure 5-source data 2/Figure 5 b.pdf]

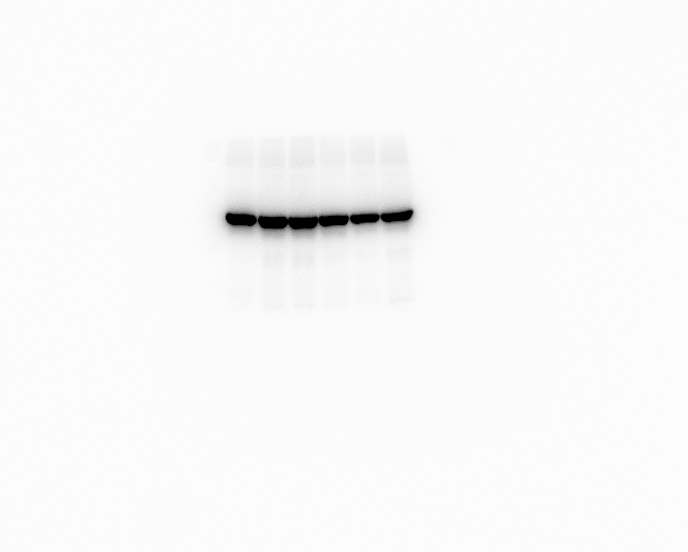

Supplement: Figure 5—figure supplement 1—source data 1. [file elife-97206-fig5-figsupp1-data1.zip › Figure 5-figure supplement 1-source data 1/Original file for the Western blot in Figure 5-figure supplement 1 (anti-Actin).tif]

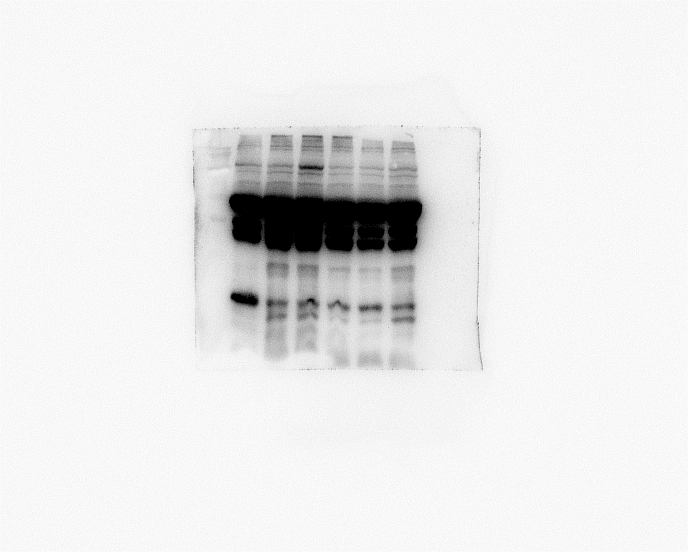

Supplement: Figure 5—figure supplement 1—source data 1. [file elife-97206-fig5-figsupp1-data1.zip › Figure 5-figure supplement 1-source data 1/Original file for the Western blot in Figure 5-figure supplement 1 (anti-NPR1-GFP).tif]

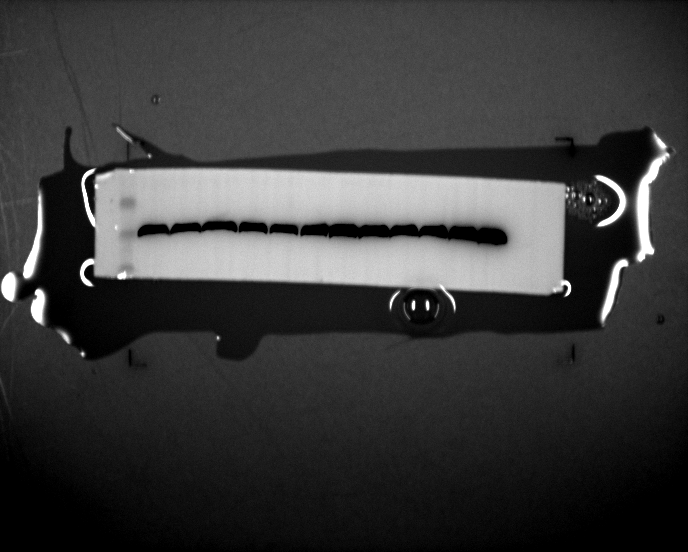

Supplement: Figure 6—source data 1. [file elife-97206-fig6-data1.zip › Figure 6-source data 1/Figure 6 a/Original file for the Western blot analysis in Figure 6a (anti-Actin) .tif]

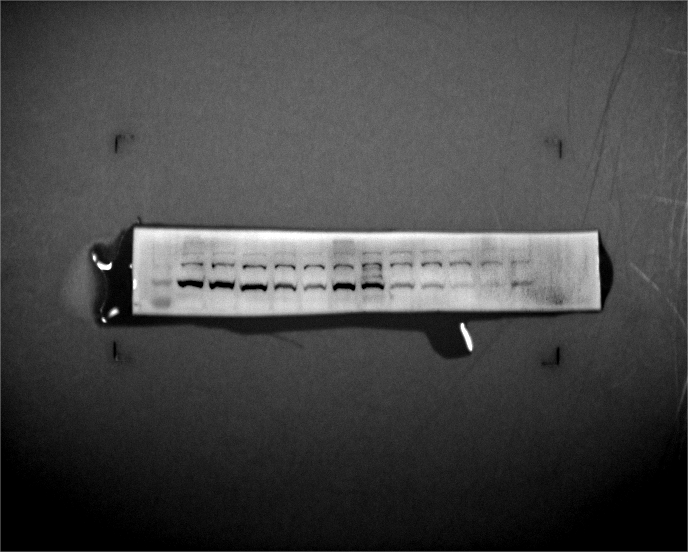

Supplement: Figure 6—source data 1. [file elife-97206-fig6-data1.zip › Figure 6-source data 1/Figure 6 a/Original file for the Western blot analysis in Figure 6a (anti-NPR1-GFP) .tif]

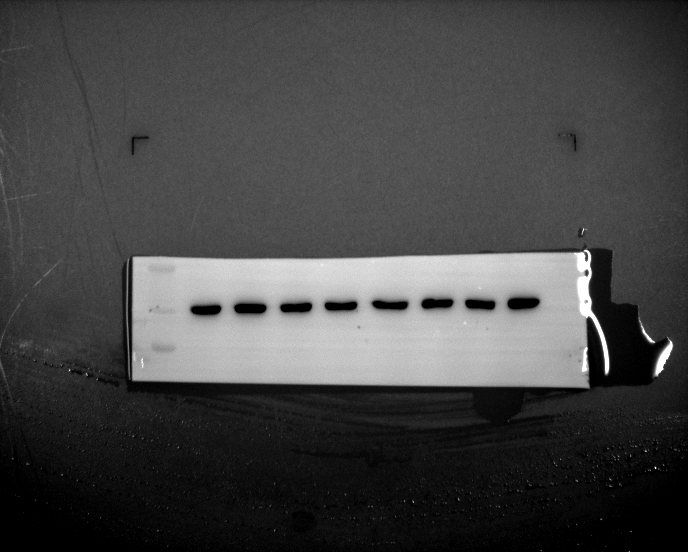

Supplement: Figure 6—source data 1. [file elife-97206-fig6-data1.zip › Figure 6-source data 1/Figure 6 c/Original file for the Western blot analysis in Figure 6c (anti-Actin) .tif]

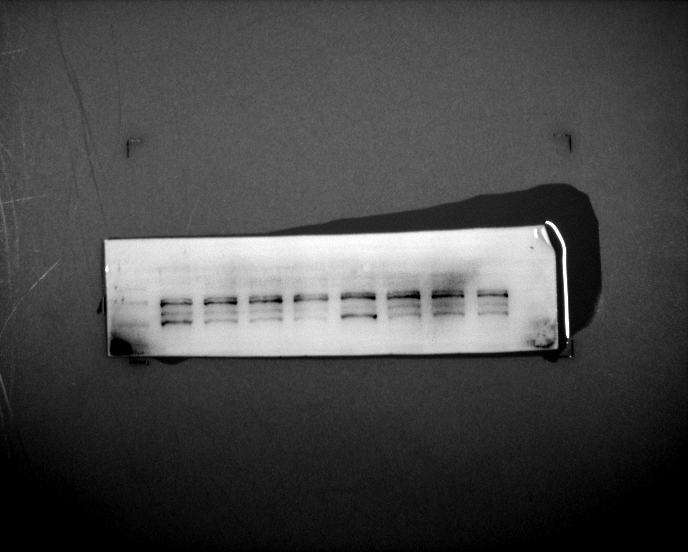

Supplement: Figure 6—source data 1. [file elife-97206-fig6-data1.zip › Figure 6-source data 1/Figure 6 c/Original file for the Western blot analysis in Figure 6c (anti-NPR1-GFP) .tif]

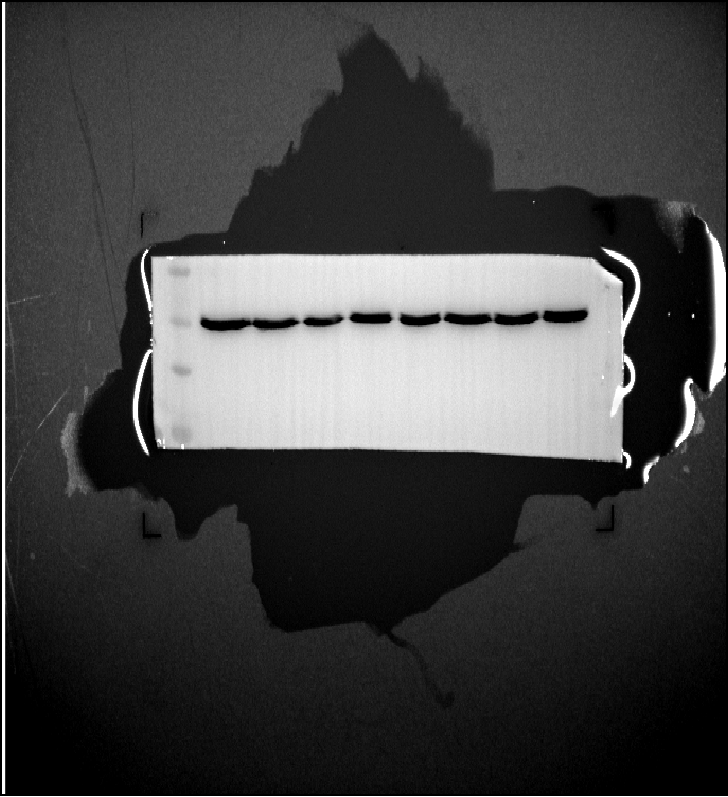

Supplement: Figure 6—source data 1. [file elife-97206-fig6-data1.zip › Figure 6-source data 1/Figure 6 e/Original file for the Western blot analysis in Figure 6e (anti-Actin) .tif]

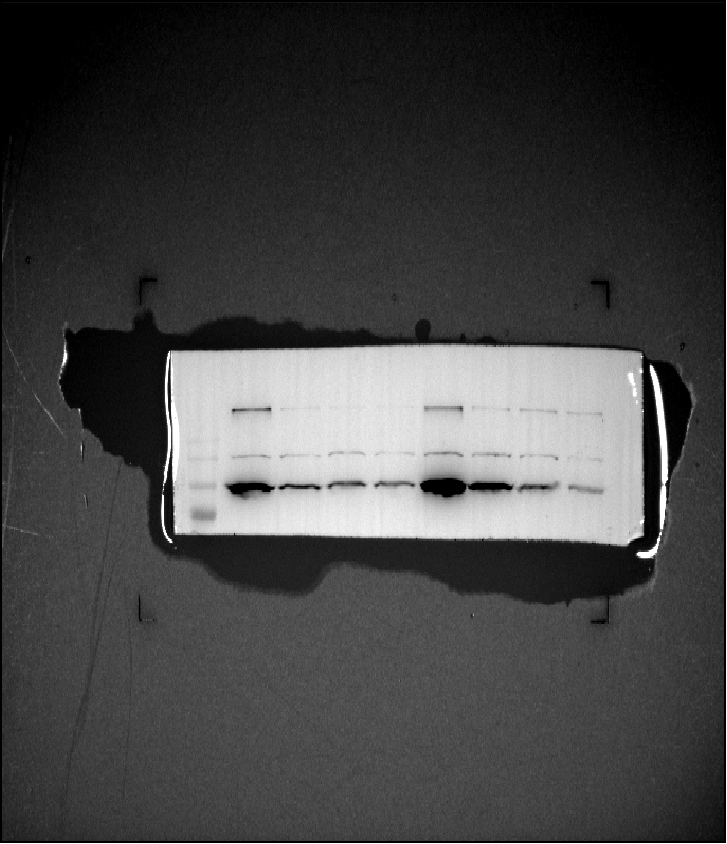

Supplement: Figure 6—source data 1. [file elife-97206-fig6-data1.zip › Figure 6-source data 1/Figure 6 e/Original file for the Western blot analysis in Figure 6e (anti-NPR1-GFP) .tif]

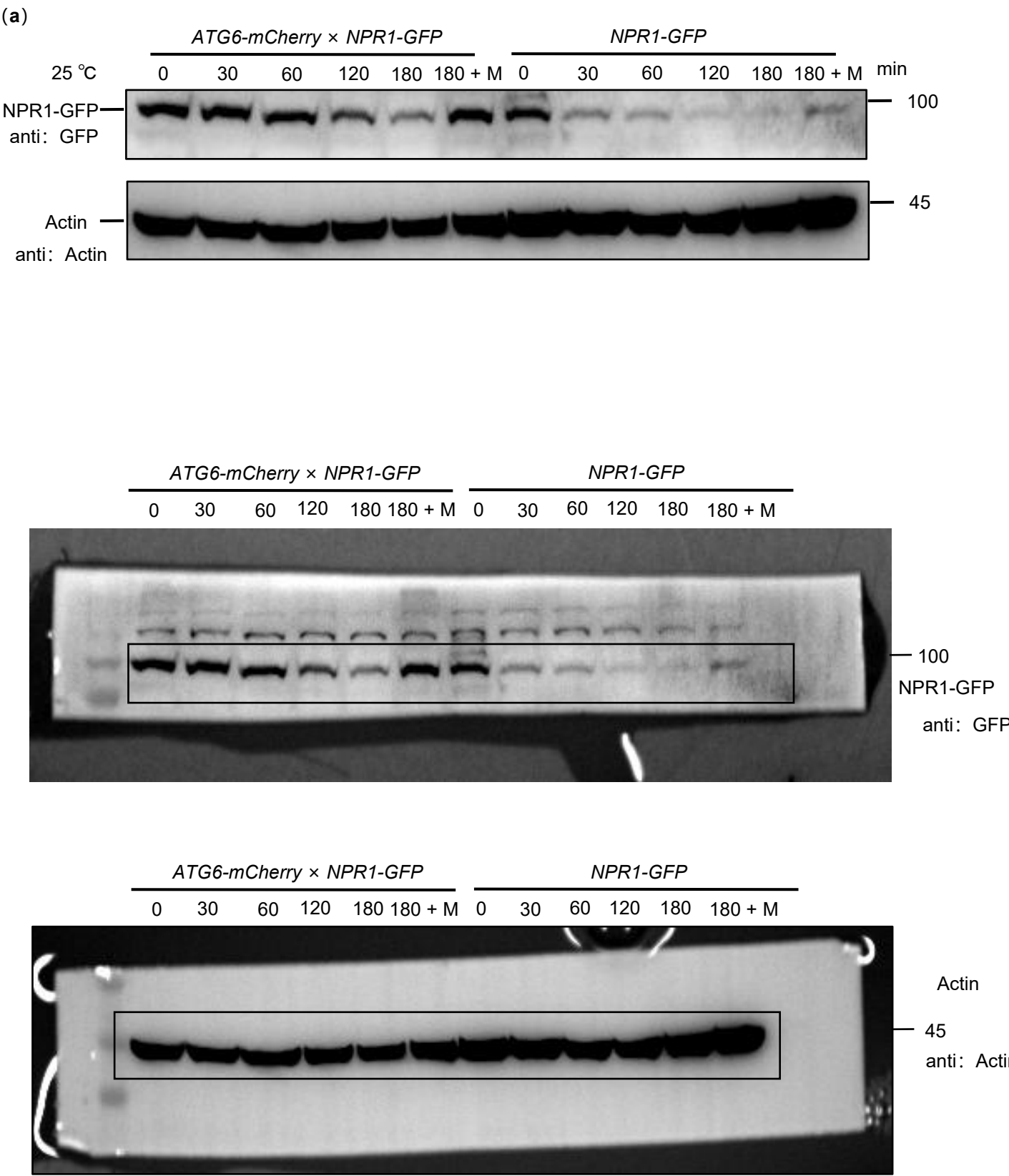

Figure 6. ATG6 improves the protein stability of NPR1.

Supplement: Figure 6—source data 2. [file elife-97206-fig6-data2.zip › Figure 6-source data 2/Figure 6 a.pdf]

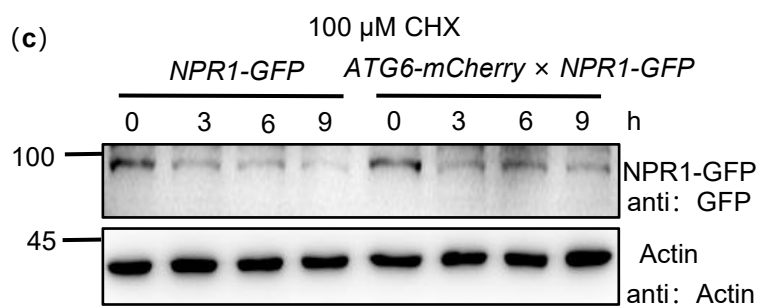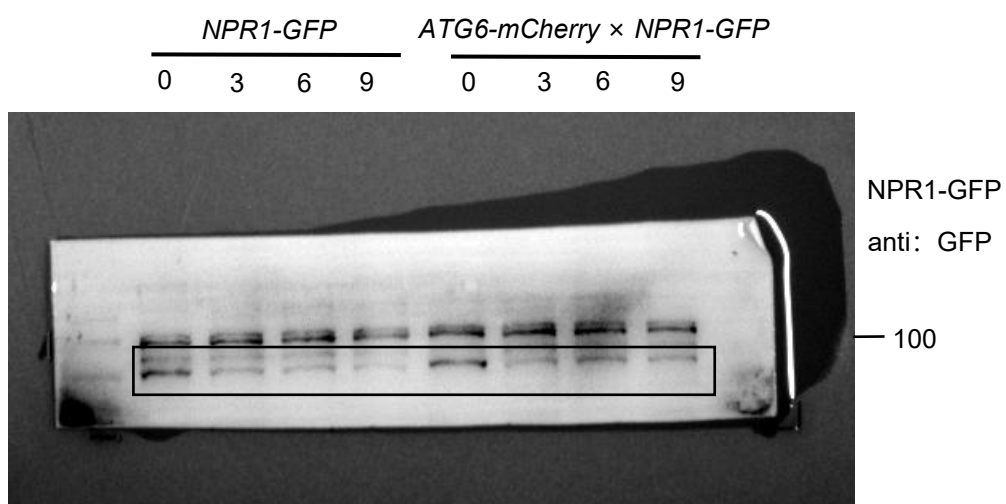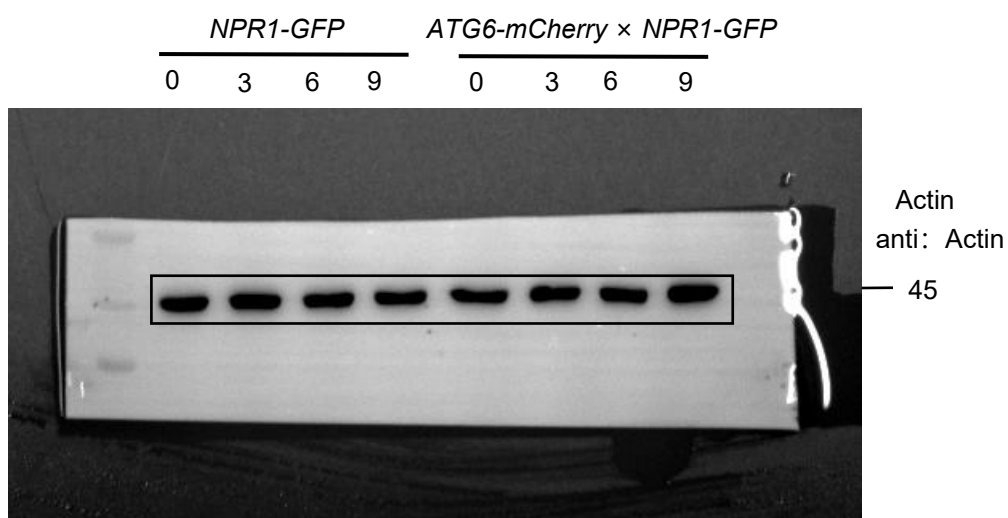

Figure 6. ATG6 improves the protein stability of NPR1.

Supplement: Figure 6—source data 2. [file elife-97206-fig6-data2.zip › Figure 6-source data 2/Figure 6 c.pdf]

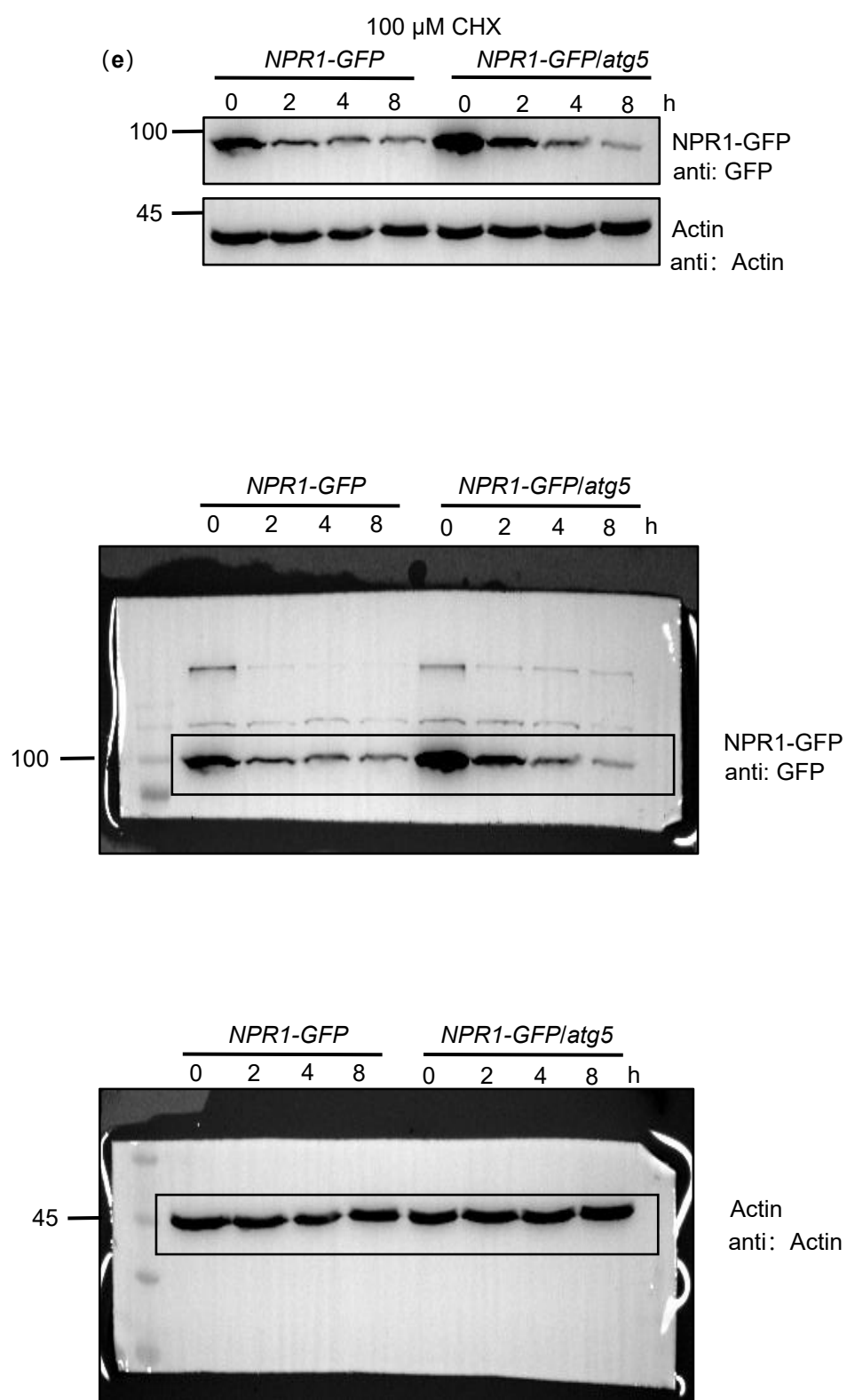

Figure 6. ATG6 improves the protein stability of NPR1.

Supplement: Figure 6—source data 2. [file elife-97206-fig6-data2.zip › Figure 6-source data 2/Figure 6 e.pdf]

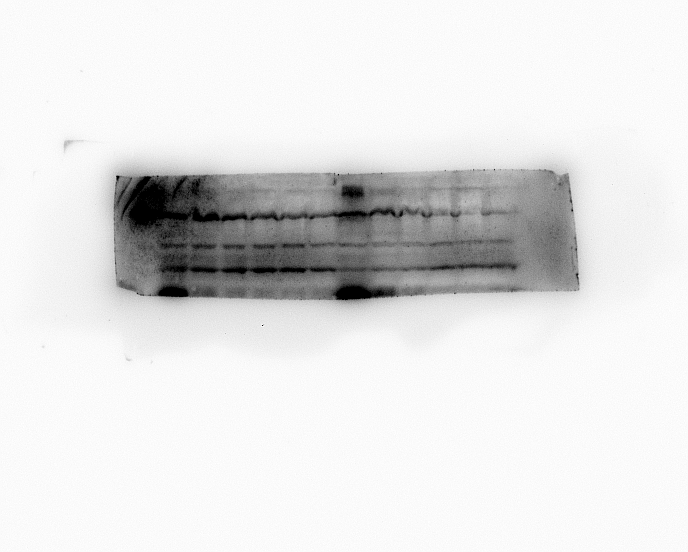

Supplement: Figure 6—figure supplement 1—source data 1. [file elife-97206-fig6-figsupp1-data1.zip › Figure 6-figure supplement 1-source data 1/Original file for the Western blot analysis in Figure 6-figure supplement 1 (anti-NPR1-GFP).tif]

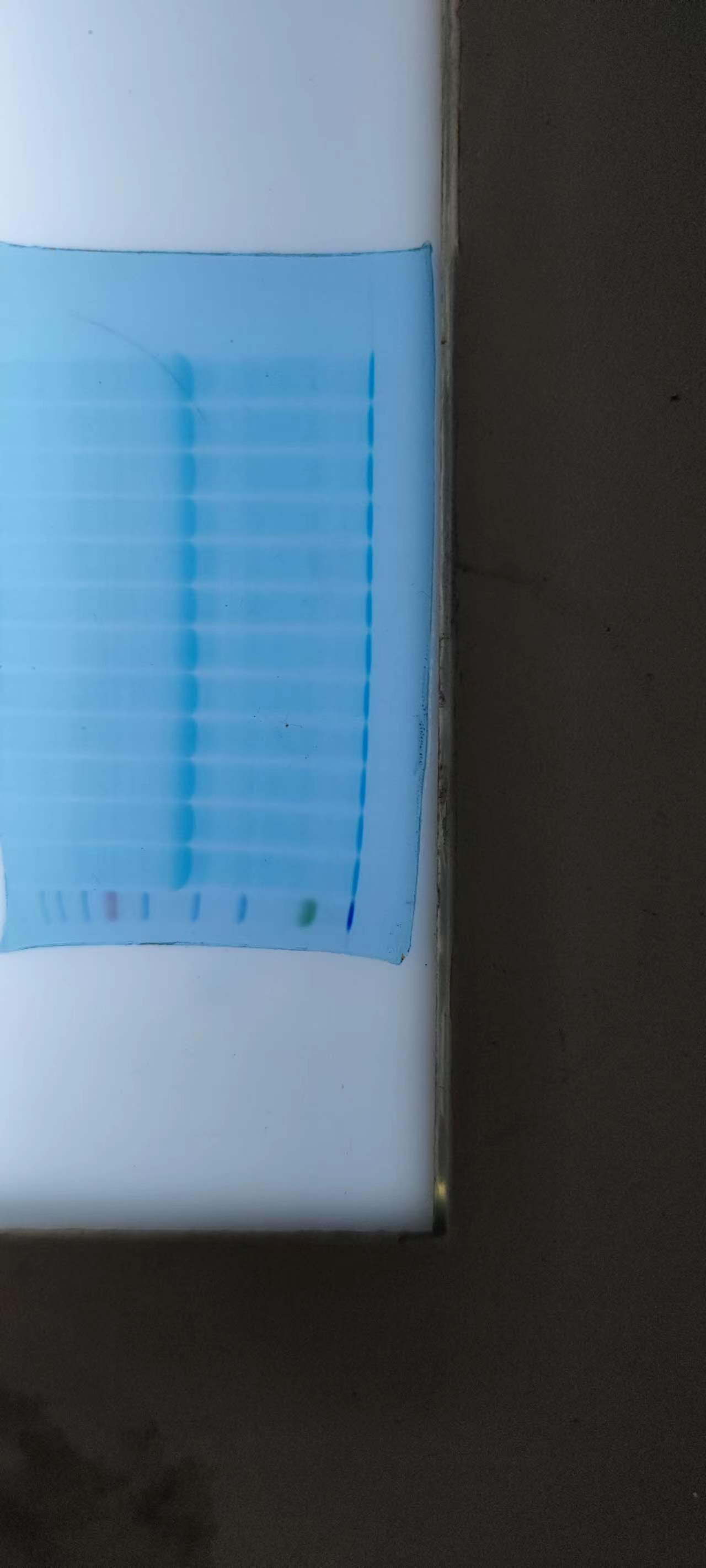

Supplement: Figure 6—figure supplement 1—source data 1. [file elife-97206-fig6-figsupp1-data1.zip › Figure 6-figure supplement 1-source data 1/Original file for the Western blot analysis in Figure 6-figure supplement 1 (CBB).tif]

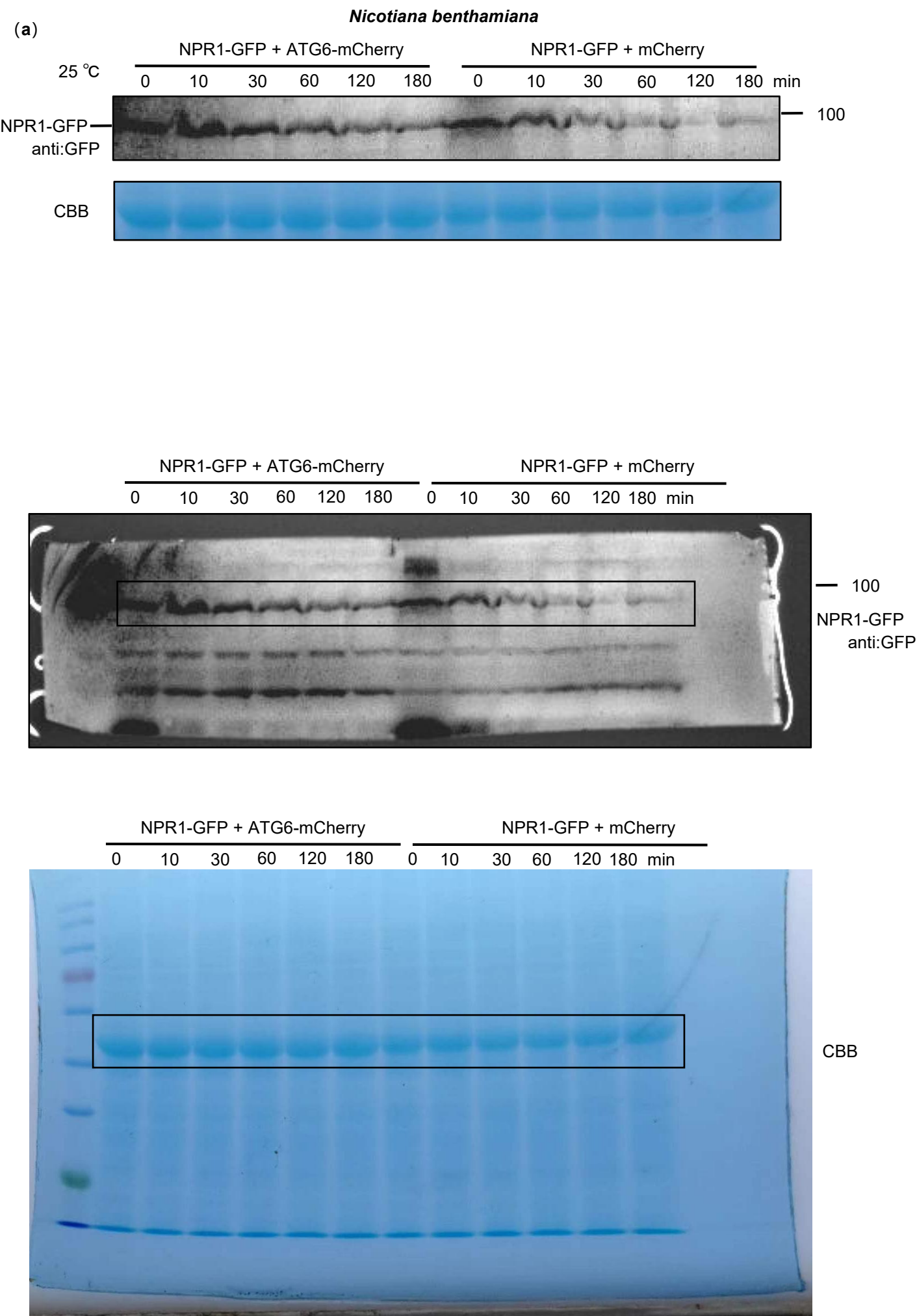

Figure 6-figure supplement 1 ATG6 improves the protein stability of NPR1 in *N. benthamiana*.

Supplement: Figure 6—figure supplement 1—source data 2. [file elife-97206-fig6-figsupp1-data2.zip › Figure 6-figure supplement 1-source data 2/Figure 6-figure supplement 1.pdf]

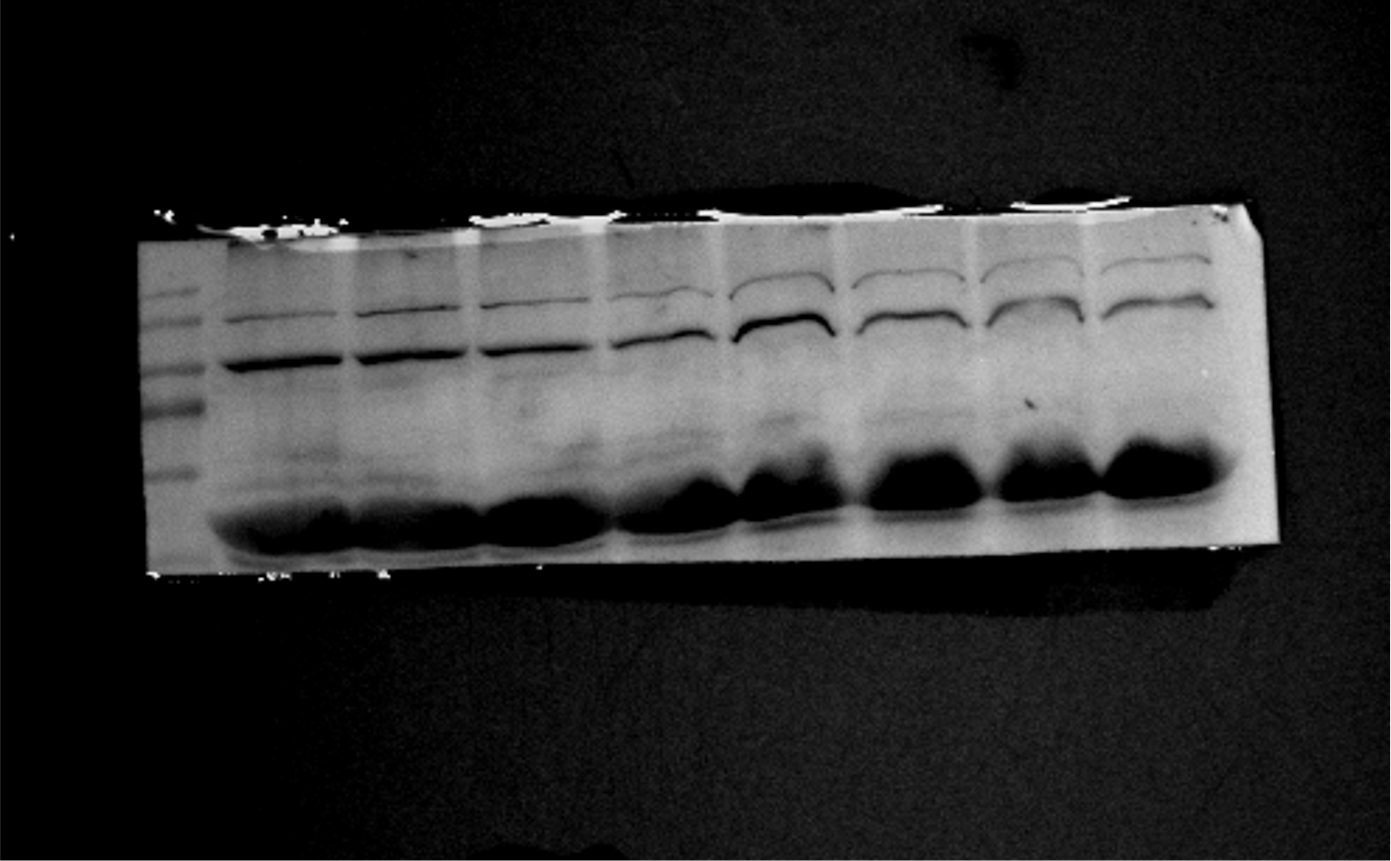

Supplement: Figure 6—figure supplement 2—source data 1. [file elife-97206-fig6-figsupp2-data1.zip › Figure 6-figure supplement 2-source data 1/Original file for the Western blot analysis in Figure 6-figure supplement 2 (anti-NPR1-GFP and Rubiso).tif]

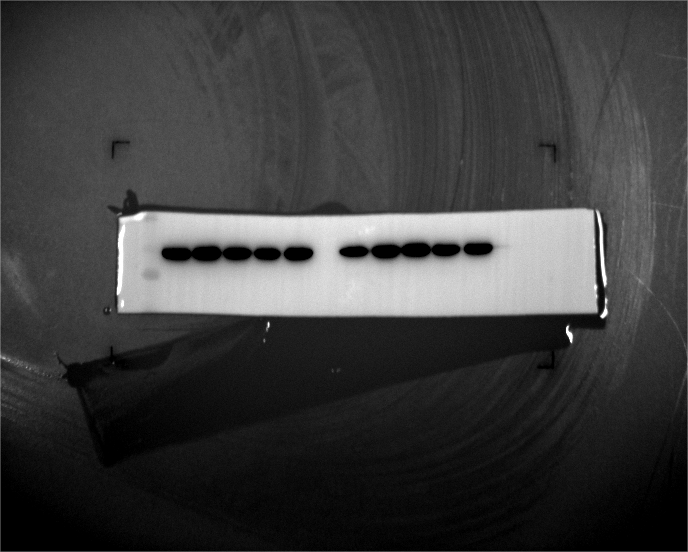

Supplement: Figure 7—source data 1. [file elife-97206-fig7-data1.zip › Figure 7-source data 1/Figure 7c/Original file for the Western blot analysis in Figure 7c (anti-Actin) .tif]

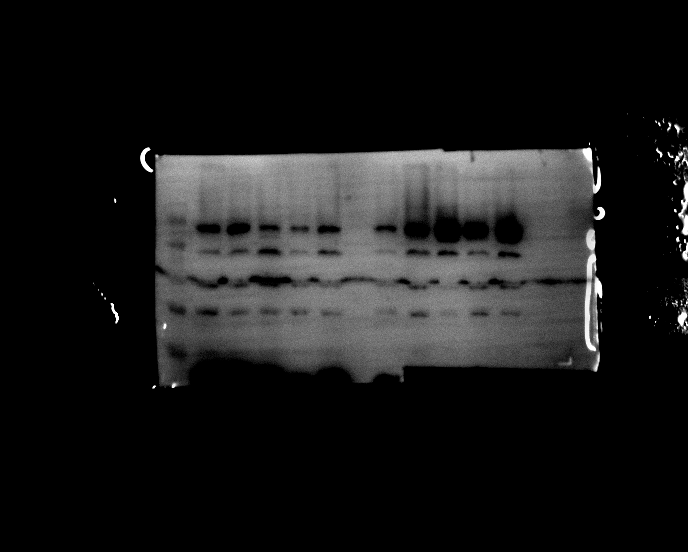

Supplement: Figure 7—source data 1. [file elife-97206-fig7-data1.zip › Figure 7-source data 1/Figure 7c/Original file for the Western blot analysis in Figure 7c (anti-ATG6) .tif]

(c)

0.5 mM SA

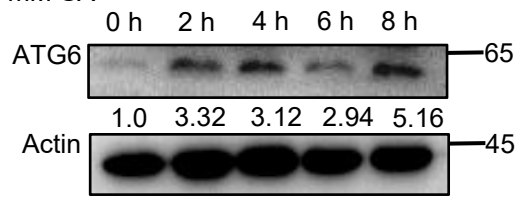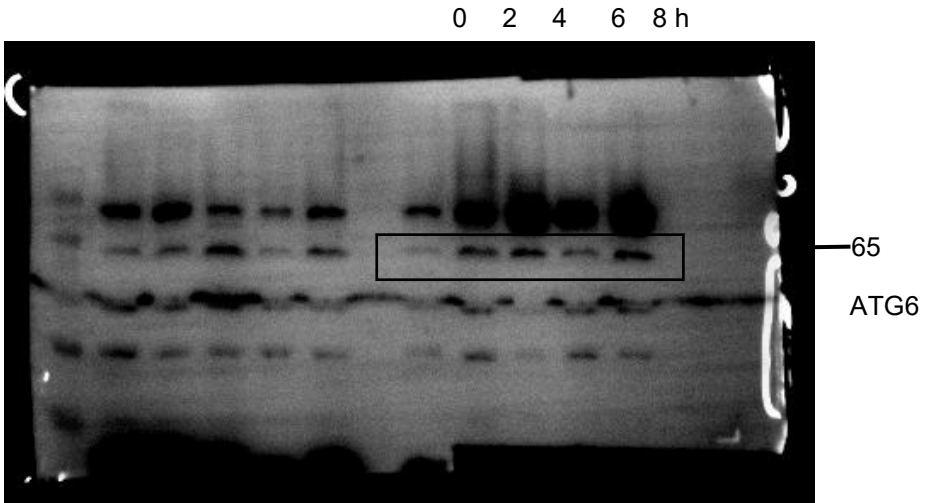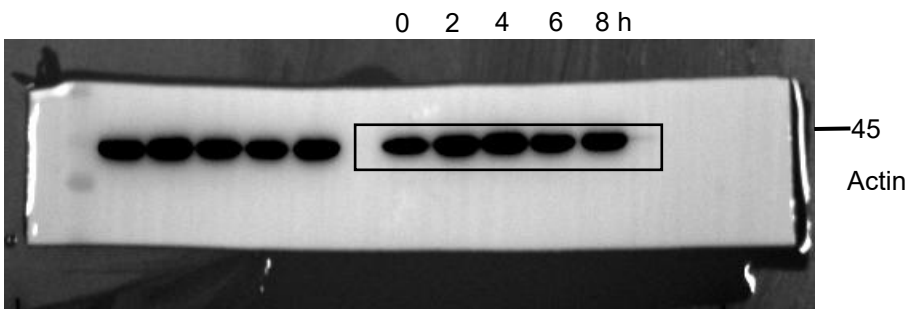

Figure 7. ATG6 and NPR1 jointly inhibit the growth of *Pst* DC3000/*avrRps4*

Supplement: Figure 7—source data 2. [file elife-97206-fig7-data2.zip › Figure 7-source data 2/Figure 7c.pdf]

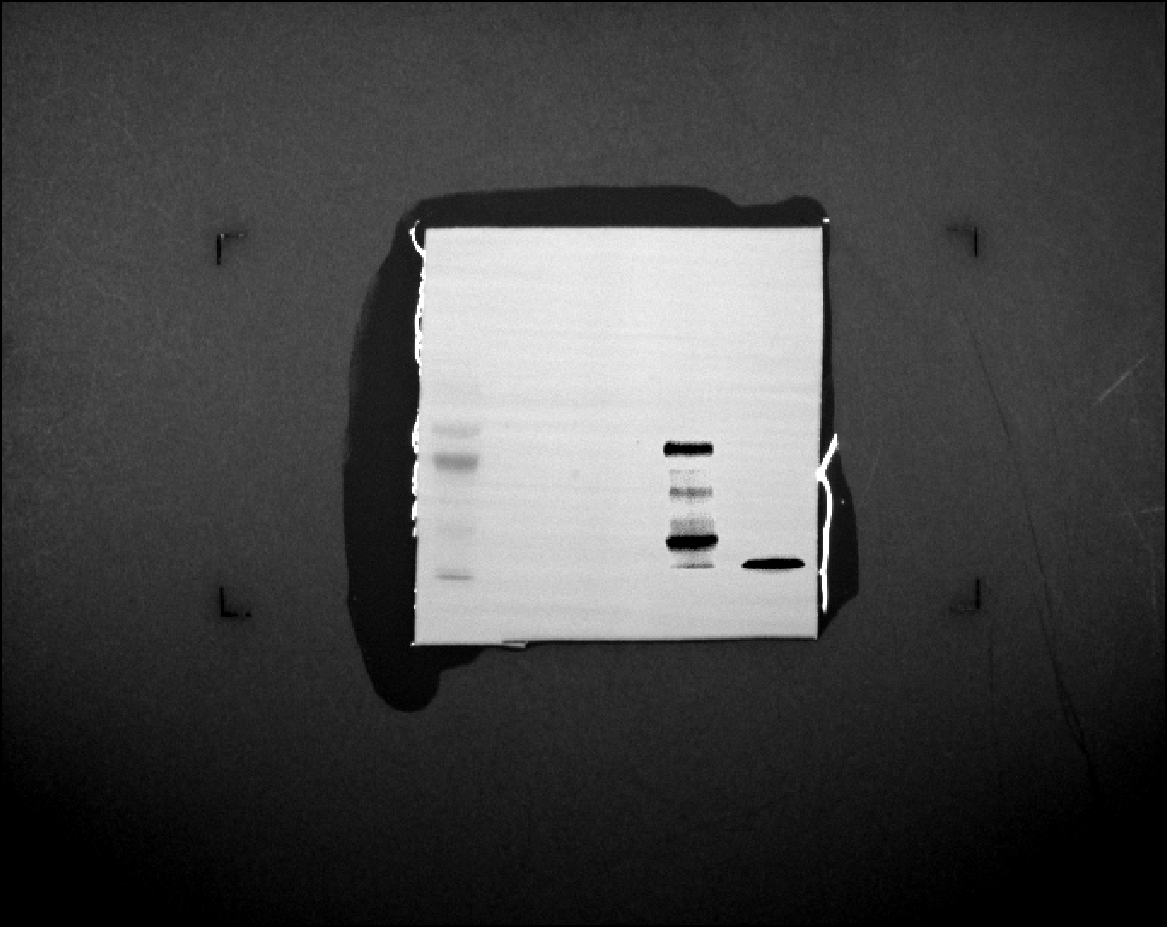

Supplement: Figure 7—figure supplement 1—source data 1. [file elife-97206-fig7-figsupp1-data1.zip › Figure 7-figure supplement 1 -source data 1/Figure 7-figure supplement 1a/Original file for the Western blot in Figure 7-figure supplement 1a (anti-GST-ATG6).tif]

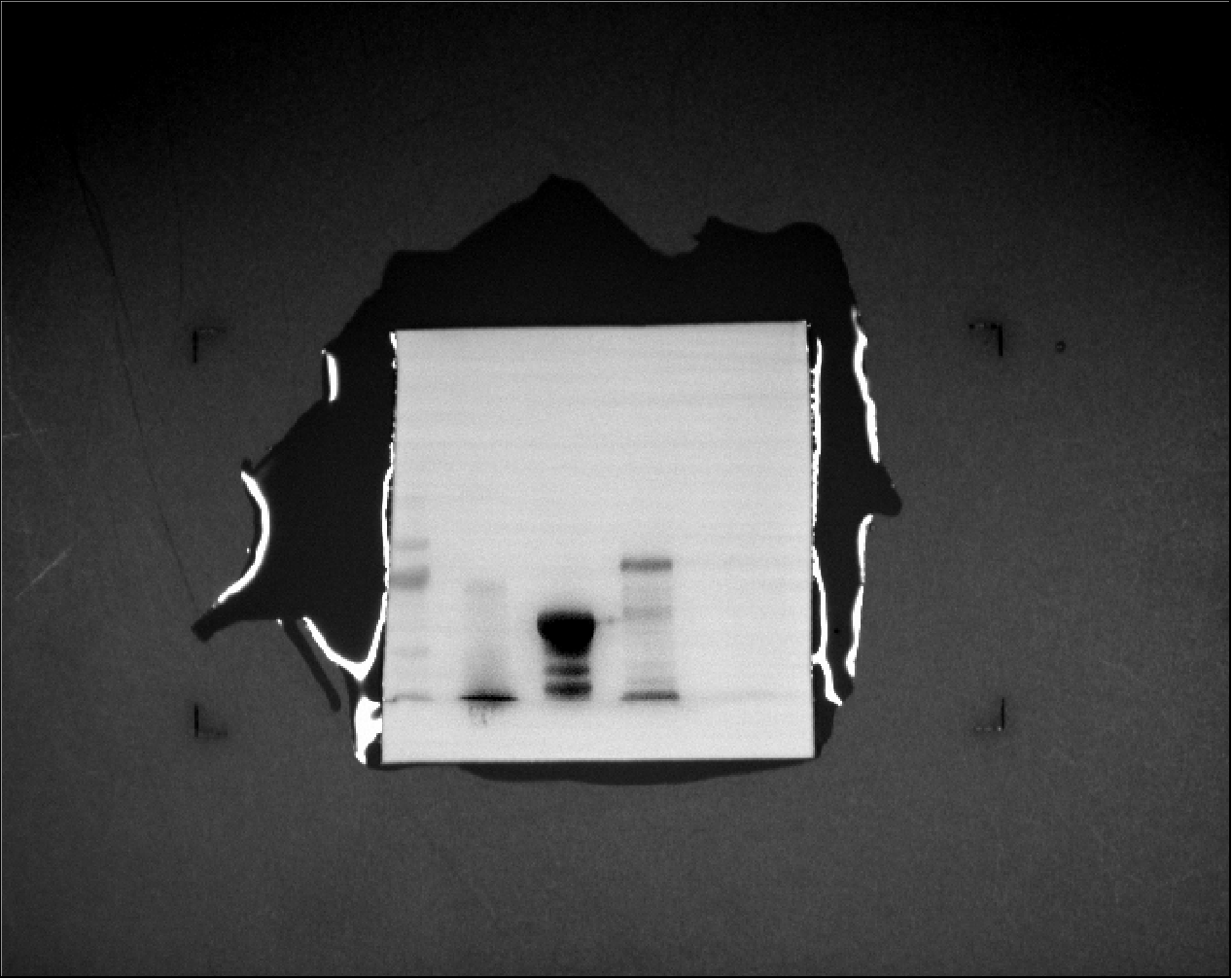

Supplement: Figure 7—figure supplement 1—source data 1. [file elife-97206-fig7-figsupp1-data1.zip › Figure 7-figure supplement 1 -source data 1/Figure 7-figure supplement 1a/Original file for the Western blot in Figure 7-figure supplement 1a (anti-GST-ATG6,GST-SnRK2.8).tif]

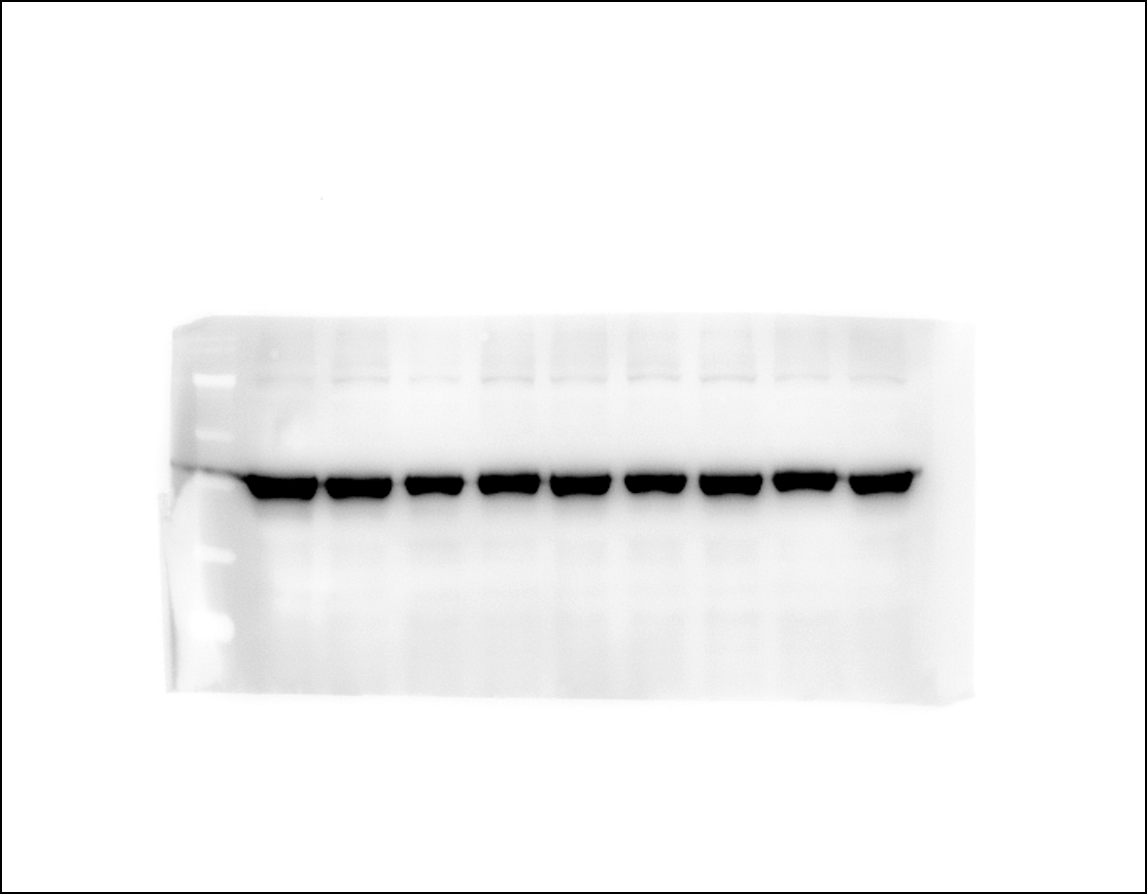

Supplement: Figure 7—figure supplement 1—source data 1. [file elife-97206-fig7-figsupp1-data1.zip › Figure 7-figure supplement 1 -source data 1/Figure 7-figure supplement 1b/Original file for the Western blot in Figure 7-figure supplement 1b (anti-Actin).tif]

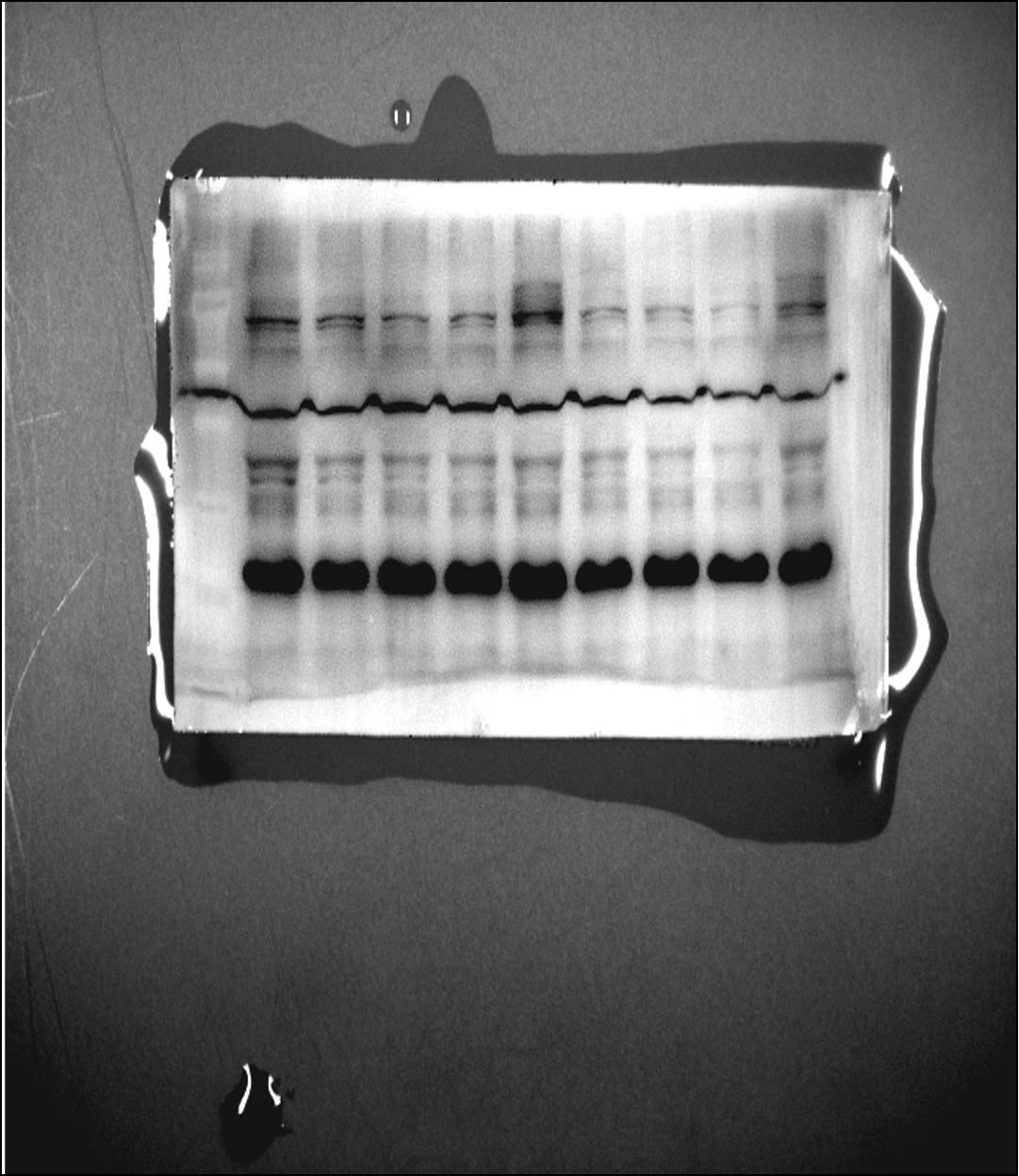

Supplement: Figure 7—figure supplement 1—source data 1. [file elife-97206-fig7-figsupp1-data1.zip › Figure 7-figure supplement 1 -source data 1/Figure 7-figure supplement 1b/Original file for the Western blot in Figure 7-figure supplement 1b (anti-ATG6).tif]

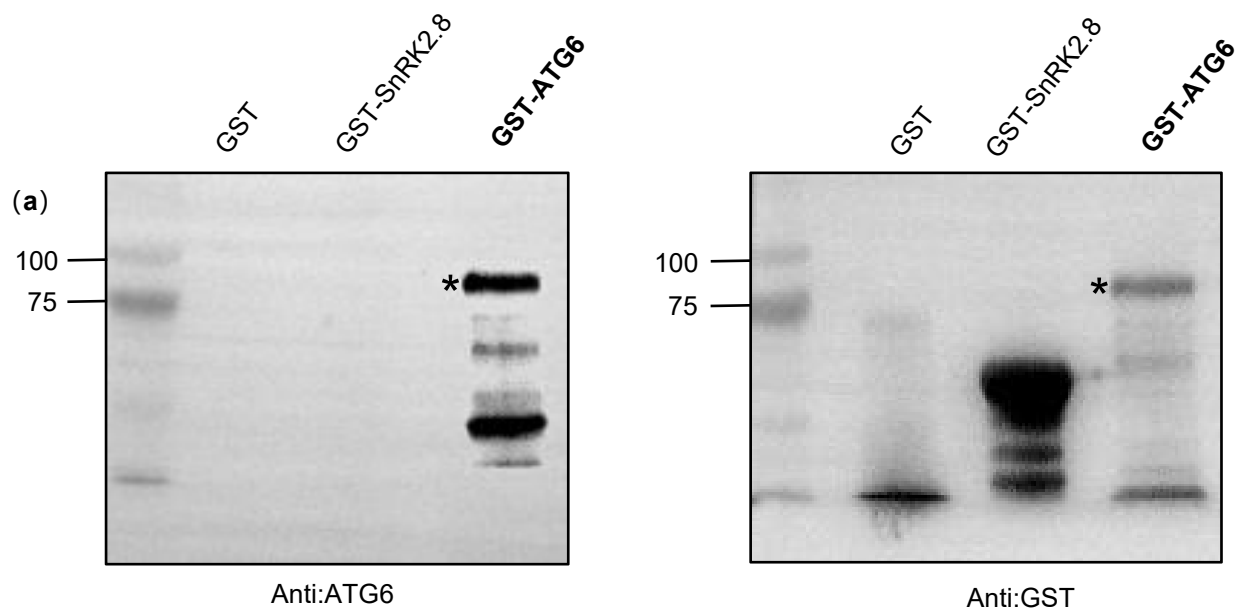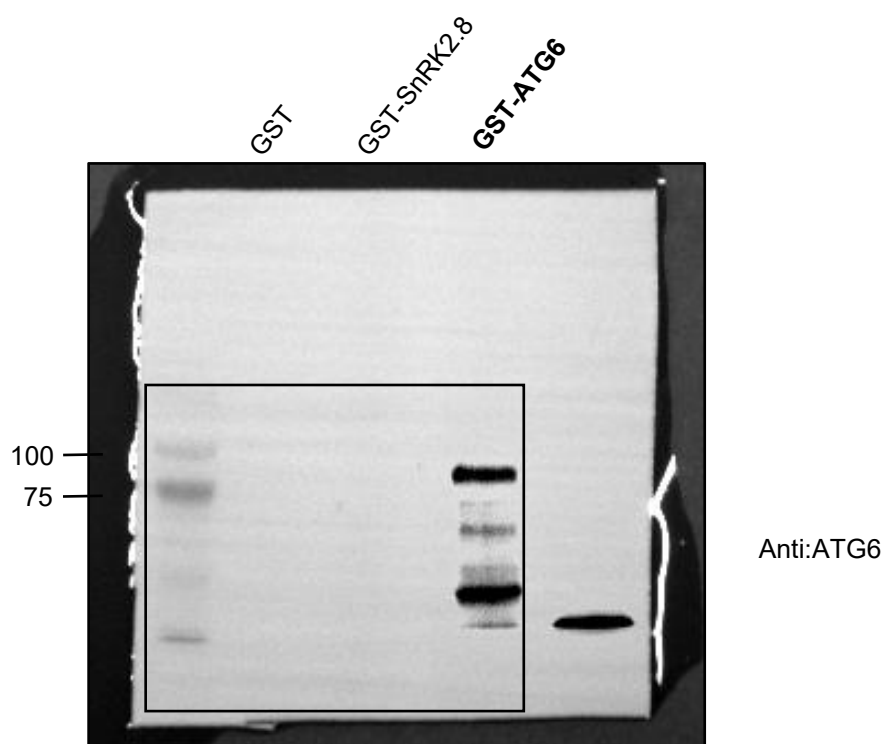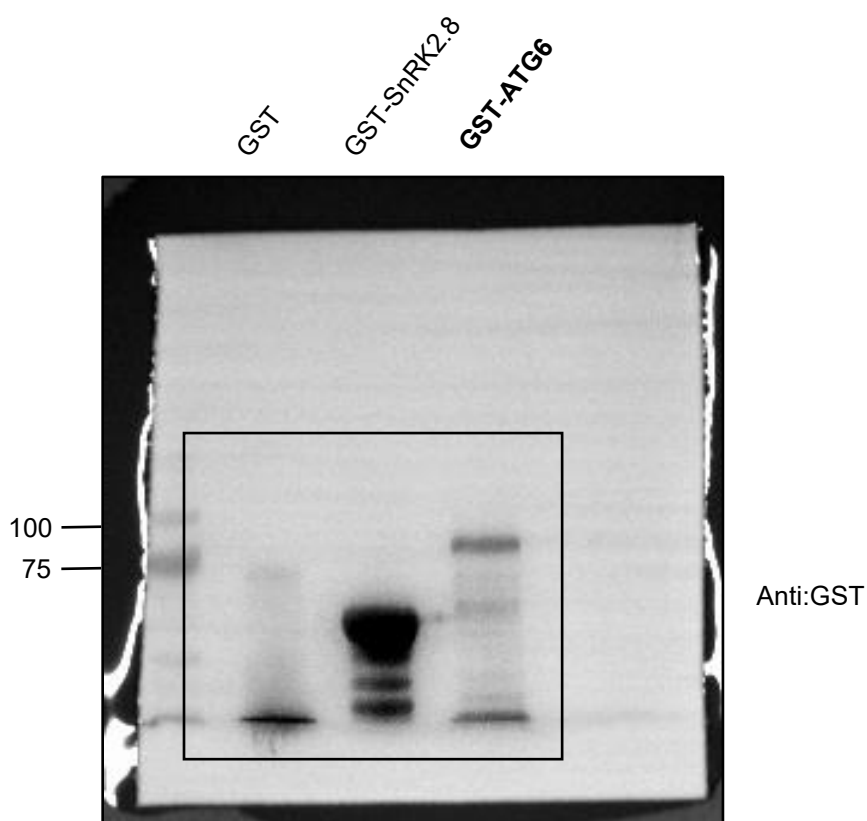

Figure 7-figure supplement 1. Verification of ATG6 antibody specificity.

Supplement: Figure 7—figure supplement 1—source data 2. [file elife-97206-fig7-figsupp1-data2.zip › Figure 7-figure supplement 1 -source data 2/Figure 7-figure supplement 1a.pdf]

(b)

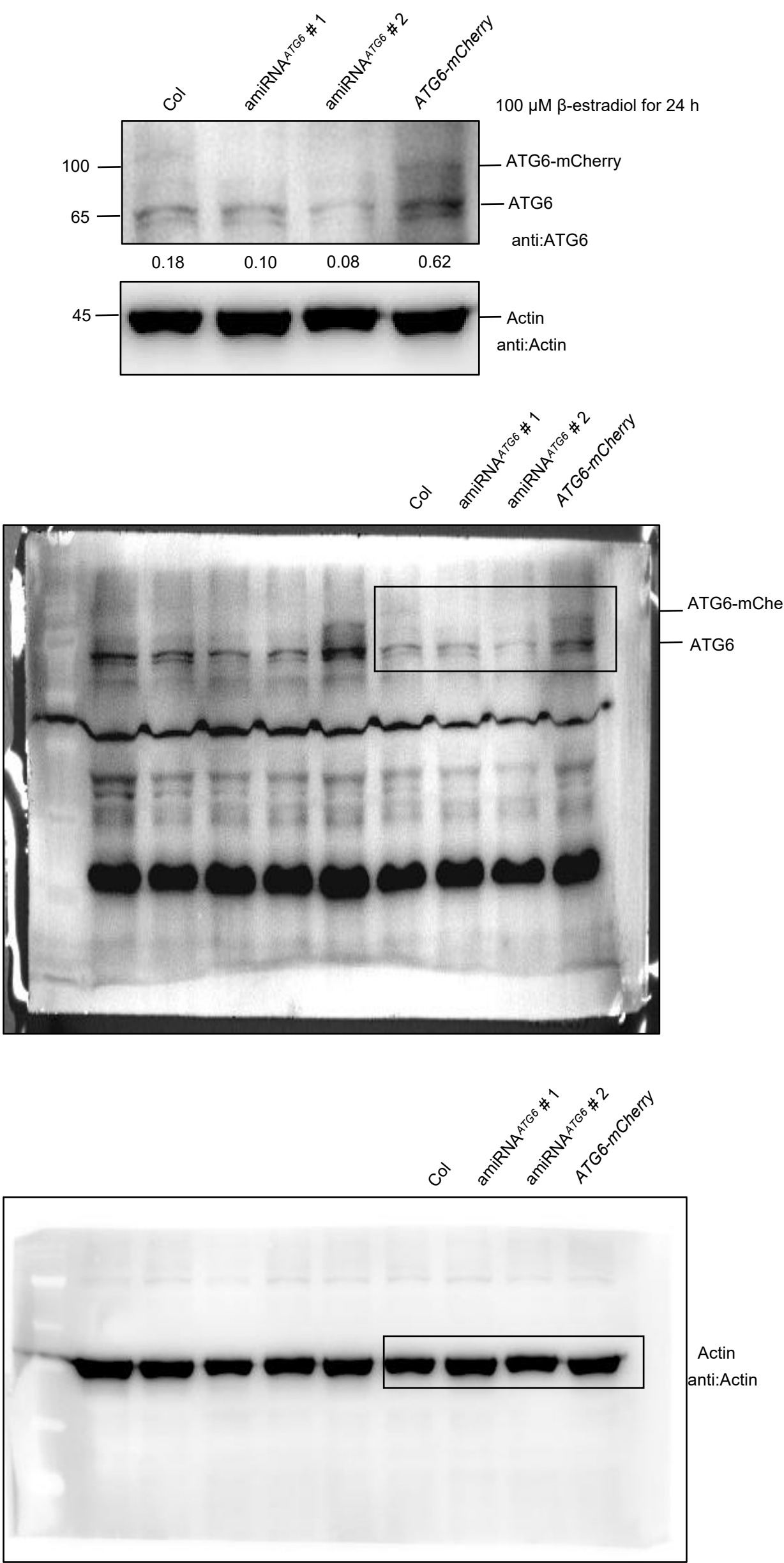

Figure 7-figure supplement 1. Verification of ATG6 antibody specificity.

Supplement: Figure 7—figure supplement 1—source data 2. [file elife-97206-fig7-figsupp1-data2.zip › Figure 7-figure supplement 1 -source data 2/Figure 7-figure supplement 1b.pdf]
