# Supplementary material for: ATG6 interacting with NPR1 increases Arabidopsis thaliana resistance to Pst DC3000/avrRps4 by increasing its nuclear accumulation and stability
Source: eLife. 2025 Mar 4;13:RP97206. doi: 10.7554/eLife.97206 (PMC11879114; doi:10.7554/eLife.97206)
Supplement: Figure 2—figure supplement 3—source data 2. [file elife-97206-fig2-figsupp3-data2.zip › Figure 2-figure supplement 3-source data 2/Figure 2-figure supplement 3.pdf]

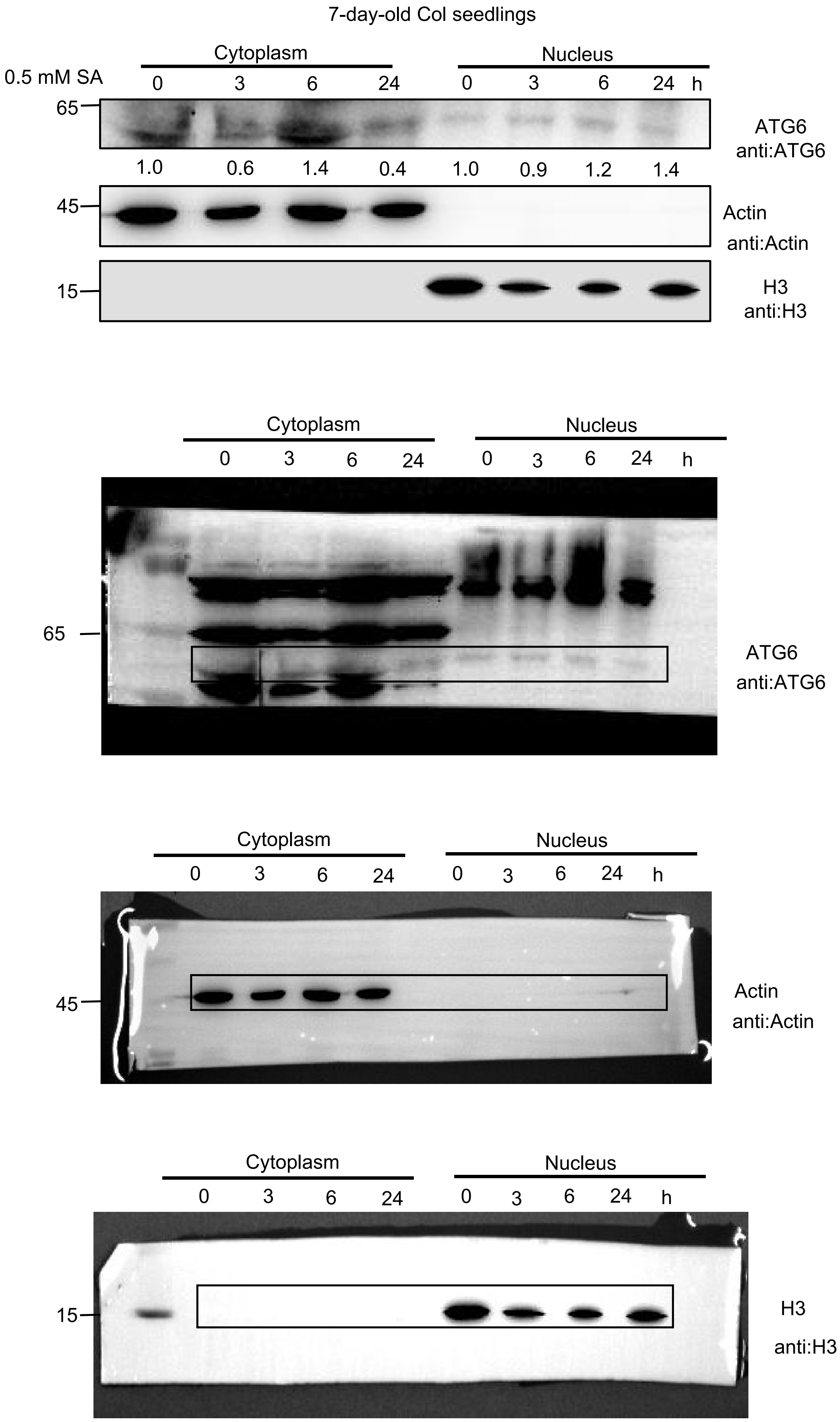

Figure 2-figure supplement 3 Subcellular fractionation of endogenous ATG6 in Col after 0.5 mM SA treatment for 0, 3, 6 and 20 h.
