# Supplementary material for: ATG6 interacting with NPR1 increases Arabidopsis thaliana resistance to Pst DC3000/avrRps4 by increasing its nuclear accumulation and stability
Source: eLife. 2025 Mar 4;13:RP97206. doi: 10.7554/eLife.97206 (PMC11879114; doi:10.7554/eLife.97206)
Supplement: Figure 3—figure supplement 2—source data 2. [file elife-97206-fig3-figsupp2-data2.zip › Figure 3-figure supplement 2-source data 2/Figure 3-figure supplement 2 repeat 3.pdf]

Nicotiana benthamiana

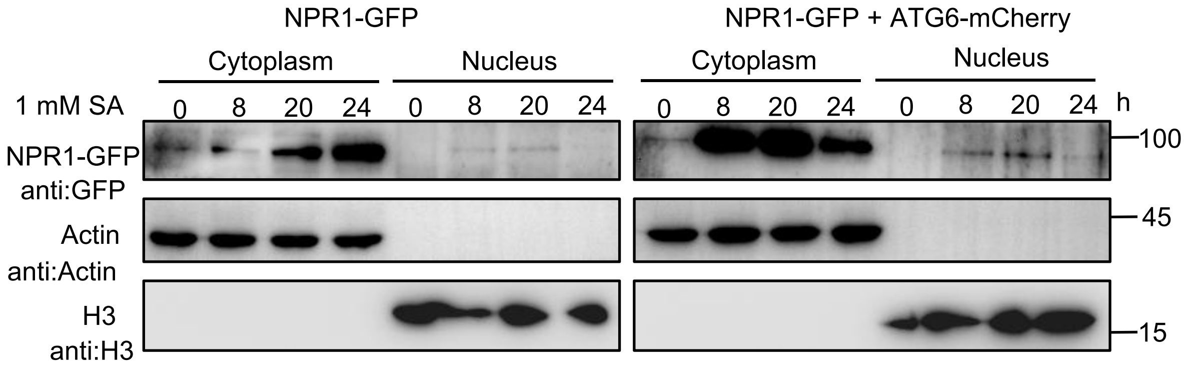

Fig 3e repeat 2

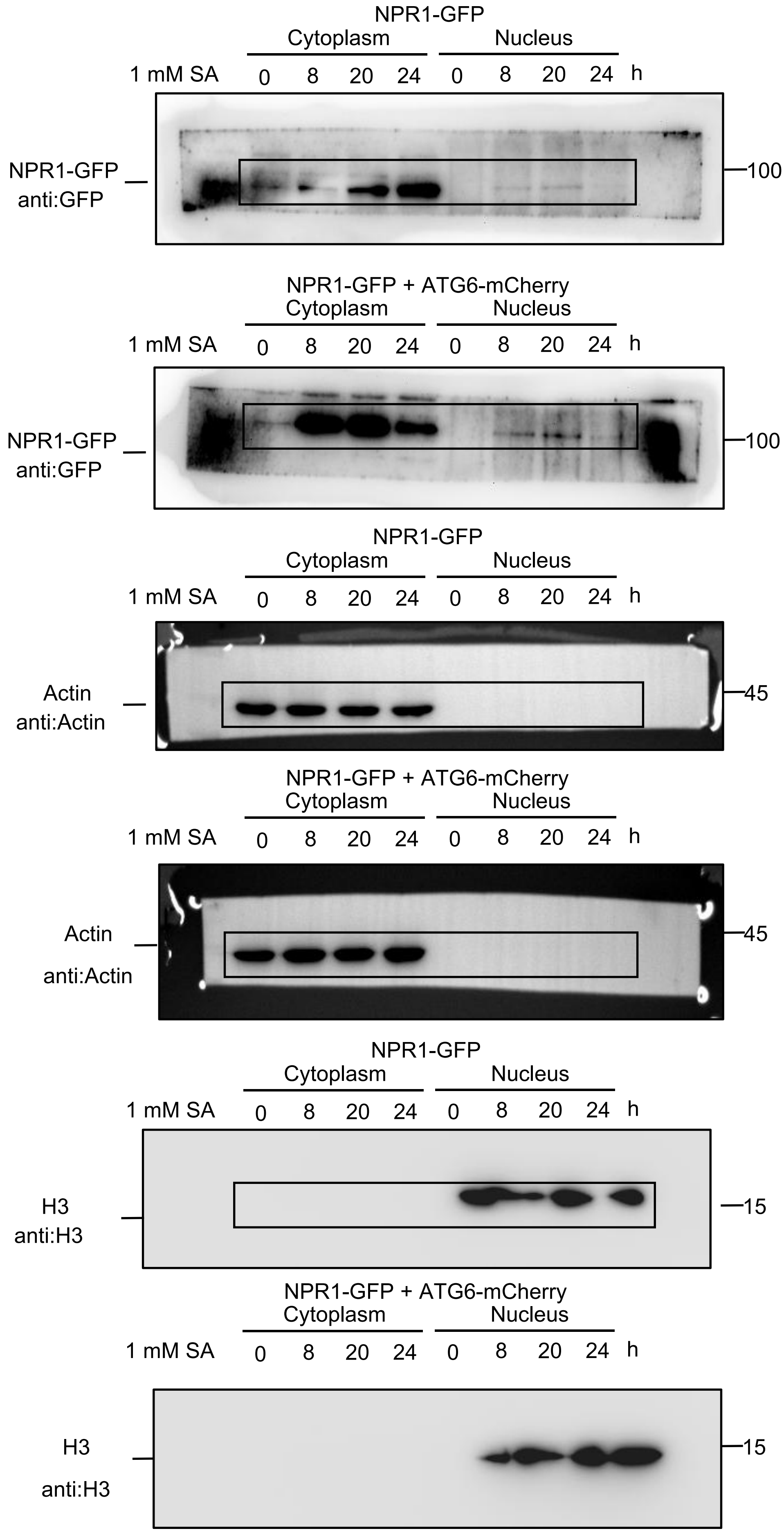

Figure 3-figure supplement 2 ATG6 increases the nuclear accumulation of NPR1 under SA treatment.
