# Supplementary material for: ATG6 interacting with NPR1 increases Arabidopsis thaliana resistance to Pst DC3000/avrRps4 by increasing its nuclear accumulation and stability
Source: eLife. 2025 Mar 4;13:RP97206. doi: 10.7554/eLife.97206 (PMC11879114; doi:10.7554/eLife.97206)
Supplement: Figure 5—figure supplement 1—source data 2. [file elife-97206-fig5-figsupp1-data2.zip › Figure 5-figure supplement 1-source data 2/Figure 5-figure supplement 1.pdf]

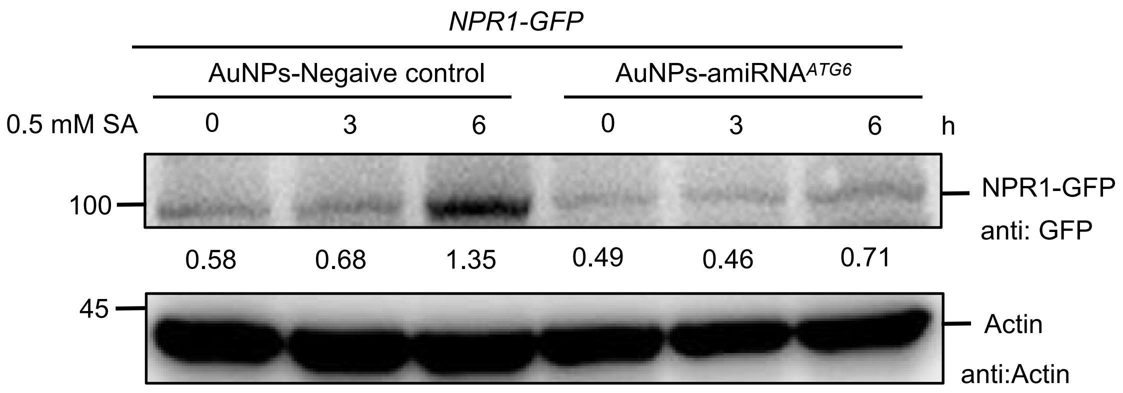

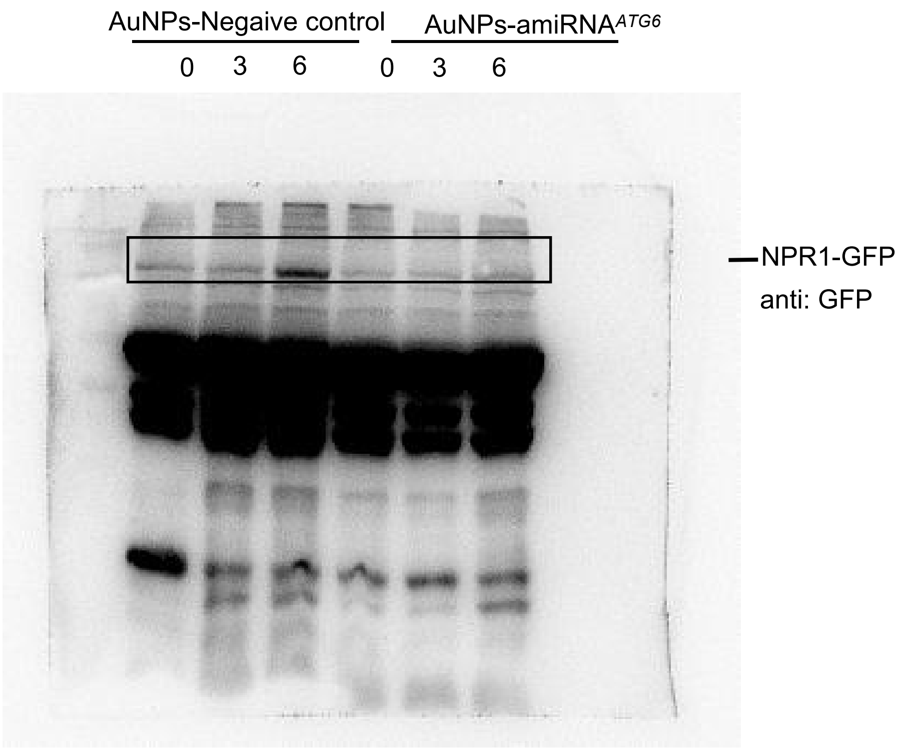

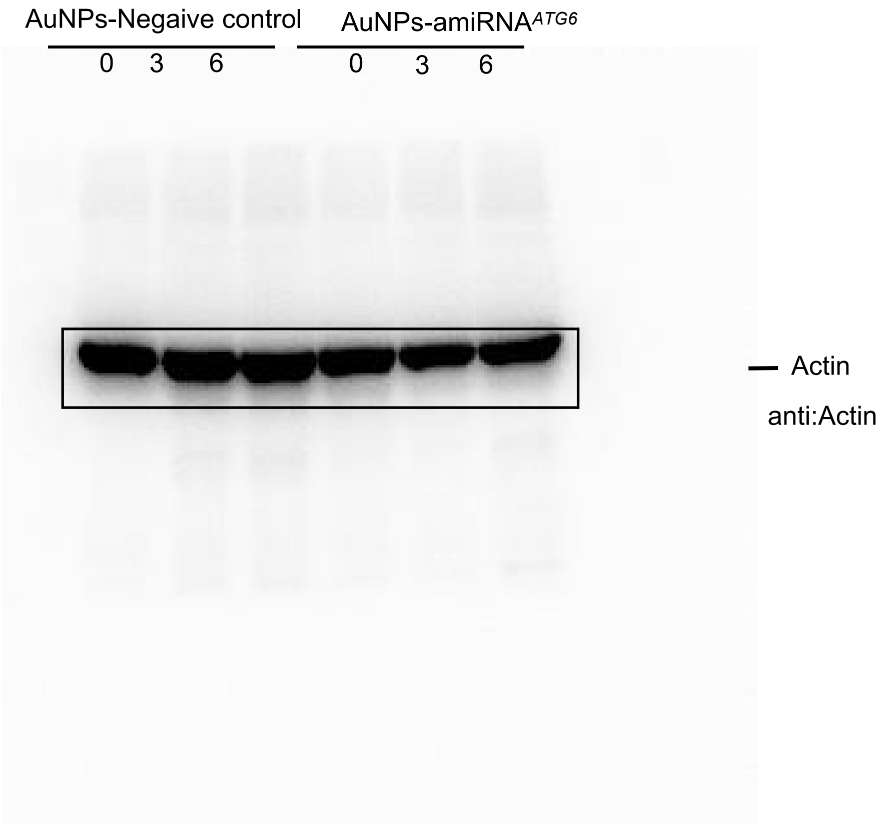

Figure 5-figure supplement 1 The protein level of NPR1-GFP in *NPR1-GFP*/silencing *ATG6* and *NPR1-GFP*/Negative control.
