# Supplementary material for: ATG6 interacting with NPR1 increases Arabidopsis thaliana resistance to Pst DC3000/avrRps4 by increasing its nuclear accumulation and stability
Source: eLife. 2025 Mar 4;13:RP97206. doi: 10.7554/eLife.97206 (PMC11879114; doi:10.7554/eLife.97206)
Supplement: Figure 2—figure supplement 2—source data 2. [file Figure 2-figure supplement 2a.pdf › Figure 2-figure supplement 2-source data 2/Figure 2-figure supplement 2a.pdf]

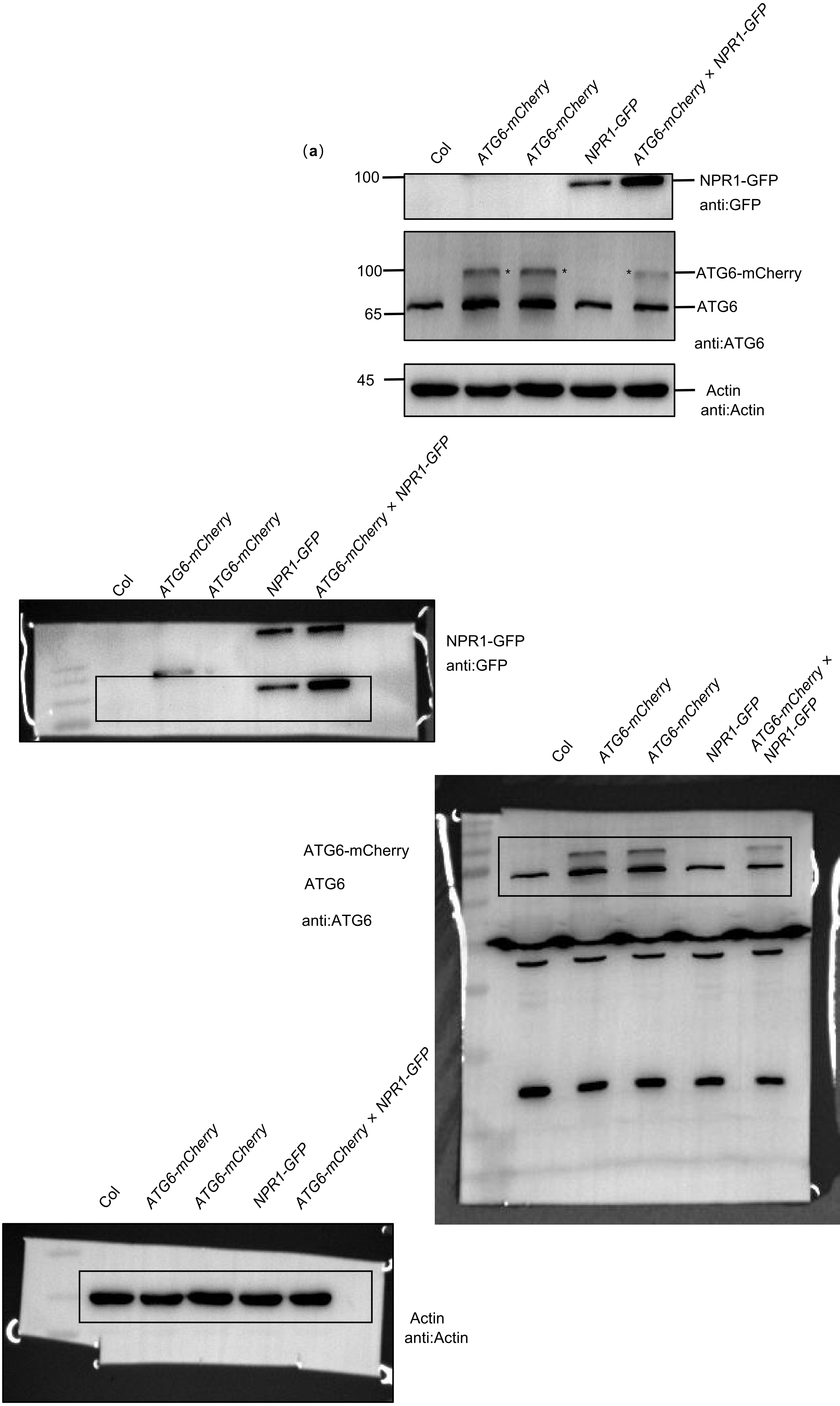

Figure 2-figure supplement 2 Identification of *ATG6-mCherry* × *NPR1-GFP* plants.
